# Safe and effective liver-directed AAV-mediated homology-independent targeted integration in mouse models of inherited diseases

**DOI:** 10.1016/j.xcrm.2024.101619

**Published:** 2024-06-18

**Authors:** Federica Esposito, Fabio Dell’Aquila, Manuel Rhiel, Stefano Auricchio, Kay Ole Chmielewski, Geoffroy Andrieux, Rita Ferla, Paula Sureda Horrach, Arjun Padmanabhan, Roberto Di Cunto, Simone Notaro, Manel Llado Santeularia, Melanie Boerries, Margherita Dell’Anno, Edoardo Nusco, Agnese Padula, Sofia Nutarelli, Tatjana I. Cornu, Nicolina Cristina Sorrentino, Pasquale Piccolo, Ivana Trapani, Toni Cathomen, Alberto Auricchio

**Affiliations:** 1 Telethon Institute of Genetics and Medicine (TIGEM), Pozzuoli, Italy; 2 Medical Genetics, Department of Advanced Biomedical Sciences, University of Naples Federico II, Naples, Italy; 3 Institute for Transfusion Medicine and Gene Therapy, Medical Center – University of Freiburg, Freiburg, Germany; 4 Center for Chronic Immunodeficiency (CCI), Medical Center – University of Freiburg, Freiburg, Germany; 5 PhD Program, Faculty of Biology, University of Freiburg, Freiburg, Germany; 6 Institute of Medical Bioinformatics and Systems Medicine, Medical Center – University of Freiburg, Freiburg, Germany; 7 Faculty of Medicine, University of Freiburg, Freiburg, Germany; 8 German Cancer Consortium (DKTK), Partner site Freiburg, a partnership between DKFZ and Medical Center - University of Freiburg, Freiburg, Germany; 9 Department of Life Science and Public Health, Catholic University of the Sacred Heart, Rome, Italy; 10 Department of Clinical Medicine and Surgery, University of Naples Federico II, Naples, Italy; 11 Gene Therapy Joint lab, Dept. of Advanced Biomedical Sciences and Dept. of Translational Medicine, University of Naples “Federico II”, Naples, Italy

**Keywords:** liver, *in vivo*, genome editing, AAV, CRISPR-Cas9, homology-independent targeted integration, HITI, inherited diseases, mucopolysaccharidosis type VI, hemophilia A, persistent transgene expression, CAST-Seq

## Abstract

Liver-directed adeno-associated viral (AAV) vector-mediated homology-independent targeted integration (AAV-HITI) by CRISPR-Cas9 at the highly transcribed albumin locus is under investigation to provide sustained transgene expression following neonatal treatment. We show that targeting the 3′ end of the albumin locus results in productive integration in about 15% of mouse hepatocytes achieving therapeutic levels of systemic proteins in two mouse models of inherited diseases. We demonstrate that full-length HITI donor DNA is preferentially integrated upon nuclease cleavage and that, despite partial AAV genome integrations in the target locus, no gross chromosomal rearrangements or insertions/deletions at off-target sites are found. In line with this, no evidence of hepatocellular carcinoma is observed within the 1-year follow-up. Finally, AAV-HITI is effective at vector doses considered safe if directly translated to humans providing therapeutic efficacy in the adult liver in addition to newborn. Overall, our data support the development of this liver-directed AAV-based knockin strategy.

## Highlights

• AAV-HITI provides sustained transgene expression from newborn and adult liver
• Liver-directed AAV-HITI is effective in mouse models of inherited diseases
• AAV-HITI results in undetectable off-targets and large gene rearrangements
• AAV-HITI is safe at the doses tested up to 1 year after AAV-HITI delivery

## Graphical abstract

**Figure:**
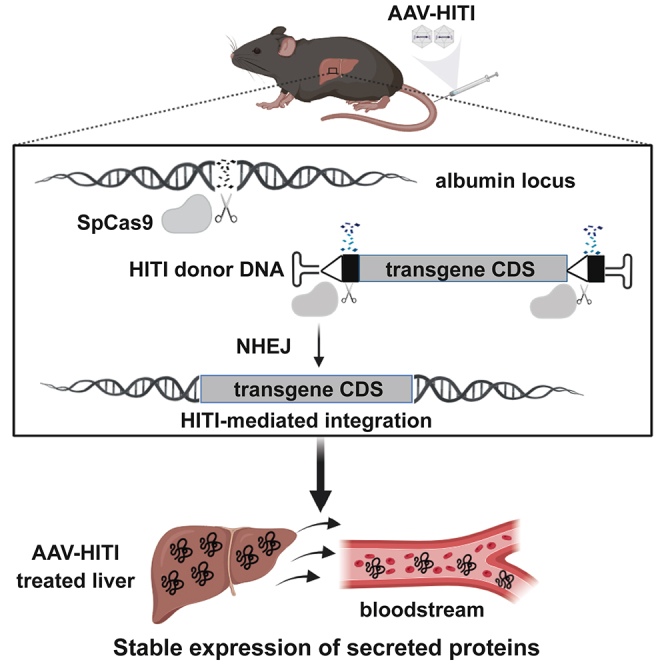

## Teaser

Esposito et al. explore the therapeutic potential of liver-directed adeno-associated viral vector-mediated homology-independent targeted integration (AAV-HITI) through CRISPR-Cas9 at the mouse albumin locus. Effective integration yields stable therapeutic protein levels in mouse models of inherited diseases without adverse effects, supporting AAV-HITI as a safe and efficient liver-directed knockin strategy.

## Introduction

Adeno-associated viral (AAV) vectors are considered the most effective tool for *in vivo* gene therapy due to their safety, efficacy, and long-term therapeutical transgene expression.^1^^,^^2^^,^^3^ In AAV-based liver-directed gene therapy, a single intravenous AAV administration has been demonstrated to be sufficient to convert hepatocytes into a factory for efficient and sustained transgene expression and secretion of proteins into the bloodstream, providing long-term therapeutic effects in both preclinical studies and humans.^4^^,^^5^^,^^6^^,^^7^^,^^8^^,^^9^ Despite their therapeutic potential, the non-integrative nature of AAV genomes excludes the use of AAV vectors from neonatal treatments,^10^^,^^11^^,^^12^^,^^13^ which is the preferred stage of intervention for diseases such as early-onset inborn errors of metabolism. Liver growth in young subjects leads to AAV genome loss^14^ over time resulting in the decline of the therapeutic efficacy following cell division.^1^ For this reason, patients under 4 years of age were excluded from our recent successfully completed phase 1/2 clinical trial of AAV-based liver gene therapy for mucopolysaccharidosis type VI (MPS VI)^5^ (Database: ClinicalTrials.gov, number NCT03173521). Furthermore, antibodies against the AAV vector capsid that are generated after the first administration may prevent a second round of treatment.^1^^,^^15^^,^^16^^,^^17^^,^^18^^,^^19^ To address these shortcomings, genome editing via CRISPR-Cas nucleases alone or in combination with knockin strategies is being explored extensively.^1^^,^^20^^,^^21^^,^^22^^,^^23^^,^^24^^,^^25^^,^^26^^,^^27^^,^^28^^,^^29^^,^^30^ In particular, homology-independent targeted integration (HITI) of therapeutic transgenes^21^^,^^25^^,^^31^^,^^32^^,^^33^^,^^34^ into the mouse albumin (m*Alb*) locus, which occurs via the cell cycle-independent non-homologous end-joining (NHEJ) repair machinery, is being considered to achieve stable expression following newborn liver gene therapy.^25^^,^^28^^,^^35^^,^^36^^,^^37^^,^^38^ Here, we show that AAV-HITI directed to the 3′ end of the albumin locus results in the integration of the donor DNA after its processing by CRISPR-Cas9 as expected based on HITI design. This provides significant therapeutic benefit in the absence of major off-target (OT) and toxic effects in different mouse models of inherited human diseases.

## Results

### Liver-directed AAV-HITI results in robust transgene expression following neonatal treatment

**Figure 1:**
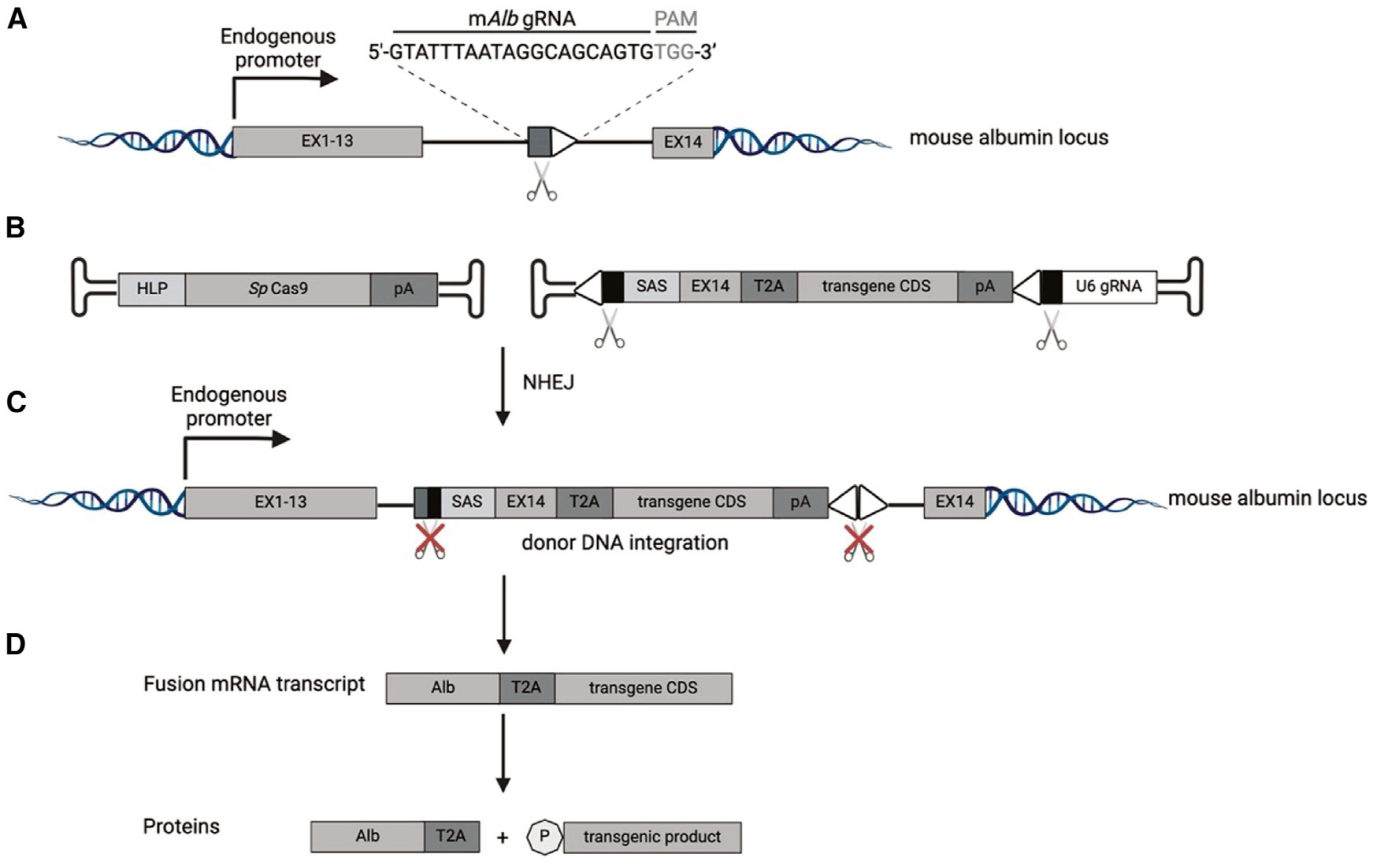
Schematic representation of homology-independent targeted integration (HITI) (A) The on-target mouse albumin locus and the gRNA sequence designed within the intron 13 are depicted. EX1-13, mouse albumin exons; EX14, last mouse albumin exon;PAM, protospacer adjacent motif. (B) Schematic representation of the two AAVs. One AAV carries the nuclease *Sp*Cas9 under control of the small hybrid liver-specific promoter (HLP). The other AAV contains the HITI donor DNA with the desired promoter-less transgene coding sequence (CDS). SAS, splicing acceptor signal; EX14, mouse albumin exon 14; T2A, Thosea asigna virus 2A skipping peptide; pA, polyadenylation signal (synthetic or bovine growth hormone); U6 gRNA, expression cassette for the gRNA (depicted) or the scRNA sequence; EX1-13, mouse albumin exons 1–13; gRNA and PAM sequences are represented with black (inverted gRNA sequences at the extremities of the donor DNA) and gray (within the mouse albumin locus) boxes and white triangle, respectively. (C) The non-homologous end-joining (NHEJ) repair pathway of the cell leads to the integration of the cleaved donor DNA at the on-target site. (D) Upon transgene integration a single fusion transcript is produced, and this results in the expression of both a modified albumin (Alb-2A) and a therapeutic transgene product containing a proline (P) residue at its N terminus.

**Figure 2:**
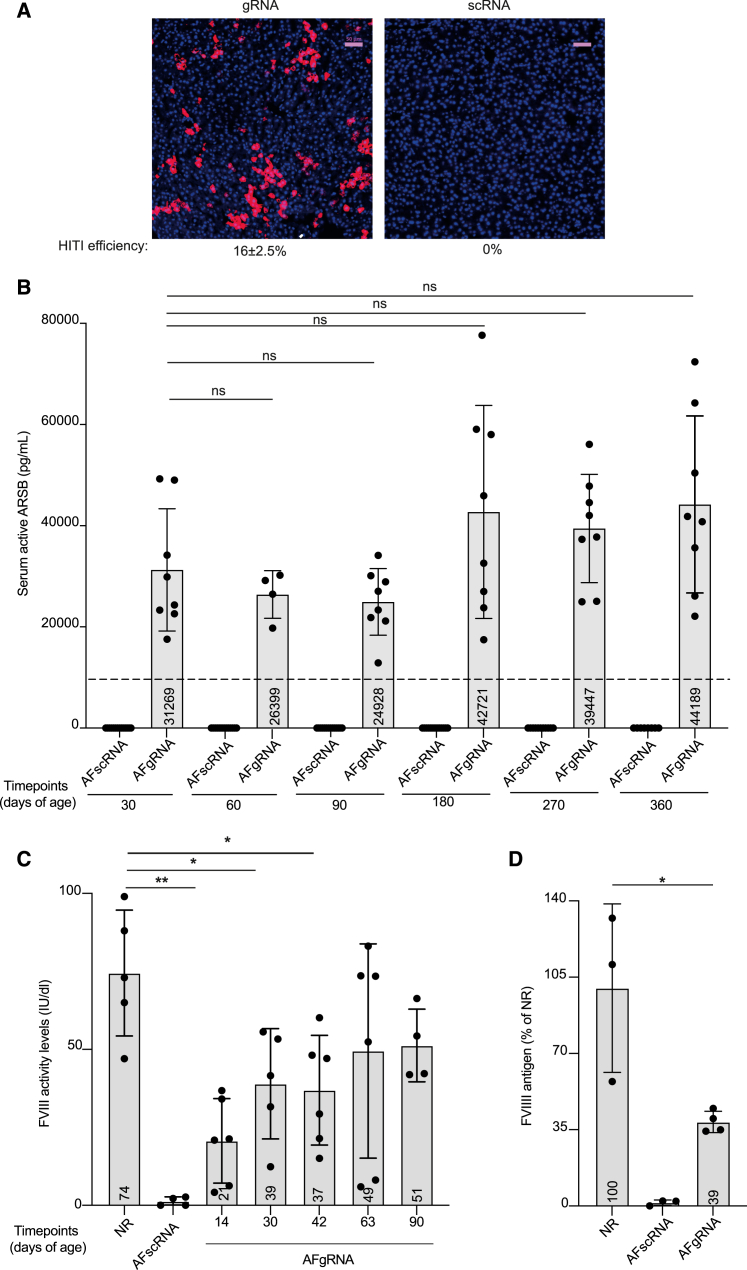
AAV-HITI-mediated transgene expression from newborn liver (A) Representative fluorescence microscopy images of OCT liver cryo-sections from wild-type mice injected with AAV-HITI using the dsRed transgene (gRNA, *N* = 5 or scRNA, *N* = 5) at the total dose of 1.2 × 10^14^ total GCs/kg. HITI efficiency is reported below the images. Scale bar, 50 μm. (B) Serum ARSB activity was analyzed at different time points after AAV-HITI treatment. AFgRNA, MPS VI mice treated with AAV-HITI-gRNA (*N* = 8); AFscRNA (*N* = 14 and *N* = 8 survived up to P360), MPS VI mice treated with AAV-HITI-scRNA. Dotted line corresponds to normal (NR) serum ARSB activity (NR = 11,825 ± 334 pg/mL; Alliegro et al., 2016^44^). ARSB measurements in all AFscRNA animals are equal to zero while all treated mice show ARSB activity levels higher than zero; therefore all the comparisons between the two groups are significant. No statistically significant differences were observed in AFgRNA-treated mice among the different time points. (B–C) Each dot corresponds to a single animal within each group at different time points. (C) FVIII activity levels evaluated in HemA mice by chromogenic assay at different time points after AAV-HITI treatment. Statistical differences were assessed by ordinary one-way ANOVA test. ∗∗*p* = 0.0025, between normal (NR, *N* = 5) and AFgRNA (*N* = 6) at 14 days; ∗*p* = 0.0190, between NR and AFgRNA (*N* = 5, one sample was excluded) at 30 days; ∗*p* = 0.0389, between NR and AFgRNA (*N* = 6) at 42 days. No statistically significant differences were observed between NR and AFgRNA at the following time points. (D) FVIII antigen levels measured in AFgRNA (*N* = 4) HemA mice at 90 days of age. ∗*p* = 0.0262 between NR and AFgRNA at 90 days. All data are represented as mean ± standard deviation. See also Figures S1, S2, and Table S1.

Our AAV vector-based CRISPR-Cas9-mediated HITI (AAV-HITI) approach was designed to target intron 13 of the m*Alb* locus, similarly to previous reports^34^ (Figure 1A). The system relies on the co-delivery of two different AAV8 vectors. AAV8 has high liver tropism^39^^,^^40^ and has been safely and effectively used in various AAV-liver-directed genome editing preclinical studies^21^^,^^30^^,^^41^ as well as by us and others in liver-directed gene therapy clinical trials.^5^^,^^8^ One AAV delivers the large *S. pyogenes* Cas9 (*Sp*Cas9) nuclease together with a small hybrid liver-specific promoter (HLP),^21^^,^^42^^,^^43^ while the other carries the HITI donor DNA which is designed to contain a synthetic splicing acceptor signal (SAS) followed by the last m*Alb* exon (exon 14), the Thosea asigna virus 2A skipping peptide (T2A), and the promoter-less coding sequence (CDS) of a desired transgene together with a polyadenylation signal (pA) and the guide (g) RNA (or scramble RNA [scRNA]) expression cassette including the U6 promoter (Figure 1B). As previously described,^21^^,^^32^^,^^33^ the HITI donor DNA is flanked at its 5′ and 3′ extremities by the same gRNA sequence of the endogenous target site (m*Alb* intron 13) but in an inverted orientation. Upon CRISPR-Cas9-mediated cleavage at both the endogenous locus and the extremities of the HITI donor DNA, the NHEJ repair pathway of the cell promotes donor DNA integration at the nuclease-induced double-strand breaks (DSBs) in the endogenous locus^21^^,^^32^^,^^33^^,^^34^ (Figure 1C). Once proper integration has occurred, the therapeutic transgene is expressed under the control of the endogenous promoter. A single fusion mRNA transcript is generated; at the protein levels, this will result in the production of both a modified albumin (Alb-2A) and a therapeutic transgene product containing a proline (P) residue at its N terminus (Figure 1D). To evaluate the efficiency of the system at targeting newborn hepatocytes, we used an AAV-HITI donor carrying the CDS of the *Discosoma* sp*.-Red* (dsRed) fluorescent protein together with the expression cassette encoding either the gRNA or an scRNA which served as a negative control (Table S1). Wild-type C57BL/6 mice were divided into two different treatment groups (gRNA or scRNA) and received a mixture of vectors at 1:1 ratio via temporal vein injection between post-natal days (P) 1 and 2 at a total dose of 1.2 × 10^14^ genome copies (GCs)/kg. Four weeks post-injection, fluorescent microscopy images from liver cryo-sections revealed that dsRed was highly expressed in livers of mice treated with gRNA (mean ± SD = 16% ± 2.5% dsRed-positive hepatocytes; Figures 2A and S1A) but not with scRNA. This indicates that our AAV-HITI approach provides robust transgene expression following neonatal delivery in mice. We then investigated the persistence of the AAV-HITI donor DNA following newborn delivery and found a significant drop after a month, as expected based on hepatocyte proliferation at that age, with similar very low levels at 1 and 12 months after vector delivery (Figure S1B). We therefore hypothesized that the majority of the HITI donor DNA at 1 month after newborn delivery is integrated and performed a customized DNA hybridization analysis with a probe specific for the HITI donor DNA vector on liver cryo-sections to assess the rate of its integration. We found about 40% more positive hepatocyte nuclei in gRNA- than scRNA-treated samples, presumably resulting from HITI donor DNA integration whether full or partial (Figure S1C).

We next investigated the therapeutic potential of our strategy in two different mouse models of human inherited diseases. Liver-directed AAV-HITI efficacy was first assessed in a mouse model of MPS VI,^45^ a lysosomal storage disorder due to arylsulfatase B (ARSB) deficiency. In this setting, the AAV-HITI donor vector contained the *ARSB* CDS in place of dsRed (Table S1). AAV-HITI vectors were co-delivered systemically in newborn MPS VI mice as described earlier, and the mice were monitored over a period of 1 year. Since ARSB is secreted into the bloodstream, it can be measured non-invasively^45^ as a readout for AAV-HITI treatment. Starting from 1-month post-treatment, serum ARSB activity was found to be higher in AAV-HITI-gRNA-treated MPS VI (AFgRNA) than in wild-type mice^44^ (Figure 2B) while AAV-HITI-scRNA-treated littermates (AFscRNA) had undetectable levels of ARSB at all time points (Figure 2B). Interestingly, ARSB activity in AFgRNA mice showed a non-statistically significant trend toward increase over time (Figure 2B). Next, we investigated whether following AAV-HITI serum albumin includes the T2A modification (Alb-2A) as predicted based on the AAV donor DNA design (Figure 1). Western blot analysis of AAV-HITI-treated sera samples collected at different time points confirmed the presence of Alb-2A of correct size (Figure S2A). In addition to MPS VI, AAV-HITI efficacy was assessed in a mouse model of hemophilia A (HemA), the most common X-linked bleeding disorder caused by mutations in the *F8* gene.^46^ Since the full-length *F8* CDS (∼7 kb) exceeds the cargo capacity (∼4.9 kb) of the AAV vector, we designed an HITI donor DNA carrying the promoter-less CDS of a previously described human B domain-deleted (BDD) *F8* variant^47^ (CodopV3 ∼4.5 kb; Table S1). Newborn HemA male mice were injected systemically at P1–2 with AAV-HITI vectors (gRNA or scRNA) at a total dose of 3.9 × 10^13^ GCs/kg. Plasma samples were collected from AAV-HITI-treated HemA mice (AFgRNA and AFscRNA) and unaffected controls at different time points, and FVIII activity and FVIII antigen were measured. Therapeutic levels of FVIII were observed in plasma samples of AFgRNA- but not AFscRNA-treated mice up to 90 days of age by chromogenic assay^42^^,^^48^ (Figure 2C), and FVIII protein levels were found to be around 40% of normal FVIII levels at 90 days post AAV-HITI delivery (Figure 2D). Lastly, Alb-2A expression was confirmed in AFgRNA mice at different time points by western blot analysis (Figure S2B).

### Liver-directed AAV-HITI improves the phenotype of MPS VI and HemA mice

**Figure 3:**
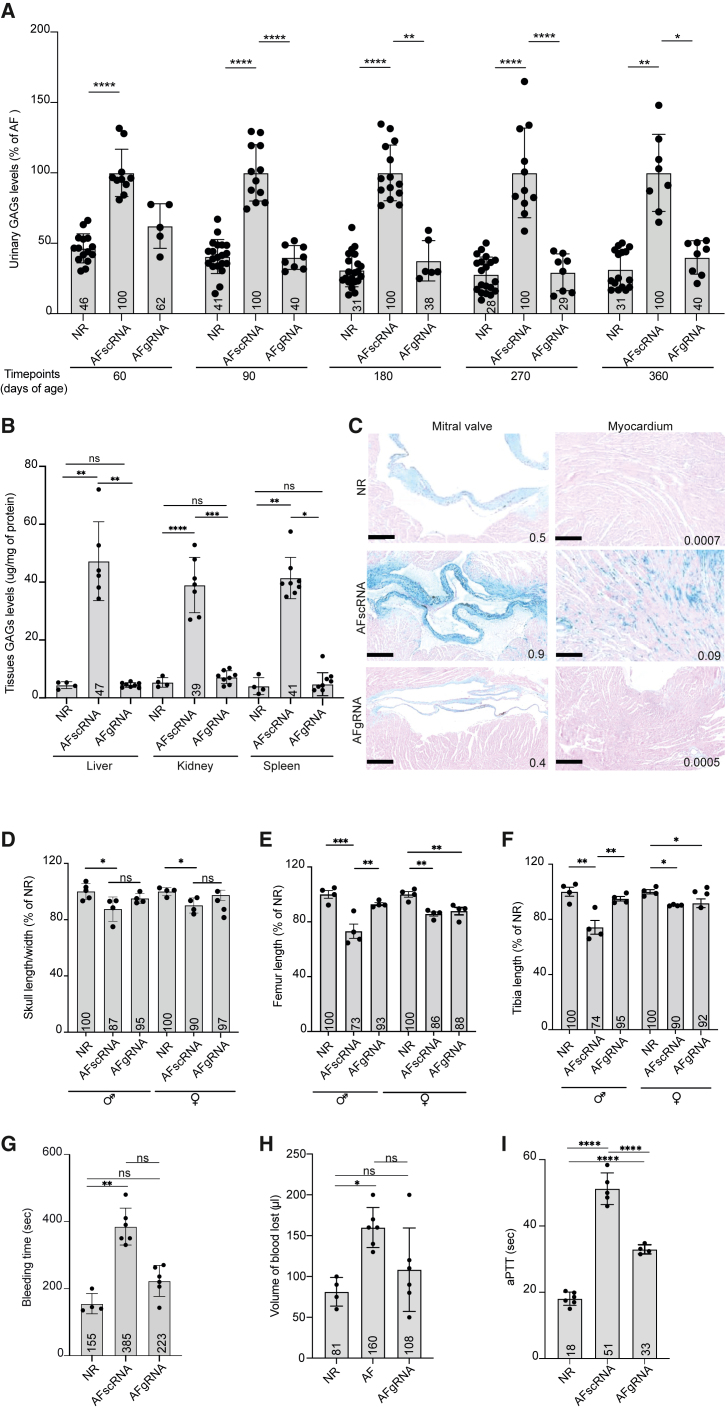
Liver-directed AAV-HITI therapeutic efficacy in newborn mice (A) Urinary levels of glycosaminoglycans (GAGs) reported as a percentage of GAG levels in affected MPS VI mice (% of AF). Statistical differences were assessed by Kruskal-Wallis test and Dunn’s multiple comparisons test. *p* values: ∗*p* = 0.0379, ∗∗*p* = 0.0022, and ∗∗∗∗*p* < 0.0001. AFgRNA (*N* = 8), MPS VI mice treated with AAV-HITI-gRNA; AFscRNA (*N* = 14 and only *N* = 8 survived up to P360), MPS VI mice treated with AAV-HITI-scRNA. Each dot corresponds to a single animal within each group at different time points. (B) Quantification of GAGs in the liver, kidney, and spleen. Statistical differences were assessed by Brown-Forsythe and Welch ANOVA tests. For the liver: ∗∗*p* = 0.0016 between NR (*N* = 4) and AFscRNA (*N* = 6); ∗∗*p* = 0.0016 between AFscRNA and AFgRNA (*N* = 8); *p* = 0.9996 between NR and AFgRNA. For the kidney: ∗∗∗∗*p* = 0.0001 between NR (*N* = 4) and AFscRNA (*N* = 7); ∗∗∗*p* = 0.0002 between AFscRNA and AFgRNA (*N* = 8); *p* = 0.3784 between NR and AFgRNA. For the spleen the Kruskal-Wallis test was used: ∗∗*p* = 0.0029 between NR (*N* = 4) and AFscRNA (*N* = 8); ∗*p* = 0.0113 between AFscRNA and AFgRNA (*N* = 8); *p* > 0.9999 between NR and AFgRNA. (A and B) Each dot corresponds to a single animal within each group at different time points. The differences in the number of analyzed samples within the same group of treatment at different time points were due to sample availability. (C) Representative histological images of GAG storage in mitral heart valve and myocardium. Scale bar, 100 μm. Alcian blue quantification is reported inside the images as Alcian blue-positive area/total area. NR, *N* = 4; AFscRNA, *N* = 8; AFgRNA, *N* = 8. (D–F) Measurement of skull length/width ratio, femur, and tibia lengths; data are reported as the percentage of normal length (% of NR). Males and females were kept separate in the analysis. AFgRNA, *N* = 8; AFscRNA, *N* = 8. Statistical differences were assessed by ordinary one-way ANOVA and Tukey’s multiple comparisons test. (D) *p* value ∗ = 0.0336 between NR and AFscRNA males; *p* = 0.4847 between NR and AFgRNA males; ∗*p* = 0.0191 between NR and AFscRNA females; *p* = 0.6887 between NR and AFgRNA females. (E) ∗∗∗*p* = 0.0011 between NR and AFscRNA males; *p* = 0.4491 between NR and AFgRNA males; ∗∗*p* = 0.0099 between AFscRNA and AFgRNA males; ∗∗*p* = 0.0122 between NR and AFscRNA females; ∗∗*p* = 0.0055 between NR and AFgRNA females. (F) ∗∗*p* = 0.0019 between NR and AFscRNA males; ∗∗*p* = 0.0089 between NR and AFgRNA males. ∗*p* = 0.0238 between NR and AFscRNA females; ∗*p* = 0.0398 between NR and AFgRNA females. (G and H) Tail-clip assay performed at 63 days of age in HemA AAV-HITI-treated male mice (AFgRNA, *N* = 6; AFscRNA, *N* = 6) and unaffected (NR; *N* = 4) controls. (G) ∗∗*p* = 0.0019; (H) ∗*p* = 0.0129. (I) Activated partial thromboplastin time (aPTT) measured at 90 days of age in HemA AAV-HITI (AFgRNA, *N* = 4 and AFscRNA, *N* = 5) mice and unaffected (NR, *N* = 6) controls. *p* values: ∗∗∗∗*p* < 0.0001 between NR and AFscRNA; ∗∗∗∗*p* < 0.0001 between NR and AFgRNA; ∗∗∗∗*p* < 0.0001 between AFscRNA and AFgRNA. All data are represented as mean ± standard deviation. See also Figure S3.

ARSB deficiency results in systemic abnormal storage and urinary excretion of glycosaminoglycans (GAGs). The measurement of urinary GAGs levels is considered a useful biomarker for MPS VI.^5^^,^^49^^,^^50^ To evaluate AAV-HITI-mediated phenotypic improvement, we collected urine samples from treated mice and unaffected littermates at different time points. Normalized urinary GAG levels were found in urinary samples from AFgRNA-treated MPS VI mice starting from 3 months post-treatment (P90) until the last time point of the analysis (P360; Figure 3A). One-year post-treatment, MPS VI mice were sacrificed, and different organs were collected for further analysis. GAG levels in liver, kidney, and spleen lysates were normal in AFgRNA-treated MPS VI mice (Figure 3B). Accordingly, GAGs storage was significantly decreased in histological sections of these tissues (Figure S3) as well as in the heart mitral valve and myocardium of the AFgRNA-treated MPS VI mice (Figure 3C). Indeed, MPS VI patients suffer from heart failure, predominantly due to mitral valve insufficiency.^51^ A further feature of MPS VI patients is skeletal abnormalities,^49^^,^^52^ which have also been described in the mouse model of MPS VI.^53^ Therefore, we assessed if AAV-HITI can improve these disease manifestations. Radiographic images from the AAV-HITI-treated MPS VI mice (AFgRNA or AFscRNA) and unaffected normal controls (NR) were used to evaluate the skull and long bones (femurs and tibias); male and female mice were considered separately given the sex-dependent growth differences. In AFgRNA-treated mice we observed an improvement in the skull width/length ratio compared to AFscRNA-treated mice of the same sex (Figure 3D). In AFgRNA-treated male mice we found a significant amelioration in the femur (Figure 3E) and tibia (Figure 3F) lengths. Altogether, our data indicate that AAV-HITI is effective following newborn delivery to MPS VI mice. Lastly, we assessed AAV-HITI efficacy in newborn AAV-HITI-treated (AFgRNA or AFscRNA) HemA mice by measuring the hemostasis activity by tail-clip and activated partial thromboplastin time (aPTT) assays.

We found that AFgRNA-treated male mice exhibited a significant reduction of bleeding time (Figure 3G) and blood loss (Figure 3H) at 63 days of age and improved coagulation compared to AFscRNA-treated mice at 90 days of age (Figure 3I). These findings support the use of AAV-HITI for therapies directed to newborn liver.

### AAV-HITI at the on-target site

**Figure 4:**
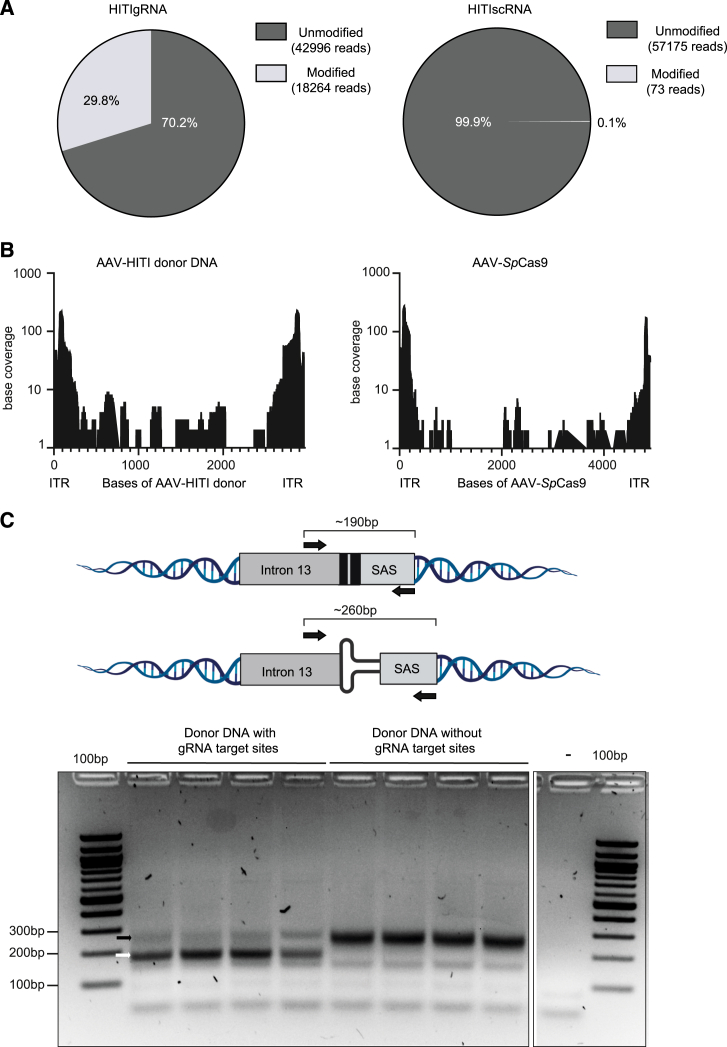
AAV-HITI molecular characterization at the on-target site (A) Pie chart showing the modified reads observed in AAV-HITI (HITIgRNA or HITIscRNA)-treated mice analyzed by next-generation sequencing (NGS). The expected amplicon size is 285 bp, which was covered by 2x250 bp reads in NGS. The percentage of the modified reads is used as an indication of the gRNA efficiency at the on-target site (HITIgRNA∼30% of indel). (B) Sequences from different portions of both AAV vectors (AAV-HITI donor DNA and AAV-*Sp*Cas9) are captured at the induced double-strand breaks, mostly ITR sequences (ITR). (C) Schematic representation of the strategy with the expected PCR amplicons and the position of the primers (up) and representative images of the 5′ junction PCR run on a 2% agarose gel (down). Four different biological samples for each group are showed in the gel. The white arrow indicates proper HITI-mediated donor DNA integration band (∼190 bp); the black arrow indicates the ITR-mediated donor DNA integration band (∼260 bp). See also Table S1.

Having established the physiological benefits of AAV-HITI in two disease models, we thoroughly characterized AAV-HITI at the on-target site by molecular analysis and next-generation sequencing (NGS). Genomic DNA extracted from liver samples of AAV-HITI-treated MPS VI animals was used to evaluate *Sp*Cas9-gRNA efficiency 1-year post-treatment. For each treatment (gRNA or scRNA), a pool of 3 different DNA samples was analyzed by targeted amplicon NGS. Small insertions/deletions (indels) were observed in ∼30% of reads from gRNA but not scRNA samples (∼0.1%; Figure 4A). Interestingly, in ∼1% (*n* = 603) of the total obtained reads (*n* = 61,260) from the gRNA DNA pool, we observed insertions longer than 10 nucleotides which were found to align with the AAV-HITI donor and the AAV-*Sp*Cas9 vector genomes. The vast majority of these insertions originated from the inverted-terminal repeat (ITR) regions (Figure 4B). We then investigated if the AAV donor DNA was integrated before or after being processed by CRISPR-Cas9 (therefore with or without the ITRs, respectively). To this end, we generated, as control, a donor DNA lacking the inverted gRNA sites at both its 5′ and 3′ extremities (donor DNA without gRNA target sites; Table S1). This donor should be integrated exclusively with its ITRs. Next, wild-type newborn C57BL/6 mice were randomly assigned to two different treatment groups and injected systemically by temporal vein at P1–2 at a total dose of 3.9 × 10^13^ GCs/kg. The same AAV vector encoding for the nuclease (AAV-*Sp*Cas9) was used in both groups and co-delivered with a second AAV carrying either the previously described (Figure 1B) dsRed HITI donor DNA (HITI donor) or the newly generated donor DNA without gRNA target sites. One-month post-treatment, genomic DNA was extracted from liver samples for molecular analysis. The 5′ junction was PCR amplified with specific primers (Figure 4C; Table S2) on DNA samples obtained from all the treated mice. A PCR product corresponding to the expected size (∼260 bp) of the ITR-mediated integration was observed in samples treated with the donor DNA without gRNA target sites and the presence of ITRs confirmed by Sanger sequencing. Two junction PCR products of different sizes (∼260 and ∼190 bp; Figure 4C) were found in the samples from the AAV-HITI group. Sanger sequencing analysis showed that the smaller and more abundant PCR product (∼190 bp) corresponded to the expected HITI-mediated integration while the higher (∼260 bp) and less represented PCR product to the result of ITR-mediated integration.

**Figure 5:**
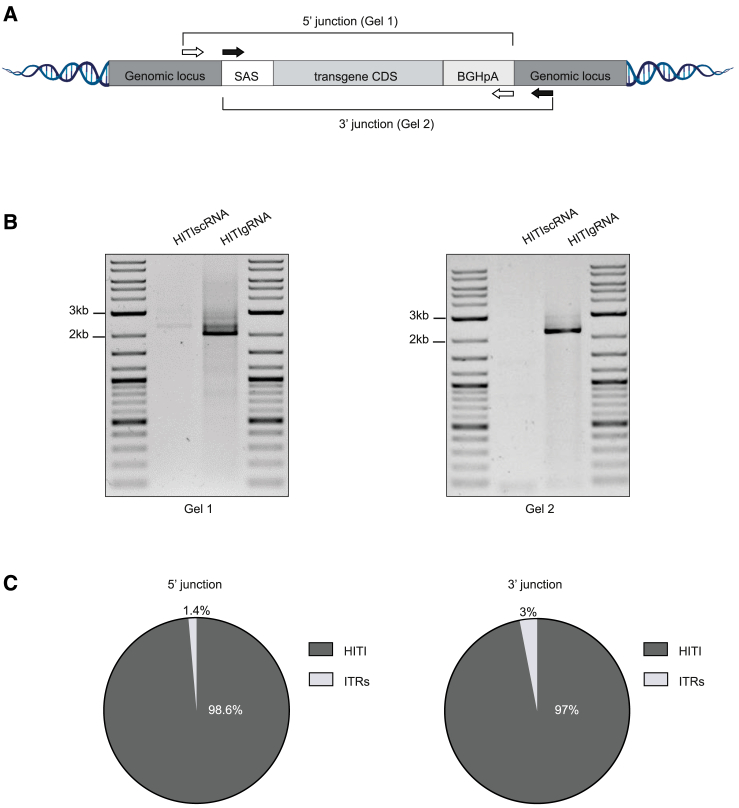
Long-reads analysis shows that full-length HITI donor DNA is integrated predominantly after ITR cleavage (A) Schematic of the different long PCR junction products. White arrows indicate the primers designed to amplify the 5′ junction: the forward primer (white left) was designed in the endogenous locus right before the cleavage site; the reverse primer (white right) was designed at the end of the donor DNA on the polyadenylation signal (BGHpA). Black arrows indicate the primers designed to amplify the 3′junction: the forward primer (black left) was designed at the beginning of the donor DNA on the splicing acceptor signal (SAS); the reverse primer (black right) was designed in the endogenous locus right after the cleavage site. (B) Representative images of the 5′ and 3′ junction PCR products run on an agarose gel for the detection of the full-length HITI donor DNA (∼2 kb). The 5′ junction is showed in Gel 1 and the 3′ junction in Gel 2. (C) Pie chart showing the percentage (%) of long reads in which donor DNA integration was HITI mediated or ITRs mediated (ITRs) at the junction sites. See also Figures S4, S6, and Tables S2 and S3.

To further evaluate the role of AAV ITRs in mediating full-length HITI donor DNA integration, we analyzed DNA samples from AFgRNA-treated MPS VI mice (previously injected to assess AAV-HITI efficacy) by long-read (LR) sequencing. We initially tried to amplify the target site upon HITI integration, hoping to detect the full-length integrated donor DNA. However, we obtained only small PCR products resembling the target site without HITI donor integration. This is very likely due to the strong bias toward amplification of smaller fragments during PCR. Here, the fragment derived from wild-type alleles is ∼10-fold smaller than the expected fragment after HITI. In order to circumvent this PCR bias yet visualize the target site after successful HITI, we PCR-amplified in separate reactions two different long PCR products (5′ and 3′junctions; Figure 5A) whose size corresponded to the full-length donor DNA integrated at the on-target (∼2 kb; Figure 5B). These long PCR fragments were analyzed by LR sequencing using Nanopore technology with specific primers (Table S3). We found that the majority of the obtained reads corresponded to the expected HITI-mediated integration; however, a small fraction of reads presented with remnants of ITRs. We then tailored a bioinformatics pipeline to count the reads that contained ITR sequences, and we found that 1.4% of the 5′ junction reads and 3% of the 3′ junction reads contained substantial (>9 bp) parts of the ITR sequences (Figure 5C). To assess the frequency of the ITR-mediated donor DNA integration by a method complementary to LR sequencing, we performed Illumina-based short-read sequencing on samples treated with AAV-HITI. Short fragments (∼200 bp) covering the 5′ junction were produced, and the number of reads containing (partial) ITRs was counted. Across three samples, we found that on average 9.8% (range 8.3%–11.3%) of the reads contained substantial (>9 bp) parts of an ITR. In line with the LR sequencing data, the vast majority of integrated fragments seem to have been released from the viral sequences prior to integration via HITI (Figure S4). To assess the various HITI donor DNA integration outcomes, we performed chromosomal aberrations analysis by single targeted linker-mediated PCR sequencing (CAST-Seq)^54^ analysis (Figure S5) of liver DNA samples. CAST-Seq revealed six predominant integration events (Figure S6A): (1) the correct HITI event in the desired orientation (peak 1), (2) a fusion of intron 13 with the pA-encoding region of the AAV vector (peak 2), and (3) ITR-containing AAV fragments in both forward and reverse orientations (peaks 3–6; Figure S6A). Based on read coverage, we estimate that the intended HITI fusion product accounts for about 20% of integration events which is slightly higher than the integration in the reverse orientation (14.5%). The most frequent integration event is capturing of ITR-containing AAV fragments, maybe because they are shorter than the transgene-containing DNA sections (Figures S6A and S6B). In Figure S6C, only a sub-fraction of CAST-Seq reads was analyzed, i.e., only reads representing integration of cleaved (ITR-free) HITI donor template. This analysis includes peak 1 (19.7% of all integration events) and peak 2 (14.5% of all integration events) of Figures S6A and S6B but none of the other peaks (peaks 3–6). Of the total 34.2% (i.e., 19.7% + 14.5%) ITR-free HITI donor integration events, 48% are productive (Figure S6C). These 16.4% (48% of 34.2%) align very well with the 16% dsRed-positive hepatocytes observed by fluorescence microscopy (Figure 2A). Furthermore, the 34.2% of HITI integration events converge with the 49% HITI-positive nuclei observed in Figure S1C.

### Safety of AAV-HITI following neonatal delivery

To assess the safety of AAV-HITI following neonatal treatment, we evaluated over time the amount of AAV-*Sp*Cas9 genomes following delivery in newborn mice and found a significant drop of about 30-fold after a month (Figure S7A), which is in line with data in Figure S1B.

To further investigate AAV-*Sp*Cas9 genome integration at the target site, we used CAST-Seq. We observed predominantly ITR-mediated capture of the AAV-*Sp*Cas9 genome without preference for either ITR (Figure S7B); however, Cas9 integration events were found to be less abundant (peak at 60,000 reads in Figure S7B) than HITI donor DNA integration events (peak at 200,000 reads in Figure S6A). Detection of internal AAV regions, in addition to the ITRs, suggests that internal regions were likely captured upon end resection of the vector genome (Figure S7B). To qualitatively assess potential full-length AAV-*Sp*Cas9 integration at the on-target site, we used specific primers as depicted in Figure S7C (Table S4). A faint PCR fragment corresponding to the expected size (∼4.5 kb) was amplified in the AFgRNA- but not in the AFscRNA-treated sample (Figure S7C). Full-length *Sp*Cas9 integration was confirmed by Sanger sequencing analysis. To understand whether this could result in *Sp*Cas9 expression, we performed western blot analysis on AAV-HITI-treated liver lysates 1 year after vector delivery, and no Cas9 expression was detected (Figure S7D) suggesting that the risk of unwanted genetic editing due to long-term *Sp*Cas9 expression appears low under these conditions.

**Figure 6:**
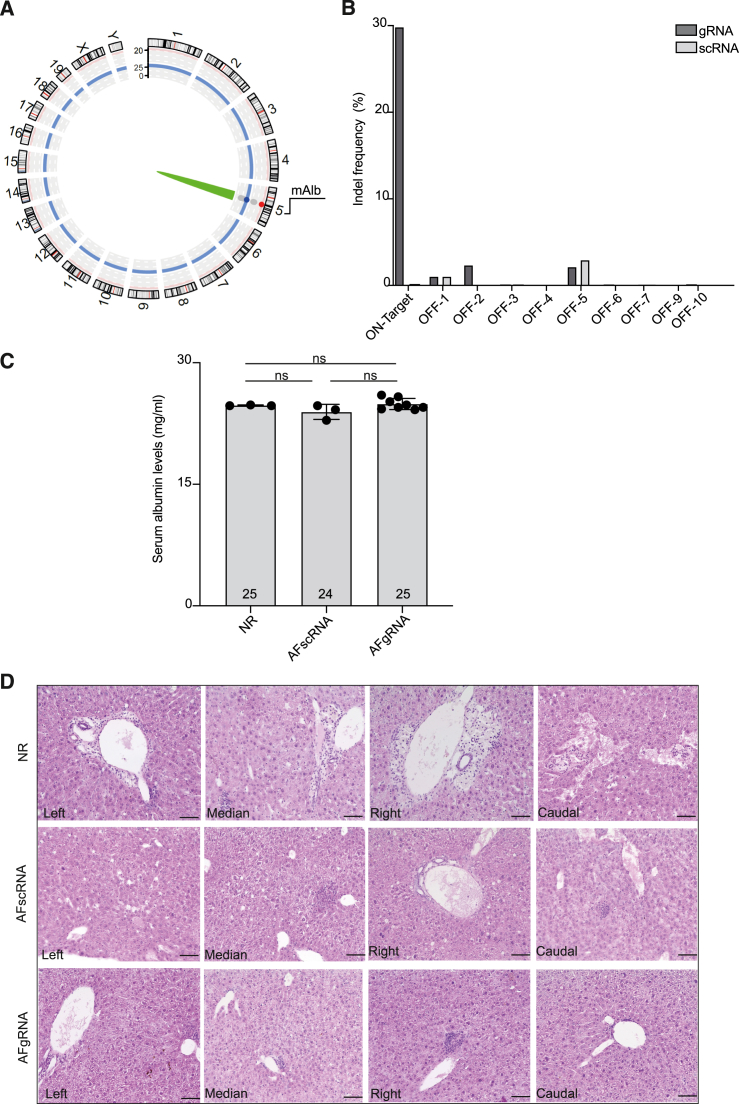
Safety following neonatal delivery of high doses of AAV-HITI (A) The circos plot summarize the CAST-Seq analysis performed on genomic DNA extracted from liver samples of AAV-HITI-treated MPS VI mice. The on-target is reported in green. No chromosomal aberrations were found. (B) Off-target activity (indel frequency) measured at the top predicted off-target loci by next-generation sequencing (NGS) analysis on genomic DNA extracted from liver samples of AAV-HITI-treated MPS VI. The off-target 8 (OFF-8) was excluded due to technical issues. (C) Serum albumin levels measured 1-year post-treatment in sera samples from normal controls (NR, *N* = 3) or AAV-HITI-treated MPS VI mice (AFgRNA, *N* = 8; AFscRNA, *N* = 3); data are represented as mean ± standard deviation. Statistical differences were assessed by ordinary one-way ANOVA test. (D) Representative images from histopathological analysis performed on paraffin sections from different liver lobes from AAV-HITI-treated MPS VI mice (AFgRNA, *N* = 8; AFscRNA, *N* = 8) and untreated normal controls (NR, *N* = 4) 1-year post-treatment. Scale bar, 50 μm. See also Figures S7, S8 and Tables S5–S7.

To evaluate potential CRISPR-Cas9-induced chromosomal rearrangement in an unbiased fashion, we performed CAST-Seq designed to this aim. We have shown before that CAST-Seq allows for the nomination of off-target (OT) sites through detection of OT-cleavage-induced translocations.^54^ It furthermore enables assessment of DNA aberrations at the target site post nuclease treatment, including large deletions and inversions. CAST-Seq performed on genomic DNA extracted from liver samples of 3 different MPS VI mice treated with AAV-HITI (gRNA or scRNA; Table S5) revealed an exquisite safety profile of our nuclease. We did not detect any OT-mediated translocations (OMTs; Figure 6A) but only large deletions/inversions surrounding the on-target site (Figure S8A), in extreme cases reaching up to 35 kb. CAST-Seq also allowed us to assess the relative frequencies of gross chromosomal aberrations at the target site. We found that 2.4% of CAST-Seq reads indicate deletions and inversions larger than 200 bp, whereas only 0.6% of reads were detected in AAV-HITI scRNA-treated mice (Figure S8B).

Moreover, we used *in silico* prediction to identify putative OTs and selected the top 10 candidates for downstream analysis (Table S6). We PCR-amplified the region surrounding the expected OT cleavage site with primers specific for each locus (Table S7). PCR products were analyzed by NGS for the presence of indel mutations. Indels were found to be almost undetectable or present at similar frequency in AFgRNA- and AFscRNA-treated samples (Figure 6B), underlining that the employed CRISPR-Cas9 nuclease is highly specific. Furthermore, we measured serum albumin levels in AAV-HITI-treated (gRNA or scRNA) MPS VI mice and unaffected littermates at P360 and confirmed that our AAV-HITI approach does not alter endogenous albumin expression (Figure 6C). Histopathological analysis performed on liver sections from all AAV-HITI-treated (AFgRNA or AFscRNA) mice and unaffected controls showed no evidence of hepatocellular carcinoma (HCC) at this stage (P360; Figure 6D).

### Dose response of liver-directed AAV-HITI in newborn mice

We next performed a dose-response study with two additional doses of AAV-HITI: a medium dose (MD) of 3.9 × 10^13^ total GCs/kg and a low dose (LD) of 1.2 × 10^13^ total GCs/kg. AAV-HITI efficiency at these new doses was assessed in newborn C57BL/6 mice using the dsRed HITI donor DNA. Animals were randomly assigned to the MD or LD group and injected systemically at P1–2, as described earlier. The percentage of hepatocytes expressing dsRed as a result of the integration (used to evaluate AAV-HITI efficiency) was quantified on liver cryo-sections 1-month post-treatment and found to be AAV dose dependent (Figure S9A). Next, the efficacy of AAV-HITI at these same doses (MD and LD) was evaluated in newborn MPS VI mice. A similar dose-dependent effect on serum ARSB activity was observed. Mice administered with AAV-HITI at MD exhibited sustained and stable serum ARSB activity levels starting from 1-month post-treatment, while mice treated with AVV-HITI at LD stably achieved ∼50% of the normal ARSB levels (Figure S9B). Urinary (Figure S9C) and tissue GAG levels were normalized in AFgRNA-treated MPS VI mice (Figures S10A–S10D). GAGs storage was significantly decreased also in histological sections from the heart mitral valve and myocardium (Figure S10E) regardless of the dose used. In MD AFgRNA-treated mice, we also observed a significant amelioration in the skull width/length ratio and in the femur length compared to AF controls (Figures S10F–S10H).

### Liver-directed AAV-HITI is effective in adult mice

**Figure 7:**
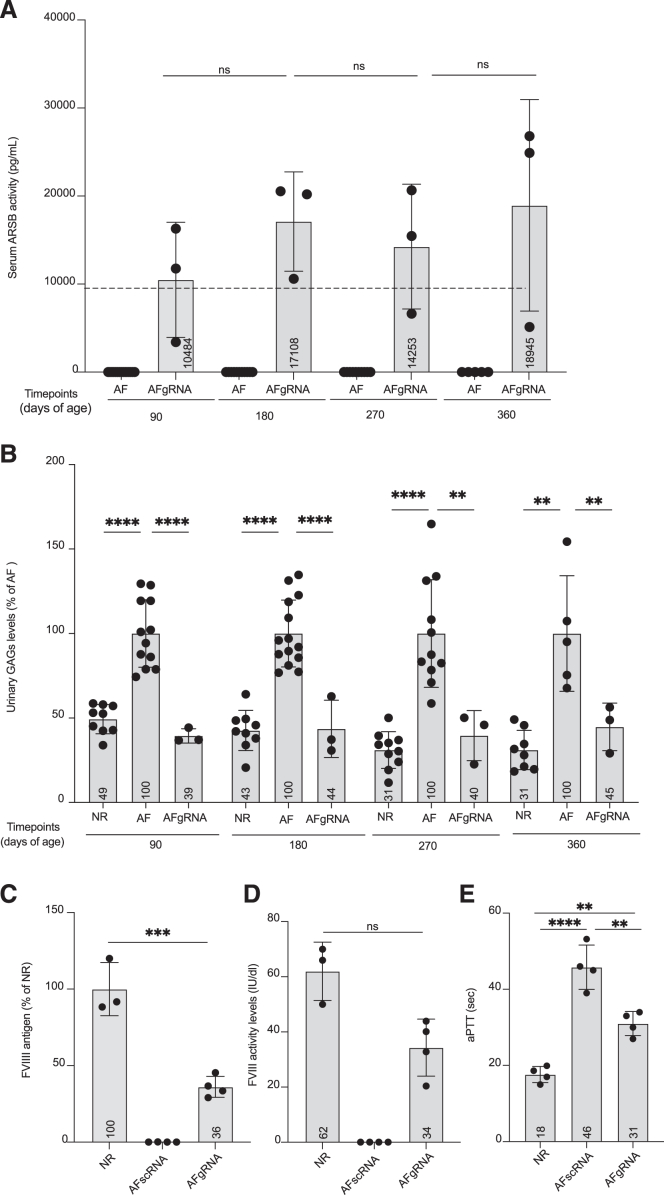
Liver-directed AAV-HITI in adult mice (A) Serum ARSB activity analyzed at different time points in MPS VI mice left untreated (AF, *N* = 14) or treated with AAV-HITI-gRNA (AFgRNA, *N* = 3) at the total dose of 1.2 × 10^13^ total GCs/kg. (B) Urinary GAG levels at different time points in MPS VI mice left untreated (AF, *N* = 14) or treated with AAV-HITI-gRNA (AFgRNA, *N* = 3) reported as a percentage of GAG levels in untreated MPS VI mice. NR, sera samples from normal animals. Statistical differences were assessed at P90 by Brown-Forsythe and Welch ANOVA tests: ∗∗∗∗*p* < 0.0001 between NR and AF; ∗∗∗∗*p* < 0.0001 between AF and AFgRNA; by Kruskal-Wallis test at P180: ∗∗∗∗*p* < 0.0001 between NR and AF; ∗∗∗∗*p* < 0.0001 between AF and AFgRNA; by ordinary one-way ANOVA test at P270: ∗∗∗∗*p* < 0.0001 between NR and AF; ∗∗*p* < 0.0007 between AF and AFgRNA; at P360: ∗∗*p* = 0.0012 between NR and AF; *p* = 0.9999 between NR and AFgRNA; ∗∗*p* = 0.0078 between AF and AFgRNA. The differences in the number of analyzed samples within the same group of treatment were due to sample availability. Each dot corresponds to a single animal within each group at different time points. (A and B) The differences in the number of analyzed samples within the same group of treatment at different time points were due to sample availability. (C) FVIII antigen levels detected in AAV-HITI (AFscRNA, *N* = 4; AFgRNA, *N* = 4)-treated adult HemA mice and normal controls (NR, *N* = 3) 1-month post AAV-HITI treatment. ∗∗∗*p* = 0.0001 between NR and AFgRNA. (D) FVIII activity levels evaluated in HemA mice (AFgRNA, *N* = 4; AFscRNA, *N* = 4) and normal controls (NR, *N* = 3) by chromogenic assay 1-month post AAV-HITI treatment. Statistical differences were assessed by ordinary one-way ANOVA test. *p* value between NR and AFgRNA = 0.0783. (E) Activated partial thromboplastin time (aPTT) measured 1 month post AAV-HITI treatment in HemA AAV-HITI (AFgRNA, *N* = 4 and AFscRNA, *N* = 4) mice and normal controls (NR; *N* = 4). ∗∗∗∗*p* < 0.0001 between NR and AFscRNA; ∗∗*p* = 0.0028 between NR and AFgRNA; ∗∗*p* = 0.0014 between AFscRNA and AFgRNA. All data are represented as mean ± standard deviation. See also Figure S11.

To understand the applicability and efficiency of AAV-HITI in the adult liver, MPS VI and HemA mice were randomly administered systemically at 6 weeks of age an LD of 1.2 × 10^13^ GCs/kg of AAV-HITI vectors. For MPS VI mice detectable serum ARSB activity was measured in the AFgRNA-treated animals at 90 days of age, and this activity became supraphysiological at the subsequent time points (Figure 7A). Moreover, AFgRNA-treated mice showed decreased levels of urinary (Figure 7B) and tissue GAGs compared to the AF control mice (Figures S11A–S11D). GAGs storage was also reduced in histological sections from the heart mitral valve and myocardium (Figure S11E) while no significant improvement was observed in the analyzed bones (Figures S11F–S11H). In HemA mice at 30 days post AAV-HITI delivery, we observed FVIII protein levels around 36% of normal (Figure 7C), therapeutic levels of FVIII activity (Figure 7D), and reduced clotting time (Figure 7E). These data indicate that AAV-HITI is effective in the adult liver at moderate AAV doses in addition to newborn.

## Discussion

In this study, we have demonstrated the therapeutic efficacy of liver-directed AAV-HITI at the highly transcribed m*Alb* locus both in the newborn and in the adult liver. Several previous studies have reported targeting of the albumin locus^30^^,^^37^^,^^55^ with overall similar levels of transgene integration to those we obtained here. HITI, however, hijacks NHEJ able to target both newborn and adult tissues^21^^,^^22^^,^^25^^,^^34^ without the need for long homology arms in the donor template. This allows to accommodate larger therapeutic sequences. Importantly, here we show that the majority of the full-length donor DNA integration happened after CRISPR-Cas9 cleavage of the ITRs, as intended by its design, which is in line with the lower integration rate of the AAV-*Sp*Cas9 vector which lacks the inverted gRNA sites. This, combined with the slightly favored desired integration of the donor DNA that we observed using CAST-Seq, highlights the importance of the inverted gRNA sites at both extremities of the donor DNA. In addition, we found that integration of internal AAV-HITI donor DNA sequences was rare, suggesting that excessive end resection upon CRISPR-Cas-mediated cleavage of the HITI donor DNA does not occur.

Furthermore, we demonstrated the safety of AAV-HITI following neonatal delivery at high doses. AAV-HITI combines two different technologies whose safety is under evaluation: AAV vectors and CRISPR-Cas9 nuclease. AAV vectors are predominantly non-integrative; however, it has been shown that portions of their genome can integrate into pre-existing DSBs at a low frequency.^56^ Despite this low level of integrations, a number of studies have been conducted in mice to investigate potential genotoxicity associated with these events particularly after neonatal treatment.^57^^,^^58^ Data collected in animal studies suggest that several factors such as age of treatments, vector doses, and vector design may influence genotoxic events related to AAV vectors.^57^ However, no confirmed genotoxicity associated with AAV therapies has been described in humans to date.^57^ In our recent publication,^21^ we performed genome-wide analysis of liver genomic DNA following AAV neonatal delivery and found partial AAV integrations at spontaneous DSBs. Here, we showed that portions of the AAV vector genomes can be found at the CRISPR-Cas9-induced DSBs with most of these integrations falling within the AAV ITR sequences, in line with previously reported data.^59^^,^^60^^,^^61^ To further investigate the safety of our approach, we thoroughly assessed AAV-*Sp*Cas9 integration and long-term expression and found that, albeit detectable, full-length integration occurs at low levels and does not result in long-term detectable protein expression in the transduced liver.

Moreover, when nuclease-based strategies are proposed for therapeutic applications, the need to assess nuclease specificity and genome integrity is crucial to reduce the risk of genotoxicity.^54^ Indeed, poor nuclease specificity may result in indel mutations at OT sites and large chromosomal aberrations.^54^ Notably, the CAST-Seq data combined with the assessment of *in silico*-predicted OT sites showed that, despite low rates of partial AAV integrations at the on-target site, neither intra-chromosomal rearrangements nor OT indels could be detected. CAST-Seq further revealed that a minor portion of reads (2.4%) from the target locus pointed at large chromosomal aberrations. Despite their low frequency, these events warrant consideration for potential safety implications. Of note, these data are in stark contrast to the high frequency (often exceeding 50%) at which such reads are found in cells that were exposed to a CRISPR-Cas9 nuclease in the absence of a donor DNA template (Klermund et al., in revision; Rhiel et al., in preparation), suggesting that the presence of an AAV donor DNA template could mitigate these chromosomal aberrations. However, more experiments, and in particular proper side-by-side comparisons, are needed to confirm this hypothesis.

Additionally, the absence of HCC up to 1 year after neonatal treatment supports the safety of the AAV-HITI platform. Of note, our AAV-HITI approach is based on the use of a weak HLP to drive *Sp*Cas9 expression and no promoter in the donor DNA. This design should limit the risk of genotoxicity, as also demonstrated by Chandler et al.^62^ Nonetheless, to exclude potential AAV-HITI-induced genotoxicity associated with HCC development in mice, longer follow-up up to 18–24 months of age will be necessary.^63^

The unaltered secreted albumin levels support the safe design of HITI in albumin intron 13, as correct HITI insertions are not predicted to affect albumin gene expression. However, we should not underestimate the risk that undesired integration events may introduce potential cryptic acceptor splice sites that could alter expression from the endogenous locus. While this does not affect albumin levels in our case, it should not be overlooked, especially if HITI is performed in other genes.

Since high doses of AAVs may lead to adverse events,^64^^,^^65^ we performed a dose-response study in which we found that AAV-HITI administered in neonatal mice at 1.2 × 10^13^ total GCs/kg resulted in ∼50% of the normal levels of serum ARSB activity, which significantly improved several features of the mouse disease phenotype. If directly translatable to humans, this dose is well tolerated. We also tested this same AAV-HITI dose in adult MPS VI and HemA mice and found it to be highly effective thus expanding the potential applications of AAV-HITI to conditions that require intervention in the adult liver.

Overall, our data support the use of the liver-directed AAV-HITI for sustained and stable expression of therapeutic transgenes to treat inborn genetic disorders. However, additional studies are required to further assess its translational potential. For instance, unwanted AAV genome integration at the on-target site could be mitigated using low doses of AAV-HITI,^25^ or by using non-viral systems such as lipid nanoparticles (LNPs) for nuclease delivery which provide transient expression.^66^

Finally, validating gRNA sequences tailored for the human albumin locus and assessing the effectiveness and safety of AAV-HITI in mice with humanized liver could represent a significant step toward advancing this approach for clinical application.

### Limitations of the study

Future implementations of this study could include longer animal follow-up and formal Investigational New Drug (IND)-enabling studies to further support the safety of the AAV-HITI platform and unbiased LR whole-genome sequencing to further strengthen the characterization of HITI-mediated editing events.

## STAR★Methods

### Key resources table

**Table:** 

REAGENT or RESOURCE	SOURCE	IDENTIFIER
**Antibodies**		

2A	Novus Biologicals	Cat#NBP259627H, RRID:AB_3101802
Cas9	Thermo Fisher Scientific	Cat# MA1-201, RRID:AB_2610640
Calnexin	Enzo Life Sciences	Cat#ADI-SPA-860F, RRID:AB_11178981
hARSB polyclonal antibody	Covalab	Custom

**Chemicals, peptides and recombinant proteins**		

SubX	Leica Biosystems	Cat# 3803670E
Alcian blue	Merck	Cat#A5268-25G
Nuclear Fast Red	Merck	Cat#N8002-5G
Aluminum sulfate hydrate 98%	Merck	Cat# 36,845-8

**Critical commercial assays**		

Coatest® SP4 FVIII-kit	Chromogenix, Werfen, Milan	Cat#K824094
Activated partial thromboplastin time (aPTT)	Teco, Bunde, Germany	Coatron M4
Mouse albumin ELISA kit	Abcam	Cat# 108791
VisuLizeFVIII ELISA kit	Affinity Biologicals	Cat# FVIII-AG

**Deposited data**		

BSgenome.Mmusculus.UCSC.mm10 1.4.3	Bioconductor	https://bioconductor.org/packages/release/data/annotation/html/BSgenome.Mmusculus.UCSC.mm10.html
TxDb.Mmusculus.UCSC.mm10.knownGene 3.10.0	Bioconductor	https://bioconductor.org/packages/release/data/annotation/html/TxDb.Mmusculus.UCSC.mm10.knownGene.html
org.Mm.e.g.,.db 3.16.0	Bioconductor	https://bioconductor.org/packages/release/data/annotation/html/org.Mm.eg.db.html

**Experimental models: Organisms/strains**		

C57BL/6 mice	Envigo	Cat#057
B6; 129S F8tm1Kaz/J	Jackson Laboratory	Cat#004424
MPS VI mice	Institute of Molecular Medicine and Cell Research, University of Freiburg, Germany	N/A

**Software and algorithms**		

Fiji	ImageJ	http://rsbweb.nih.gov/ij/
GraphPad	Prism GraphPad	https://www.graphpad.com/features
Benchling	Benchling	www.benchling.com
Zen Blue	ZEISS ZEN	https://www.zeiss.com/microscopy/en/products/software/zeiss-zen-lite.html
Qpath	Qpath	https://qupath.github.io/
Biorender	Biorender	https://www.biorender.com/
CRISPResso2	CRISPResso2	http://crispresso2.pinellolab.org/submission
CRISPOR web tool	CRISPOR web tool	CRISPOR.org
MinKNOW version 23.04.6	Oxford Nanopore Technologies	https://nanoporetech.com
Minimap2 v. 2.24-r1122	minimap2	https://github.com/lh3/minimap2/releases
BBmap	SOURGEFORGE	https://sourceforge.net/projects/bbmap/
BWA MEM v0.7.17	SOURGEFORGE	https://bio-bwa.sourceforge.net/bwa.shtml
GNU parallel 20220922	GNU Operating System	https://www.gnu.org/software/parallel/
Samtools 1.16.1	Samtools	http://www.htslib.org/
Pcregrep 8.45 2021-06-15		https://man7.org/linux/man-pages/man1/pcregrep.1.html
BBDuk	BBTools	https://jgi.doe.gov/data-and-tools/software-tools/bbtools/bb-tools-user-guide/bbduk-guide/
bedtools v2.27.1	Bedtools website	https://bedtools.readthedocs.io
Biostrings 2.46.0	Bioconductor	https://bioconductor.org/packages/release/bioc/html/Biostrings.html
ChIPseeker 1.14.2	Bioconductor	https://bioconductor.org/packages/release/bioc/html/ChIPseeker.html
CAST-Seq	CAST-Seq github	https://github.com/AG-Boerries/CAST-Seq
R 4.2.2	CRAN	https://www.r-project.org

**Other**		

TaqMan-PCR	Applied Biosystems	Cat# 4304437
O.C.T. matrix	Kaltek	Cat# 0782
Vectashield with DAPI	Vector Laboratories	Cat# H-1200-10
Trisodium citrate 0.109 M	BD	Cat# 5T31.363048
Bradford Reagent	Bio-Rad	Cat#5000006
Protease inhibitor cocktail	Merck	Cat#78430
Ponceau	Merck	Cat#P7170-1L
KAPA HiFi Hotstart Polymerase	Roche	Cat#07958927001
Ampure XP beads	Beckman Coulter	Cat# A63881
DNeasy Blood & Tissue kit	Qiagen	Cat# 69504
QIAquick® Gel Extraction Kit	Qiagen	Cat# 28706
Ligation Sequencing Kit V14	Oxford Nanopore Technologies	Cat# SQK-LSK114
LightCycler 480 SYBR Green I Master	Roche	Cat#04707516001

### Resource availability

#### Lead contact

Further information and requests for resources and reagents as well as datasets and protocols should be directed to and will be fulfilled by the lead contact, Alberto Auricchio (auricchio@tigem.it).

#### Materials availability

This study did not generate new unique reagents.

#### Data and code availability

• All data reported in this paper will be shared by the lead contact upon request.
• This study does not report original code.
• Any additional information required to reanalyze the data reported in this work paper is available from the lead contact upon request.

### Experimental model and subject details

#### Animal models

Mice were housed at the TIGEM animal house (Pozzuoli, Italy) and maintained under a 12 h light/dark cycle at 23 ± 1°C and humidity of 50% ± 5% with food and water available *ad libitum*. Animals were raised in accordance with the Institutional Animal Care and Use Committee guidelines for the care and use of animals in research. C57BL/6J mice were purchased from Envigo Italy SRL (Udine, Italy). The hemophilic A (HemA) mouse model (Cat# B6; 129S-F8tm1Kaz/J) was imported from the Jackson Laboratory (JAX stock). The MPS VI mice were kindly provided by Prof. C. Peters (Institute of Molecular Medicine and Cell Research, University of Freiburg, Germany). HemA mice were maintained by crossing knockout homozygous females with knockout hemizygous males to produce knockout experimental mice. MPS VI mice were maintained as heterozygotes and crossed to produce homozygous knockout experimental mice.

### Methods details

#### Study design

This study was designed to evaluate the efficacy and safety of the AAV vector mediated HITI approach in the liver. Editing efficiency in the liver was defined in cryo-section fluorescent images by using the ImageJ software to count and calculate the percentage of hepatocyte positive for the *Discosoma* sp*.*-Red (dsRed) fluorescent protein. In all *in vivo* studies, mice were randomly assigned to each treatment group. Therapeutic efficacy in the liver was assessed by evaluating the impact of neonatal systemic delivery of AAV-HITI on the phenotype of two different animal models of Mucopolysaccharidosis type VI (MPS VI) and Hemophilia A (HemA) while for safety studies only MPS VI mice were used. In addition, in the studies involving the MPS VI disease model, female and male mice were considered equivalent and randomly assigned to treatment groups whereas in the study involving hemophilic animals only males were used. Littermate controls were used when available. In both cases, observers were blind to both genotype and treatment of the animals. Sample sizes were determined based on previous experience and technical feasibility. Any difference in the numbers of analyzed samples within the same group of treatment at different timepoints was due to sample availability.

#### Systemic vector administration

Studies in animals were carried out in accordance with the Italian Ministry of Health regulation for animal procedures (Ministry of Health authorization number: 352/2020-PR and 626/2022-PR). The injections were performed under general anesthesia, as previously described.^21^ Temporal vein injections in neonatal mice (C57BL/6J or MPS VI, or HemA) were performed at post-natal p1-2 following the protocol published by Gombash Lampe et *al*..^67^ For adult treatments, (C57BL/6J, MPS VI or HemA) retro-orbital injections were performed. The following doses were used in the experiments aimed at evaluating HITI efficiency in C57BL/6J and MPSVI newborn mice: high doses 1.2 × 10^14^ total genome copies (GC/Kg; 6 × 10^13^ GC/kg for each vector); medium doses 3.9 × 10^13^ total GC/Kg (1.95 × 10^13^ GC/kg for each vector); low doses 1.2 × 10^13^ total GC/Kg (6 × 10^12^ GC/kg for each vector). For rescue experiments in neonatal HemA mice, we used 3.9 × 10^13^ total GC/Kg (1.95 × 10^13^ GC/kg for each vector). For rescue experiments in MPS VI and Hema adult mice, we used 1.2 × 10^13^ total GC/Kg (6 × 10^12^ GC/kg for each vector). AAV8 vectors were used for all *in vivo* studies because of their high liver transduction efficiency.^68^

#### Generation of the AAV vector plasmids

The plasmids used for AAV vector production were derived from either the pAAV2.1^21^ or the pTIGEM^69^ plasmids both containing the inverted terminal repeats of AAV serotype 2.^70^ The mouse albumin (m*Alb*) gRNA (5′-GTATTTAATAGGCAGCAGTG-3′) was selected using the benchling gRNA design tool (www.benchling.com), targeting the intron 13 of the albumin locus considering the best predicted on-target and off-target scores. The scramble RNA was designed so as not to align with any sequences in the mouse genome.

#### AAV vector production and characterization

AAV serotype 8 vectors (AAV8) were produced by InnovaVector s.r.l by triple transfection of HEK293 cells followed by two rounds of CsCl_2_ purification.^71^ For each viral preparation, vector titers (genome copies/ml) were determined by averaging the titer achieved by dot-blot and by TaqMan-PCR (Cat#4304437, Applied Biosystems, Carlsbad, California, USA) quantification analysis.^71^

#### Liver fluorescence imaging

To evaluate dsRed expression in liver, C57BL/6J mice were injected at post-natal day (P) 1–2. Livers were harvested at p30 under anesthesia as previously described.^21^ A small piece of each lobe was dissected, fixed in 4% PFA overnight, infiltrated with 15% sucrose over the course of a day and 30% sucrose overnight before being included in O.C.T. matrix (Cat# 0782, Kaltek) for cryo-sectioning. Five-μm-thick liver cryo-sections were cut, distributed on slides, and mounted with Vectashield with DAPI (Cat#H-1200-10, Vector Lab). Cryo-sections were analyzed under a confocal LSM-700 microscope (Carl Zeiss), using appropriate excitation and detection settings for dsRed and DAPI. For assessment of HITI efficiency in mouse liver cryo-sections, three to four images of each liver were acquired at 20× magnification and then analyzed using ImageJ (Fiji) software (http://rsbweb.nih.gov/ij/) as previously described.^21^ We counted a minimum of 900 hepatocytes, identified by DAPI staining of the nucleus, for each image. The hepatocytes expressing the dsRed as result of the integration were unequivocally identified and counted based on their shape. The final value (∼15%) was then obtained by dividing the number of dsRed positive hepatocytes over the total number of DAPI cells and multiplied by 100 to obtain the percentage (%).

#### *In situ* hybridization

Liver cryo-sections were used for the *in situ* hybridization (ISH) with the technology of Basescope (Advanced Cell Diagnostic, USA) in accordance with the manufacturer’s protocol.^72^ ISH labeling of HITI donor DNA was performed using a 3-ZZ paired probe (sense) (Cat# 1134401, ACD). ISH was performed using the BaseScope Duplex Reagent Kit (Cat# 323871, ACD). Custom pre-treatment conditions included target retrieval of 30 min at 95 C–100 C, RNAscope Protease III (Cat # 322380, ACD) for 30 min at room temperature (RT). Custom counterstaining with Mayer haematoxylin for 1 min at RT was performed after probe, amplification, and chromogen steps in the kit assay. Sections were analyzed with scanned with ZEISS Axio Scan.Z1. The whole digital slides were viewed by zen blue software. Red positive spots of HITI DNA were quantified with QuPath software. For the quantitative analysis of positive signals, we selected 4–6 liver regions at 20× magnification from mice belonging to AAV-HITIgRNA, -scRNA and PBS experimental groups, and the results were expressed as a percentage of positive nuclei over the total nuclei analyzed.

#### Serum ARSB enzymatic activity

Blood samples were collected at different timepoints from AAV-HITI-treated and control MPS VI mice as previously reported.^21^ Serum ARSB activity was measured by an immune capture assay based on the use of a specific custom-made anti-hARSB polyclonal antibody (Covalab, Villeurbanne, France) following a previously described protocol.^21^

#### Chromogenic assay

Blood sampling was performed by retro-orbital withdrawal and nine parts of blood were collected into one part of buffered trisodium citrate 0.109 M (Cat#5T31.363048; BD, Franklin Lakes, NJ, USA). Plasma was collected after centrifugation at 3.000 rpm at 4°C for 15 min. Chromogenic assay was performed on plasma samples using the Coatest SP4 FVIII-kit (Cat# K824094; Chromogenix, Werfen, Milan, Italy) according to the manufacturer’s instructions. Results are expressed as International Units (IU) per decilitre (dL).

#### FVIII antigen detection

To quantify the levels of FVIII antigen, an ELISA kit (Cat#FVIII-AG; VisuLize FVIII ELISA kit, Affinity Biologicals, Arcore, Italy) was used according to the manufacturer’s instructions.

#### GAG level analysis in urine and tissues

Urine samples were collected over 24 h using metabolic cages at p60, p90, p180, p270 and p360 from MPS VI-treated and control mice. Samples were centrifuged briefly to remove debris and diluted 1:50 in water to measure GAGs content. Fifty μL of diluted urine or 250 μg of protein lysate were then used for GAGs evaluation as previously reported.^21^

#### Alcian blue staining in histological sections

After methacarn fixation, livers, kidneys, spleens, and hearts were dehydrated by immersion in increasing concentrations of alcohol (70%, 80%, 90%, 100%) and then in Sub-X. All tissues were embedded in paraffin and sectioned transversally into 7-μm-thick serial sections on a microtome. Tissue sections were de-paraffinized, rehydrated, then washed in water and stained with 1% Alcian blue (Cat#A5268-25G, Merck) in hydrochloric acid (5 min for hearts, 60 min all remaining tissues). Counter-staining was performed with 0.1% Fast-Red (Cat#N8002-5G, Merck) in water for 1 min. Liver, kidney, and spleen sections stained with Alcian blue were imaged with Leica Microscope DM5500. Heart sections stained with Alcian blue were scanned with ZEISS Axio Scan.Z1. The whole digital slides were viewed by Zen Blue software (https://www.zeiss.com/microscopy/en/products/software/zeiss-zen-lite.html). Quantitative analyses of Alcian blue staining in myocardial tissue and in mitral valves were performed by Qpath software (https://qupath.github.io/). Alcian blue quantification in myocardium was measured in two fields of identical area. Alcian blue quantification in the mitral valve was performed on the entirety of the valve. Results are expressed as Alcian blue positive area/total area.

#### Bone analysis

Radiography images were performed on AAV-HITI-treated MPS VI mice and unaffected controls were anesthetized with an intraperitoneal injection of ketamine (10 mg/Kg) combined with medetomidine (1 mg/Kg). Skull weight and length, and tibia and femur length, were measured using ImageJ (Fiji) software (http://rsbweb.nih.gov/ij/).

#### Tail-clip assay

Mice were anesthetized and the distal part of the tail was cut at 2–3 mm of diameter and immediately put in a prewarmed 0.9% saline solution and allowed to bleed for 10 min without disturbance and the tails were then cauterized. The mixture of collected blood and physiological saline solution was centrifuged at 1500 g for 5 min and the total volume of blood lost was measured.

#### Activated partial thromboplastin time (aPTT) assay

Activated partial thromboplastin time (aPTT) was measured on plasma samples with Coatron M4 (Teco, Bünde, Germany) using the aPTT program following the manufacturer’s manual.

#### Western blot analysis

Protein concentration in AAV-HITI-treated sera samples collected from MPS VI and HemA mice was determined by Bradford Reagent (Cat#5000006, Bio-Rad). Ten microgram of sera were denatured at 100°C for 5 min in 1× Laemmli sample buffer supplemented with 1 M dithiothreitol (DTT). Next, protein samples were resolved on a 4–15% SDS–PAGE and transferred onto polyvinylidene difluoride (PVDF) membrane. After transfer to PVDF membrane, blot was blocked with TBS-Tween-20 containing 5% non-fat milk for 1 h at room temperature followed by incubation with primary antibody (Cat#NBP259627H,Novus Biologicals; dilution: 1/2,000) 1h at 4°C. Ponceau (Cat#P7170-1L, Merck) was used as normalizer in sera blot. Liver specimens were mechanically homogenized using metal beads and lysed in RIPA buffer, supplemented with protease inhibitor cocktail (Cat#78430, Merck). Samples were incubated for 30 min on ice, vortexed every 10 min, and centrifuged at 16,200 *g* for 20 min. Pellets were discarded and lysates were used for Western blot analyses. After lysis, protein samples were treated as mentioned above. Hundred micrograms of liver proteins were loaded for each specimen into a 6% SDS–PAGE; after transfer to PVDF membrane, blot was blocked with TBS-Tween-20 containing 5% non-fat milk for 1 h at room temperature followed by incubation with primary antibody overnight at 4°C. The primary antibodies used for immuno-blotting were: rabbit anti-*Sp*Cas9 (Cat# MA1-201, Thermo Fisher Scientific; dilution: 1/1,000) mouse anti-Calnexin (Cat#ADI-SPA-860F, Enzo Life Sciences; dilution: 1/2,000).

#### Serum albumin measurement

Blood was collected at p360 from AAV-HITI-treated and control mice via eye bleeding and centrifuged as previously described.^21^ Serum samples were diluted 1:30.000 and analyzed with a mouse albumin ELISA kit (Cat#108791, Abcam, Cambridge, UK) following the manufacturer’s instructions.

#### Histopathological analysis

Right, left, median, and caudal liver lobes were embedded in paraffin and sectioned at a thickness of 5 μm. To assess histological features, Haematoxylin/Eosin (Diapath) staining was performed according to standard protocols and samples were analyzed by an expert histopathologist from Histopathology Unit (Cogentech Ltd. Benefit Corporation, Milan) in blind.

#### DNA extraction

DNA extraction was performed using the DNeasy Blood & Tissue kit (Cat# 69504, QIAGEN) following the manufacturer’s instructions.

#### Quantitative PCR (qPCR)

Viral genome copies (GC) were measured by qPCR analysis in 100ng of genomic DNA extracted from AAV-HITI-treated liver samples at 1-week, -month and 1-year upon AAV-HITI administration, using the LightCycler 480 SYBR Green I Master mix (Cat#04707516001, Roche). The following primers: forward HLP: 5′-CTCCTCCGATAACTGGGGTGAC-3′ and reverse HLP: 5′-GCCCTGTCCTCGTCCGTATTTA-3′, were used to detect AAV8 -*Sp*Cas9 vector; forward BGH 5′-TCTAGTTGCCAGCCATCTGTTGT-3’; reverse BGH 5′-TGGGAGTGGCACCTTCCA-3′ were used to detect the AAV-HITI donor DNA following the qPCR protocol from Roche. Briefly, enzyme activation was set at 95°C during 20 s −3 min (1 cycle), followed by the denaturation step at 95°C during 3 s and annealing extension and acquisition at 60°C at 20 s with the last two points set at 40 cycles.

#### CAST-Seq

To identify chromosomal aberrations, nominate off-target sites and analyze integration events at the on-target site, we performed CAST-Seq.^54^ High-throughput sequencing libraries were essentially prepared according to Turchiano et al., 2021.^54^ In search for off-targets, a third decoy primer was included to prevent predominant amplification of the target locus after successful HITI. In total, three CAST-Seq libraries from liver genomic DNA samples extracted from three different 1 year-old MPS VI mice treated as newborn with high doses of AAV-HITI were prepared and compared to a single library generated from control mice samples (scRNA). In order to analyze in depth the integration of AAVs and parts thereof, two CAST-Seq libraries of HITI-treated and a single library of a control mouse were sequenced. For these samples, the HITI-specific decoy primer was not used during library preparation. Sequencing of the samples was outsourced to GeneWiz (division of Azenta Life Sciences) who collected 2x150bp paired-end reads using an Illumina NovaSeq 6000 instrument. The bioinformatics analysis was performed using the previously published pipelines.^54^^,^^73^

#### Nanopore long-read sequencing

Fragments for long-read sequencing were amplified from DNA extracted from 1 year-old MPS VI mice treated as newborn with high doses of AAV-HITI using KAPA HiFi Hotstart Polymerase (Cat#07958927001, Roche). The cycling conditions were: 3 min at 98°C followed by 35 cycles of 98°C for 20 s, 61°C (5′ junction) or 63°C (3′ junction) for 30 s, and 4 min at 72°C. A final elongation of 10 min at 72°C was programmed after the last cycle. Multiple PCR reactions were pooled and the pool subjected to bead purification using 0,9X Ampure XP beads (Cat# A63881, Beckman Coulter) and fragments eluted in a small volume of water (30 μL). The thus concentrated PCR pool was subjected to agarose gel electrophoresis and the prominent PCR product was gel-extracted using the QIAquick Gel Extraction Kit (Cat# 28706, Qiagen). With the PCR step, 24-nt long barcodes were introduced, in accordance with previously published data.^74^ The 5′ and 3′ junction PCR products were in a next step prepared for sequencing using the Ligation Sequencing Kit V14 (Cat# SQK-LSK114, Oxford Nanopore Technologies) following the manufacturer’s instructions. Samples were immediately sequenced on an R10.4.1 MinION Flow Cell (Cat#FLO-MIN114, Oxford Nanopore Technologies) using a MinION sequencing device (Cat#MIN-101B, Oxford Nanopore Technologies) operated with MinKNOW software version 23.04.6. Reads were demultiplexed by employing a custom pipeline that allows for up to 4 mismatches in the barcode sequence using pcregrep and GNU Parallel.^75^ We decided to allow up to 4 mismatches based on previously published data^76^ as a compromise between a low false annotation rate versus loss of valuable information. Of note, the two samples described here were analyzed together during one sequencing run in the absence of other samples thus reducing the chance of wrongful read assignment. Obtained reads were aligned using Minimap2 v. 2.24-r1122,^77^ and further processed with Samtools.^78^ ITR sequence insertions were counted from the CIGAR string in a window of 200 bp around the integration site junctions using a custom script.

#### Short-read sequencing

Assessment of Indels in short sequencing reads was performed using CRISPResso2.^79^ For the quantification of ITR sequence integration, raw FASTQ files were processed using BBduk (https://sourceforge.net/projects/bbmap/) and aligned to the reference amplicon using BWA MEM v0.7.17.^80^ Correctly mapped reads were extracted from the FASTQ file and aligned to the ITR sequence. The total number of all alignments and the number of all alignments to the ITR sequence were quantified and the relative frequency was calculated.

#### Off-target site analysis

The top 10 predicted off-target sites were identified using the CRISPOR web tool (CRISPOR.org)^81^ based on GRCm39/mm39 mouse genome reference, and sorted by CFD off-target scores. Equal amounts of genomic DNA extracted from 3 AAV-HITI-gRNA-treated livers and 3-scRNA-treated livers were used to amplify between 150 and 300 bp genomic regions flanking the off-targets. PCR products were amplified with specific primers (Table S7) and quantified using Qubit 4.0 fluorometric Assay (Thermo Fisher Scientific). An equal amount of each PCR product from each liver sample was pooled together. Next-generation sequencing (NGS) amplicon library were preprepared from 10 ng of pooled PCR products using the NEGEDIA DNAseq Low Input sequencing service (NEGEDIA s.r.l) which included library preparation, quality assessment and sequencing on a NovaSeq 6000 sequencing system using a paired-end, 2x150 cycle strategy (Illumina Inc.). The resulting FASTQ files were then analyzed using CRISPRessoV2,^79^ using the off-targets sequences as reference for analysis. A 17-nucleotide window (upstream and downstream of the cleavage site) was considered for evaluation. NGS analysis was performed by NEGEDIA s.r.l.

### Quantification and statistical analysis

For all the statistical analysis, we performed the Shapiro-Wilk test to check that each condition had followed the normal distribution (null hypothesis). To assess significant differences between two conditions the non-parametric Mann-Whitney test was used in the case of rejection of the null hypothesis (*p*-value <0.05). If the null hypothesis was not rejected (*p*-value ≥0.05) the parametric unpaired T-test was applied and the F-test was used to check the homoscedasticity between the two compared conditions. In case of rejection of the null hypothesis (*p*-value <0.05) the parametric Welch’s t-test was applied. For multiple comparisons and post hoc tests, non-parametric Kruskal-Wallis test was used in the case of rejection of the null hypothesis (*p*-value <0.05). The parametric one-way ANOVA test was applied in case of not rejection of the null hypothesis (*p*-value ≥0.05). In this case, we also performed the Brown-Forsythe test to check the homoscedasticity between the compared conditions (null hypothesis): we used the parametric Welch’s one-way ANOVA in case of rejection of the null hypothesis (*p*-value <0.05). For completeness, we computed the *p*-values with post hoc tests for the pairwise multiple comparisons: Tukey’s test for one-way ANOVA, Dunnet’s test for Welch’s one-way ANOVA, and Dunn’s test for Kruskal-Wallis test. *p*-values are represented as follows: ∗ *p*-value <0.05; ∗∗ *p*-value <0.01; ∗∗∗ *p*-value <0.001; ∗∗∗∗ *p*-value <0.0001.

## Supplemental information

Document S1. Figures S1–S11 and Tables S2–S7

Table S1. AAV-HITI vectors’ sequences, related to Figures 2 and 4C

Document S2. Article plus supplemental information
