# Supplementary material for: Safe and effective liver-directed AAV-mediated homology-independent targeted integration in mouse models of inherited diseases
Source: Cell Rep Med. 2024 Jun 18;5(7):101619. doi: 10.1016/j.xcrm.2024.101619 (PMC11293346; doi:10.1016/j.xcrm.2024.101619)
Supplement: Document S1. Figures S1–S11 and Tables S2–S7 [file mmc1.pdf]

Cell Reports Medicine, Volume *5*

Supplemental information

Safe and effective liver-directed AAV-mediated homology-independent targeted integration in mouse models of inherited diseases

Federica Esposito, Fabio Dell'Aquila, Manuel Rhiel, Stefano Auricchio, Kay Ole Chmielewski, Geoffroy Andrieux, Rita Ferla, Paula Sureda Horrach, Arjun Padmanabhan, Roberto Di Cunto, Simone Notaro, Manel Llado Santeularia, Melanie Boerries, Margherita Dell'Anno, Edoardo Nusco, Agnese Padula, Sofia Nutarelli, Tatjana I. Cornu, Nicolina Cristina Sorrentino, Pasquale Piccolo, Ivana Trapani, Toni Cathomen, and Alberto Auricchio

Figure S1

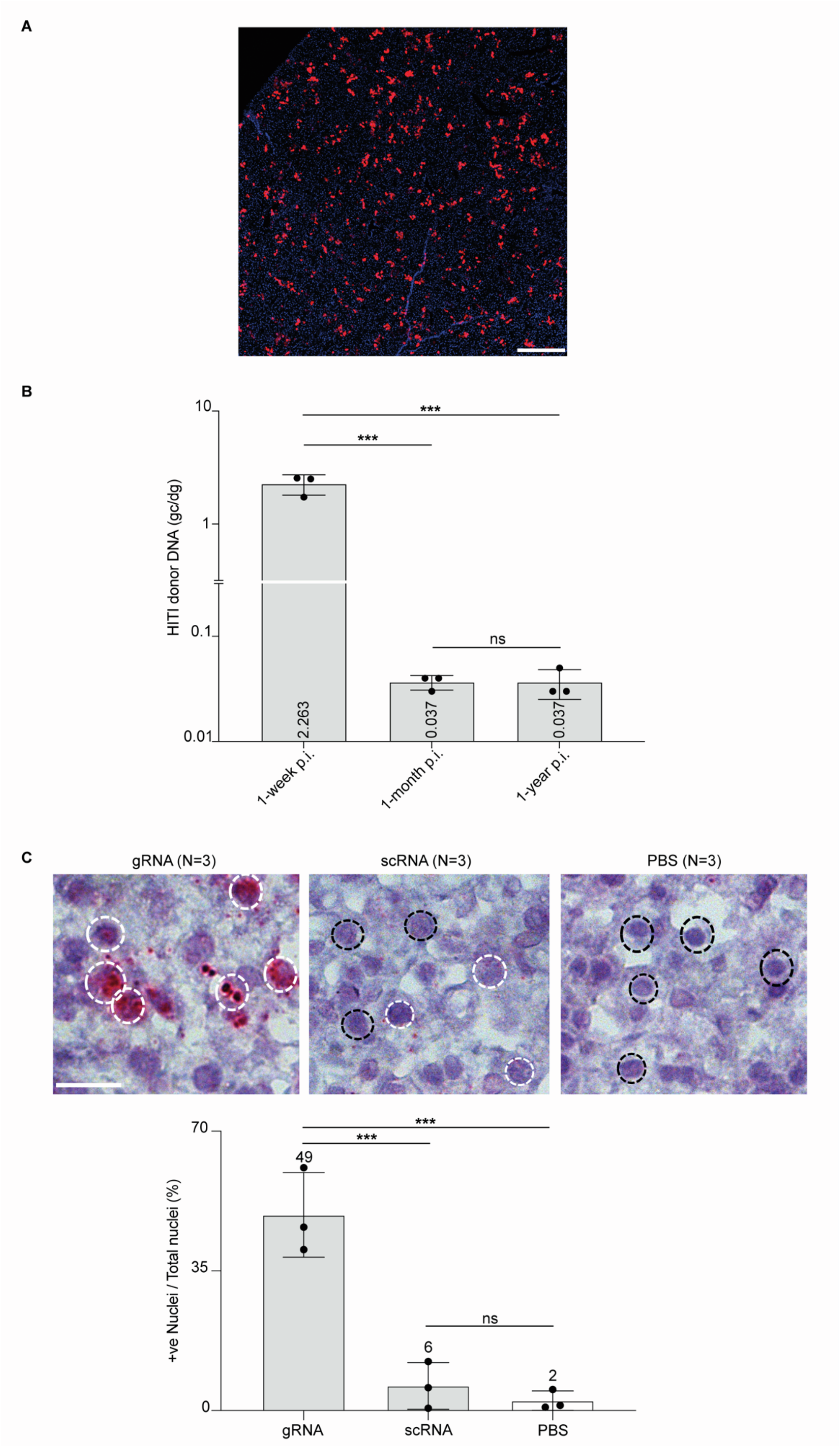

A
B
10
1
0.1
0.01
HITI donor DNA (gc/dg)
***
***
ns
2.263
0.037
0.037
1-week p.i.
1-month p.i.
1-year p.i.
C
gRNA (N=3)
scRNA (N=3)
PBS (N=3)
70
35
0
+ve Nuclei / Total nuclei (%)
***
***
ns
49
6
2
gRNA
scRNA
PBS

Figure S1. AAV-HITI Ds-Red. Related to Figure 2. A) Representative fluorescence microscopy low magnification image of an OCT liver cryo-section from a wild-type mouse injected with AAV-HITI (gRNA) at the total dose of 1.2x10^14 total GC/kg. Scale bar 500 µm. **B**) Real-Time PCR analysis of HITI Donor vector genome in liver of AAV-HITIgRNA-treated newborn mice at different timepoints. Data are represented as mean ± standard deviation of HITI donor DNA genome copies/diploid genomes (gc/dg). For each timepoint N=3 samples were analyzed. Statistical differences were assessed by Ordinary one-way ANOVA Test followed by Tukey's multiple comparisons test. P-value ***= 0.0001 between 1-week and 1-month; p-value ***= 0.0001 between 1-week and 1-year; p-value >0.999 between 1-month and 1-year. **C**) In situ Hybridization (ISH) of HITI donor DNA on liver sections. Upper panel: representative images of ISH in liver sections from (N=3) gRNA, (N=3) scRNA and (N=3) PBS experimental groups; Nuclei are stained in blue. White circles: positive nuclei; Black circles: negative nuclei; Scale bar of all images represent 25µm. Lower panel: quantification of positive nuclei over the total counted nuclei (+ve Nuclei/Total nuclei). Statistical differences were assessed by Ordinary one-way ANOVA Test followed by Tukey's multiple comparisons test. P-value *** = 0.0008 between gRNA and scRNA samples; p-value ***= 0.0005 between gRNA and PBS samples. Data are represented as mean ± standard deviation.

Figure S2

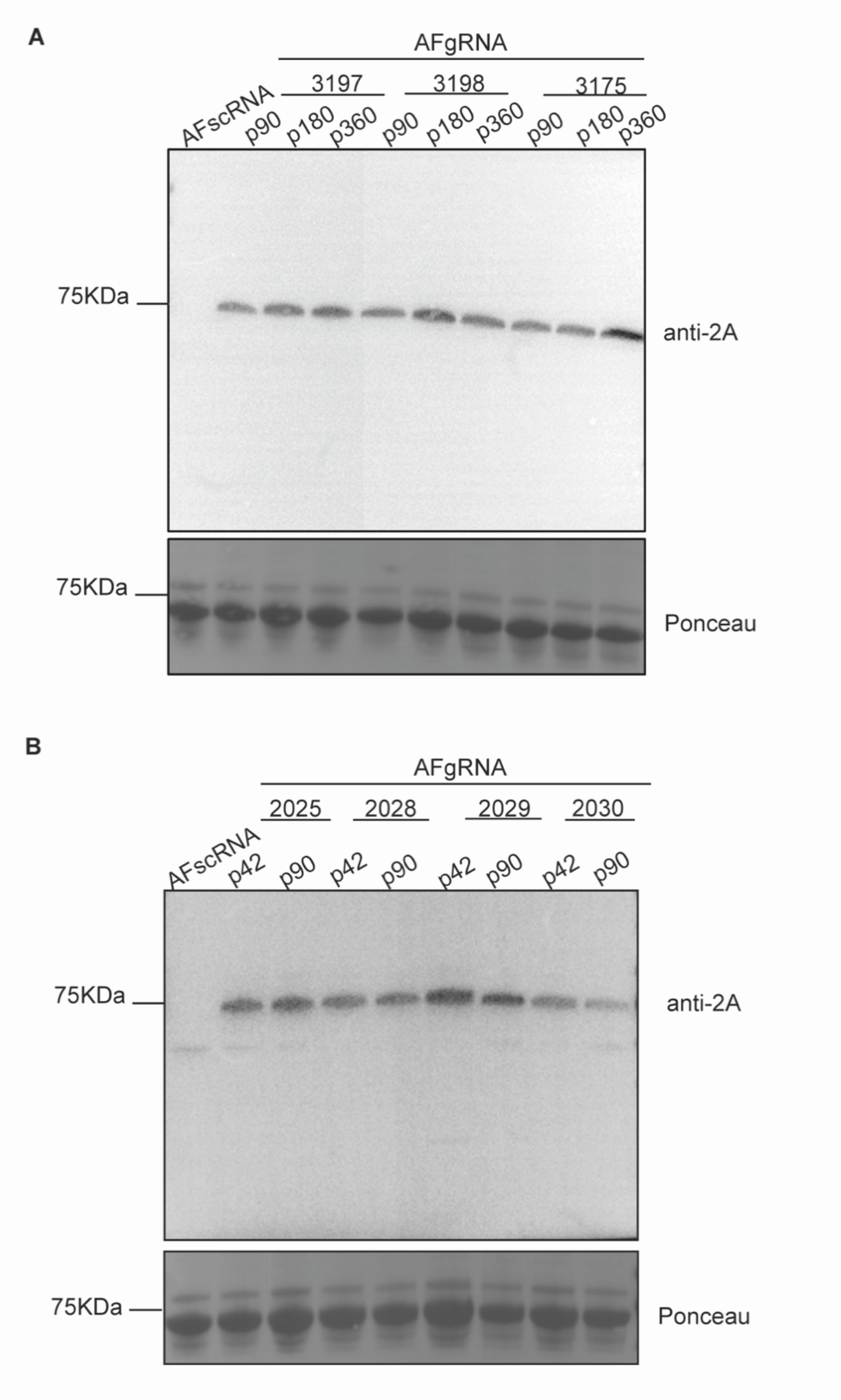

Figure S2. Detection of the modified albumin 2A (Alb-2A) in sera from treated animals. **Related to Figure 2**. **A**) Western blot analysis performed on 10 µg of proteins extracted from sera samples collected from AAV-HITI-treated MPS VI or HemA (**B**) mice to detect the modified Alb-2A (AFgRNA MPS VI N=3: 3197,3198,3175; AFgRNA HemA N=4: 2025,2028,2029,2030). In **A**) 3 different timepoints were analyzed: p90, p180 and p180 days post AAV-HITI neonatal delivery. In **B**) 2 different timepoints were analyzed: p42 and p90. An affected AAV-HITI scRNA-treated mouse (AFscRNA; MPS VI in **A** or HemA in **B**) was used as negative control.

Figure S3

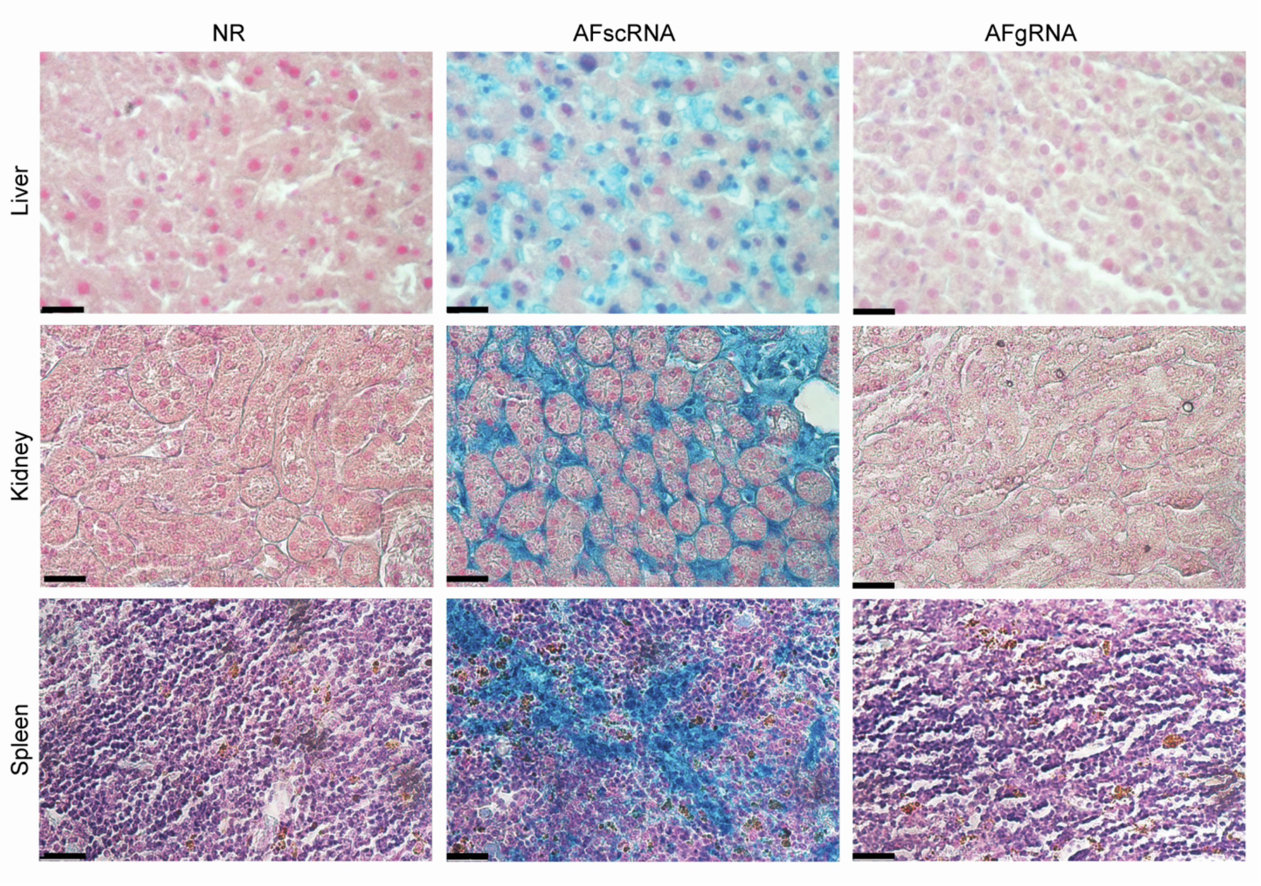

Figure S3. GAGs storage in different AAV-HITI-treated tissues. Related to Figure 3. Alcian blue staining of histological sections from different tissues: liver, kidney, and spleen. Scale bar 50μm. Unaffected untreated mice (NR, N=4); affected MPS VI mice treated with AAV-HITI-scRNA (AFscRNA, N=8); affected MPS VI mice treated with AAV-HITI-gRNA (AFgRNA, N=8).

Figure S4

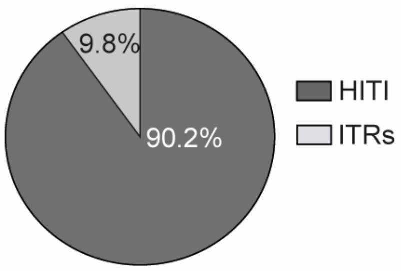

Figure S4. Short reads sequencing of 5’ HITI junction from liver genomic DNA. Related to Figure 5. Pie chart showing the percentage (%) of short reads in which donor DNA integration was HITI-mediated or ITRs-mediated (ITRs) at the 5’ junction site.

Figure S5

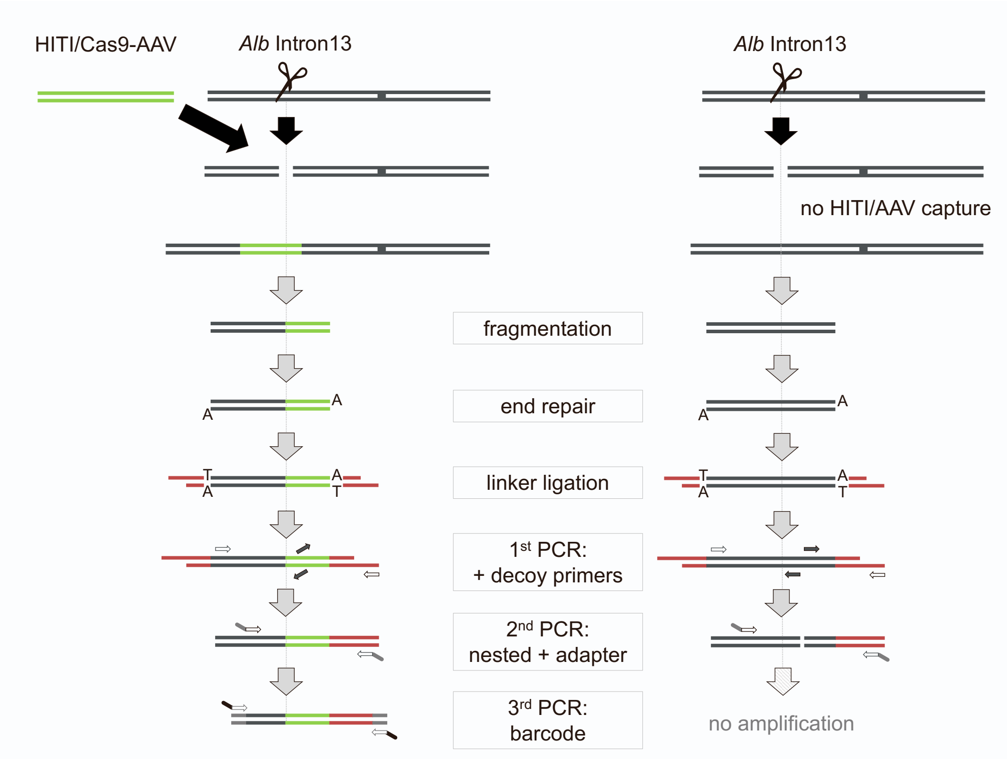

Figure S5. Schematic representation of CAST-Seq at the on-target site. Related to STAR Methods. The cartoon shows the CAST-Seq library preparation for e detection of integrated AAV-HITI vectors at the on-target site upon nuclease cleavage. Liver genomic DNA is fragmented randomly, and end repaired to add a 3′-A overhang which is used for ligation of a short linker (red). The first PCR is performed with bait and prey primers (white arrows) binding to the target site and the linker, along with decoy primers (black arrows) binding to the on-target locus near the cleavage site. The second PCR with nested primers adds adapters that are used in the third PCR to add barcodes.

Figure S6

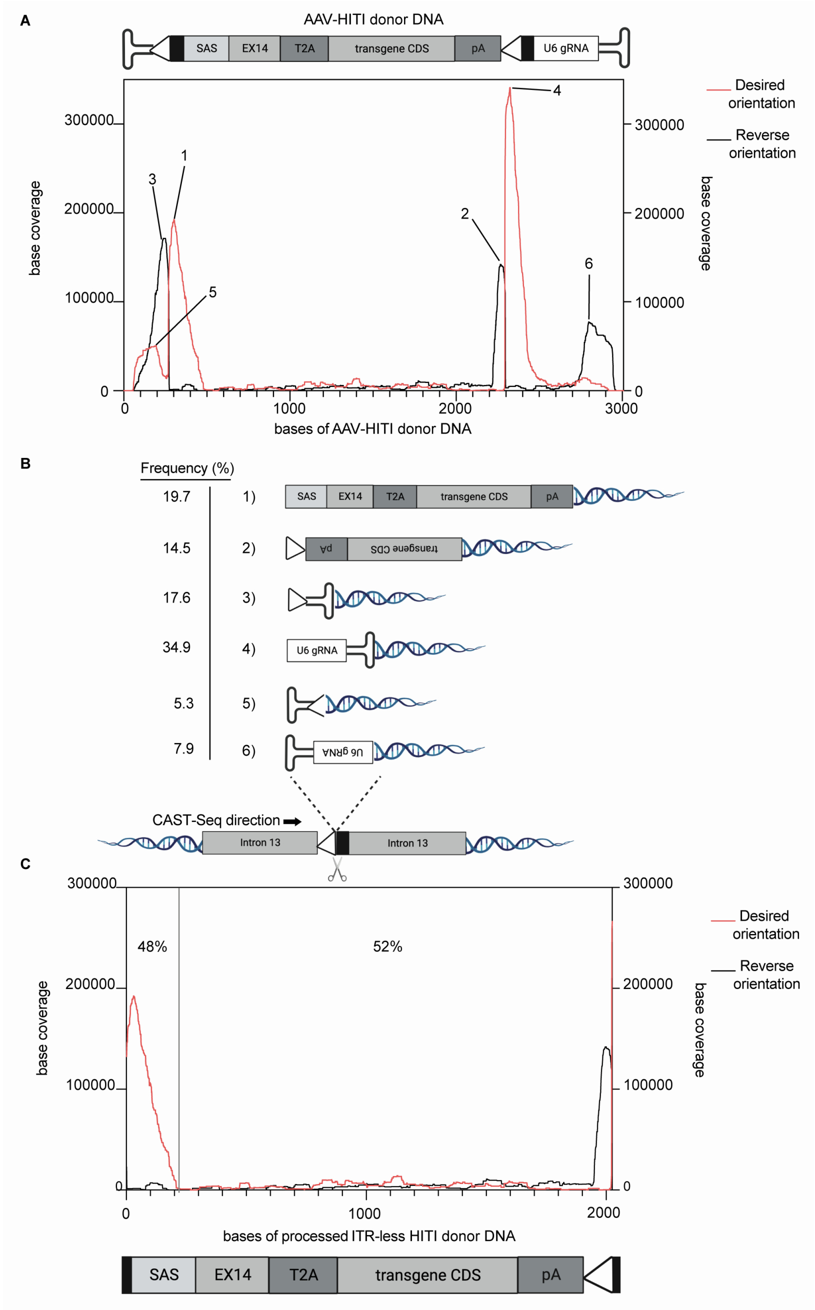

Figure S6. Assessment of HITI integration events at the on-target site by CAST-Seq. Related to Figure 5. A) Single base coverage of integration events by CAST-Seq. For each base, read coverage of the desired (forward strand) and reverse (reverse strand) integration is shown. Labelled are six distinct peaks that represent different integration events. **B**) Schematic representation of integration events. Shown are the six integration events corresponding to the peaks in (**A**), and their relative frequency determined by peak height. **C**) CAST-Seq based single base read coverage of cleaved transgene integration events. For each base, the read coverage of the desired integration and reverse integration is shown. The percentage of functional (left) versus non-productive integration (right) events are indicated.

Figure S7

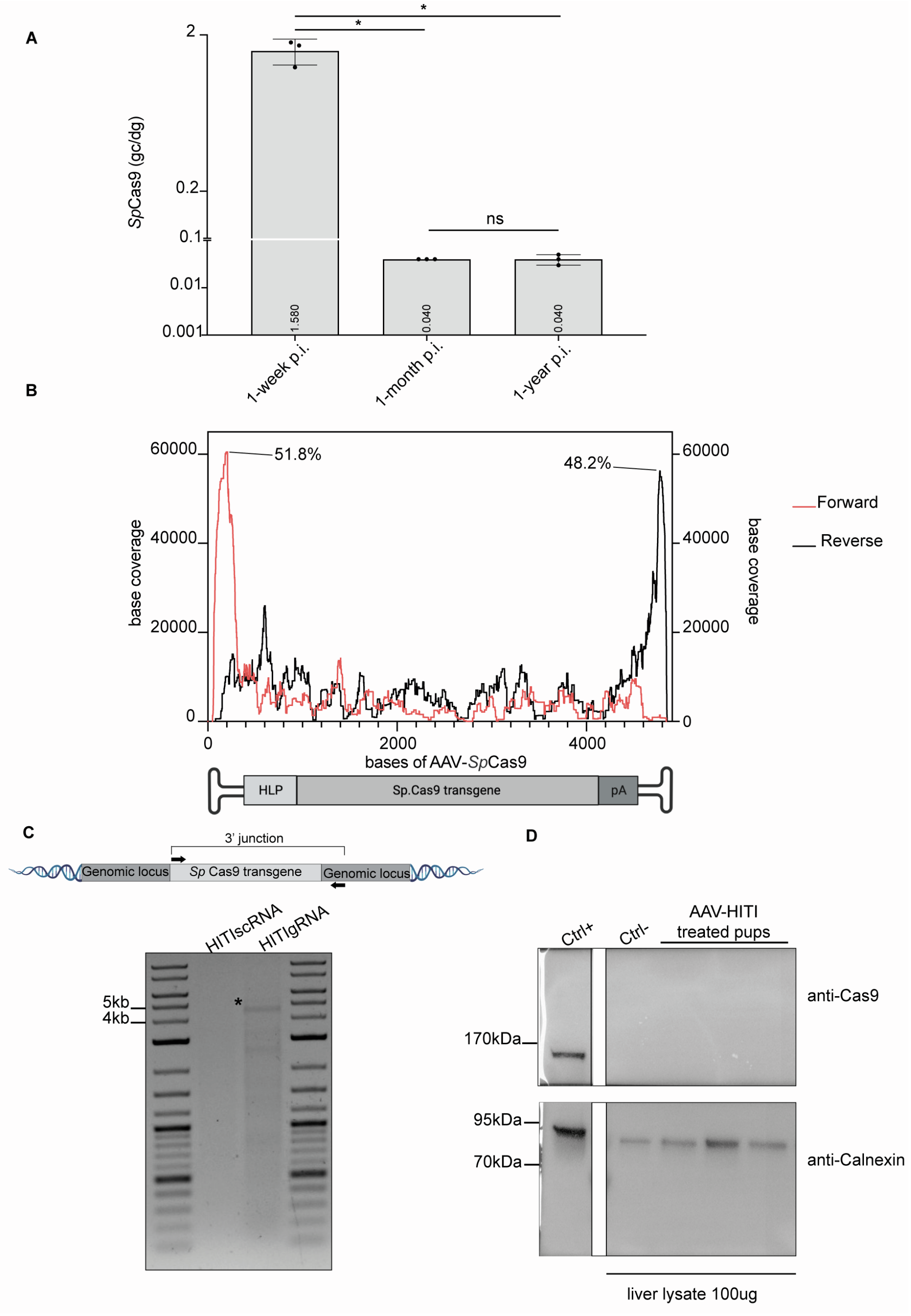

Figure S7. Evaluation of AAV-*Sp*Cas9 biodistribution, integration and expression in liver of newborn AAV-HITI-treated mice. Related to Figure 6. A) Real-Time PCR analysis of *Sp*.Cas9 vector genome in liver of AAV-HITIgRNA-treated newborn mice at different timepoints. Data are represented as mean ± standard deviation of Cas9 genome copies/diploid genomes (gc/dg). At each timepoint N=3 samples were used for the analysis. Statistical differences were assessed by Ordinary one-way ANOVA Test followed by Tukey's multiple comparisons test. P-value * = 0.0424

between 1-week and 1-month; p-value * = 0.0183 between 1-week and 1-year; p-value > 0.999 between 1-month and 1-year. **B**) CAST-Seq based single base coverage of AAV-*Sp.*Cas9 integration events. For each base, the read coverage of the forward and reverse strand is shown. The relative frequency of capturing of the 5' and 3' ITR regions as determined by peak height is indicated. **C**) Schematic of the PCR analysis to detect full-length *Sp*Cas9 vector genome integration. Black arrows indicate the primers designed to amplify the 3'junction in the forward orientation: the forward primer was designed to bind in the HA-tag preceding the *Sp*Cas9 transgene; the reverse primer binds in the endogenous locus. The expected product size is ~ 4.5kb. **D**) Western blot analysis performed on liver lysates (100µg) extracted from AAV-HITI-treated mice 1-year after neonatal administration (AFgRNA N=3). Positive control (Ctrl+): cell lysate transiently transfected with a plasmid carrying the *Sp*Cas9. Negative control (Ctrl-): liver lysed obtained from a mouse left untreated.

Figure S8

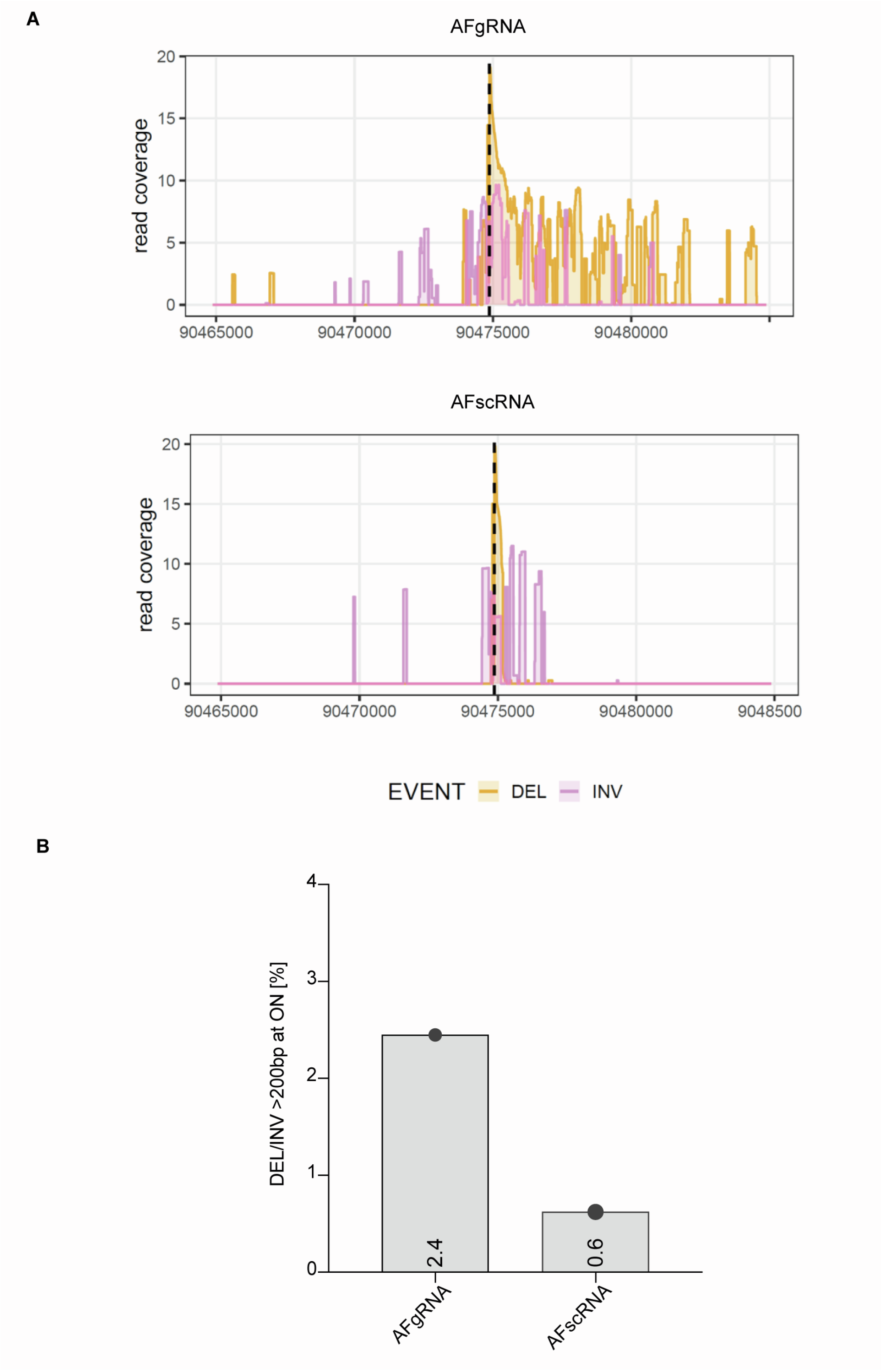

Figure S8. Assessment of on-Target aberrations in liver samples from AAV-HITI-treated mice. Related to Figure 6. A) Coverage plots showing deletions (DEL) and inversions (INV) within +/-10 kb of the on-target site in MPS VI mice treated as newborn with AAV-HITI at high doses (AFgRNA and AFscRNA) as detected by CAST-Seq. **B**) Quantification of CAST-Seq reads in A at a distance of more than 200 bp away from the cut site (ON), indicative of gross chromosomal aberrations such as deletions (DEL) and inversions (INV).

Figure S9

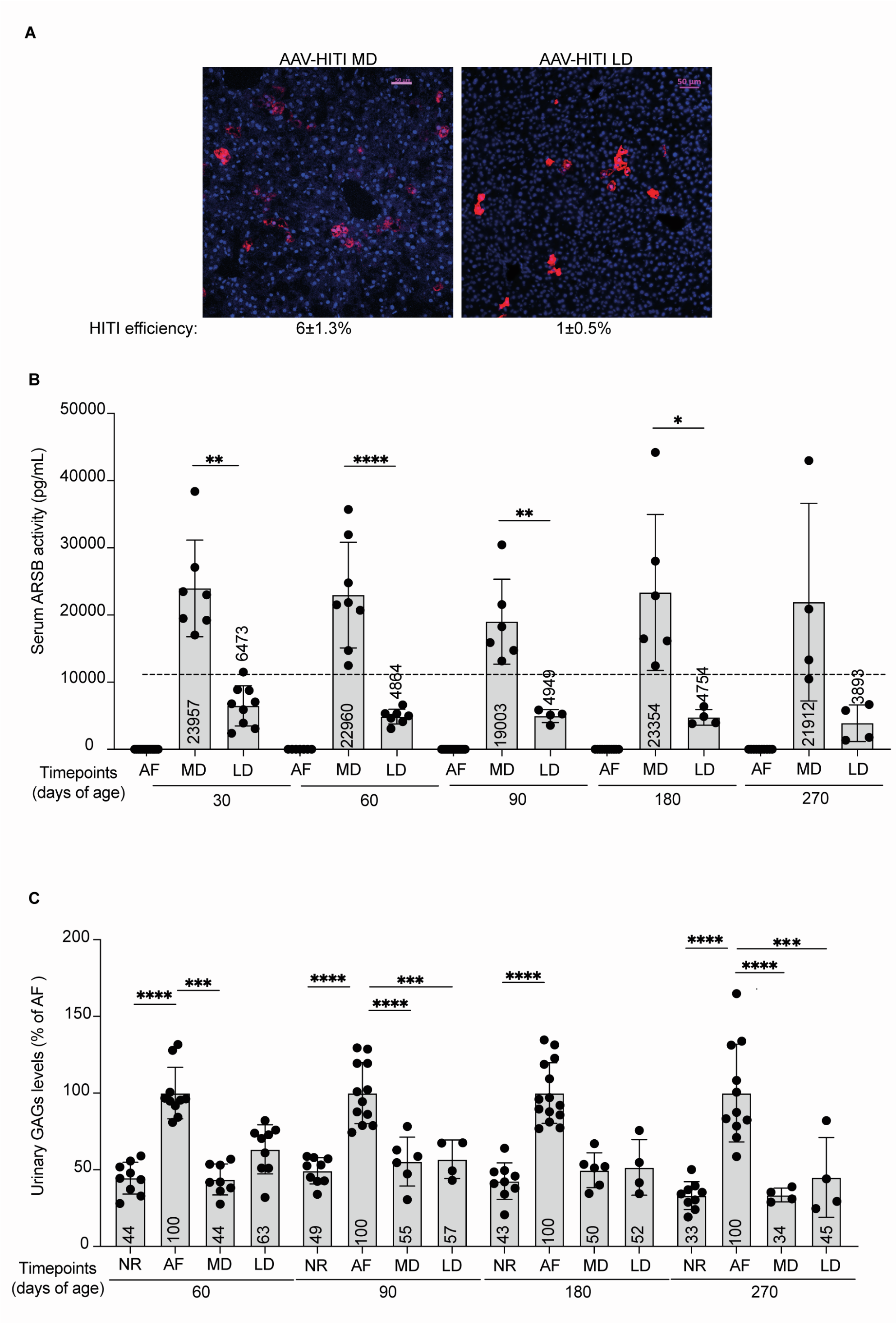

Figure S9. Liver-directed AAV-HITI at various doses in newborn MPS VI mice. Related to Figure 2. A) Representative fluorescence microscopy images of OCT liver cryo-sections from mice injected with AAV-HITI at different doses. Wild-type mice were treated with a medium dose (MD N=5, 3.9x10^13 total GC/kg) or a low dose (LD N=5, 1.2x10^13 total GC/kg) of AAV-HITIgRNA vectors. The percentage of Ds-Red positive hepatocytes (HITI efficiency) is reported. Scale bar 50μm. **B**) MPS VI mice were either left untreated (AF, N=14) or treated with a medium

dose (MD, N= 8) or a low dose (LD, N= 9), as in (**A**), of AAV-HITI-gRNA (AFgRNA) and serum ARSB activity was analysed at different timepoints after administration. Dotted line corresponds to normal serum ARSB activity (11825±334 pg/mL; Alliegro et al., 2016). Statistical differences were assessed by the ordinary one-way ANOVA and Tukey's multiple comparisons test; at p30 p-value ** =0.001; at p60 p-value **** < 0.001; at p90 p-value ** = 0.0052; at p180 p-value * =0.0302. **C**) Urinary GAGs were measured at different timepoints in MPS VI mice treated with either a medium (MD, N=8) or low dose (LD, N=9), of AAV-HITI-gRNA as in (**A**), or left untreated (AF, N=14); NR, N=9, normal mice. Values are reported as a percentage of GAGs levels in untreated MPS VI mice (% of AF). Statistical differences were assessed by Kruskal-Wallis Test and Dunn's multiple comparisons test at p60 p-value **** = <0.0001 between NR and AF; p60 p value ***= 0.0026 between AF and MD. Statistical differences were assessed by Welch ANOVA Test and Dunnett's T3 multiple comparisons test at p90 p-value **** = <0.0001 between NR and AF; p90 p-value ****= 0.0033 between AF and MD; p90 p-value ***= 0.0082 between AF and LD. Statistical differences were assessed by Kruskal-Wallis Test and Dunn's multiple comparisons test at p180. P-value ****= 0.0005 between NR and AF; at p270 p-value ****= < 0.0001 between AF and MD; at p270 p-value ***= 0.001 between AF and LD. Each dot corresponds to a single animal within each group at different time points. **B-C**) The differences in the number of analyzed samples within the same group of treatment at different timepoints was due to sample availability. All Data are represented as mean ± standard deviation.

Figure S10

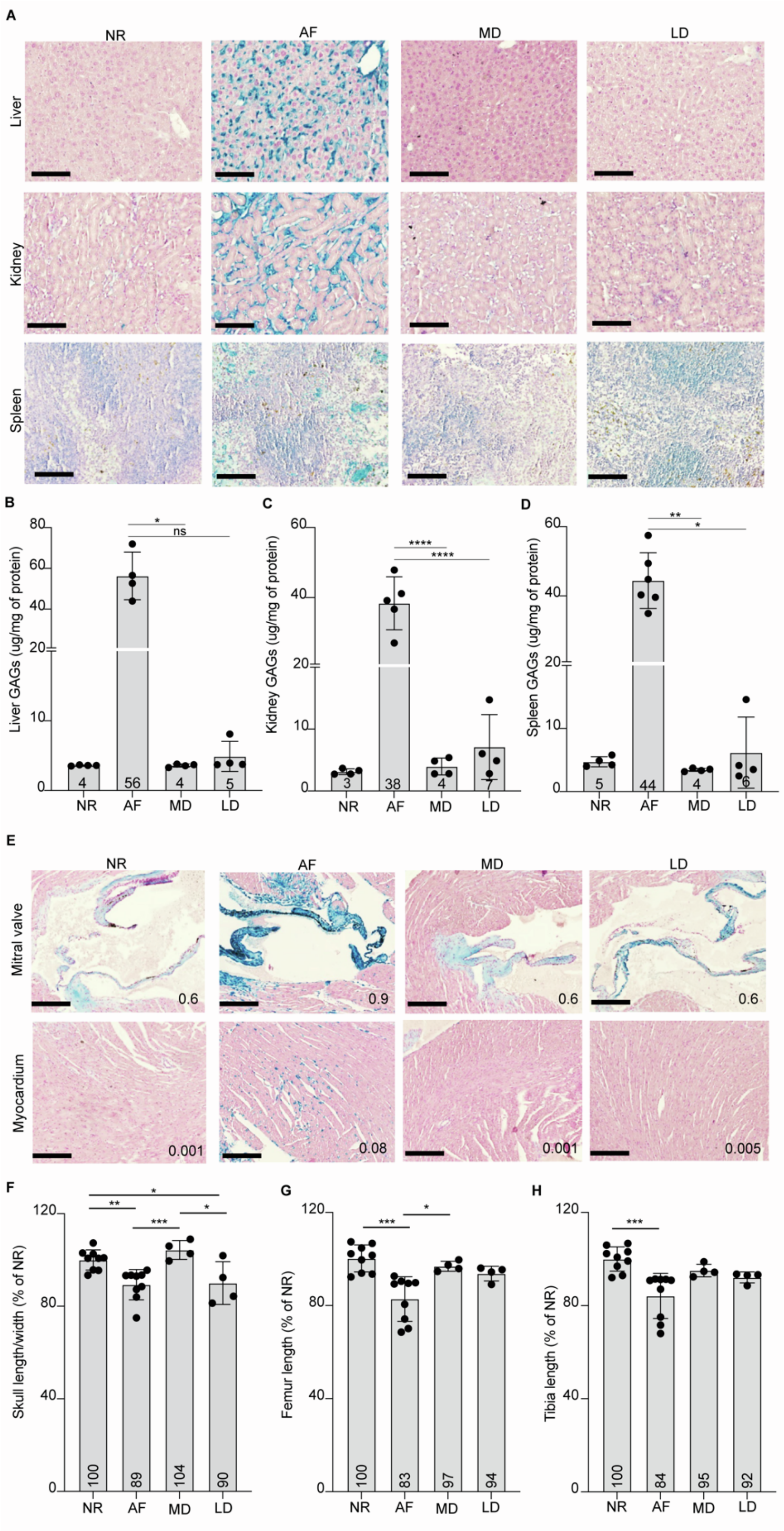

Figure S10. Efficacy of AAV-HITI at various doses in newborn MPS VI mice. Related to Figure 3. A) Alcian blue staining of histological sections from liver, kidney, spleen. Scale bar 50µm. Quantification of GAGs in liver (**B**), kidney

(**C**), and spleen (**D**). NR, unaffected untreated mice; AF (liver N=4; kidney N=5; spleen N=6), affected MPS VI mice, and AFgRNA MD (N=4), affected MPS VI mice treated with AAV-HITI-gRNA at medium doses; AFgRNA LD (N=4), affected MPS VI mice treated with AAV-HITI-gRNA at low doses **E**) Alcian blue staining of histological sections from mitral heart valve and myocardium. Alcian Blue quantifications in the mitral heart valve and in myocardium are reported as Alcian blue positive area/total area. Scale bar 50μm. NR, unaffected untreated mice; AF (N=6), affected MPS VI mice, and AFgRNA MD (N=4), affected MPS VI mice treated with AAV-HITI-gRNA at medium doses; AFgRNA LD (N=4), affected MPS VI mice treated with AAV-HITI-gRNA at low doses. **F-H**) Measurement of skull length/width ratio (**F**), femur (**G**) and tibia (**H**) lengths; data are reported as the percentage of normal length (% of NR). NR, unaffected untreated mice; AF (N= 9), affected MPS VI mice, and AFgRNA MD (N=4,), affected MPS VI mice treated with AAV-HITI-gRNA at medium dose; and AFgRNA LD (N=4,), affected MPS VI mice treated with AAV-HITI-gRNA at low doses. Statistical differences were assessed by ordinary one-way ANOVA and Tukey's multiple comparisons test: **B**) p-value * = 0.0138 between NR and AF; p-value >0.9999 between NR and AFgRNA MD; p-value * = 0.0398 between AF and AFgRNA MD; p-value =0.5656 between NR and AFgRNA LD; p-value >0.9999 between AF and AFgRNA LD; p-value >0.9999 between AFgRNA MD and AFgRNALD . **C**) P-value **** <0.0001 between NR and AF; p-value = 0.9949 between NR and AFgRNA MD; p-value **** <0.0001 between AF and AFgRNA MD; p-value = between NR and AFgRNA LD; p-value = 0.6712 between AF and AFgRNA LD; p-value 0.8037 between AFgRNA MD and AFgRNALD. **D**) P-value = 0.9858 between NR and AFgRNA MD; p-value **= 0.0051 between AF and AFgRNA MD; p-value >0.9999 between NR and AFgRNA LD; p-value* =0.0436 between AF and AFgRNA LD; p-value >0.9999 between AFgRNA MD and AFgRNALD. **F**) P-value** =0.0026 between NR and AF; p-value =0.6948 between NR and AFgRNA MD; p-value*** = 0.0009 between AF and AFgRNA MD; p-value *= 0.0401 between NR and AFgRNA LD; p-value =0.9995 between AF and AFgRNA LD; p-value* =0.0421 between AFgRNA MD and AFgRNA LD. **G**) p-value*** =0.0008 between NR and AF; p-value >0.9999 between NR and AFgRNA MD; p-value *= 0.0394 between AF and AFgRNA MD; p-value >0.9999 between NR and AFgRNA LD; p-value =0.6061 between AF and AFgRNA LD; p-value >0.9999 between AFgRNA MD and AFgRNALD. **H**) p-value*** =0.0001 between NR and AF; p-value >0.9999 between NR and AFgRNA MD; p-value =0.1701 between AF and AFgRNA MD; p-value = 0.3631between NR and AFgRNA LD; p-value >0.9999 between AF and AFgRNA LD; p-value >0.9999 between AFgRNA MD and AFgRNA LD.

Figure S11

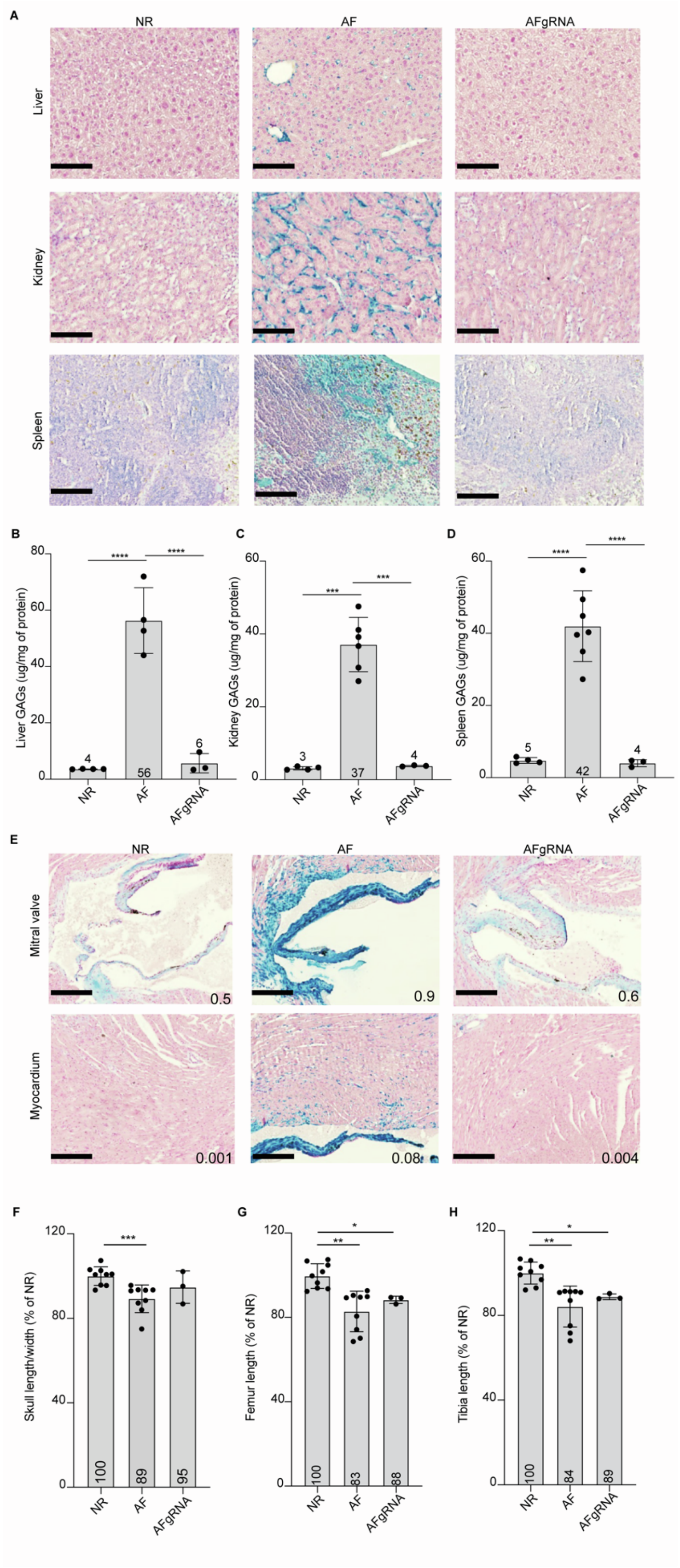
A
NR
AF
AFgRNA
Liver
Kidney
Spleen
B
Liver GAGs (ug/mg of protein)
C
Kidney GAGs (ug/mg of protein)
D
Spleen GAGs (ug/mg of protein)
E
Mitral valve
Myocardium
0.5
0.9
0.6
0.001
0.08
0.004
F
Skull length/width (% of NR)
G
Femur length (% of NR)
H
Tibia length (% of NR)

Figure S11. Efficacy of AAV-HITI in adult MPS VI mice. Related to Figure 7. A) Alcian blue staining of histological sections from liver, kidney, spleen. Scale bar 50µm. Quantification of GAGs in liver (**B**), kidney (**C**), and spleen (**D**). **E**) Alcian Blue quantification in the mitral heart valve and in myocardium are reported as Alcian blue positive area/total area. Scale bar 50µm. **F-H**) Measurement of skull length/width ratio (**F**), femur (**G**) and tibia (**H**) lengths; data are reported as the percentage of normal length (% of NR). NR (N=9), unaffected untreated mice; AF (N=9), affected MPS VI mice, and AFgRNA (N=3), affected MPS VI mice treated with AAV-HITI-gRNA at low doses; In **B**) NR, N=4; AF N=4; AFgRNA, N=3. In **C**) NR, N=4; AF N=6; AFgRNA, N=3. In **D**) NR, N=4; AF N=7; AFgRNA, N=3. Statistical differences were assessed by ordinary one-way ANOVA and Tukey's multiple comparisons test. **B)** P-value **** <0.0001 between NR and AF; p-value = 0.9280 between NR and AFgRNA; p-value **** <0.0001 between AF and AFgRNA. **C**) P-value ***= 0.0003 between NR and AF; p-value = 0.1385 between NR and AFgRNA; p-value ***= 0.0003 between AF and AFgRNA. **D**) P-value **** <0.0001 between NR and AF; p-value = 0.9904 between NR and AFgRNA; p-value **** <0.0001 between AF and AFgRNA. **F**) p-value *** =0.00029 between NR and AF; p-value = 0.3868 between NR and AFgRNA; p-value = 0.3610 between AF and AFgRNA. **G**) p-value** =0.0014 between NR and AF; p-value * = 0.0322 between NR and AFgRNA; p-value >0.9999 between AF and AFgRNA. **H**) p-value **=0.0012 between NR and AF; p-value * =0.0235 between NR and AFgRNA; p-value >0.9999 between AF and AFgRNA.

Table S2

Primers	Sequence (5'-3')
**5' junction forward**	CACGTGGTCAGGTGTAGCTC
**5' junction reverse**	TGGAGAGAAAGGCAAAGTGGA

Table S2. 5' short junction primers. Related to Figure 4 and STAR Methods. Primers used to PCR amplify the 5' junction upon donor DNA integration.

Table S3

Primers	Sequence (5'-3')
**Nanopore LR-Seq 5' junction forward**	CCACCAATCTAGATGTTGTTC
**Nanopore LR-Seq 5' junction reverse**	CCCAGAATAGAATGACACCTACTCA
**Nanopore LR-Seq 3' junction forward**	**GAGTCTTGTGTCCCAGTTACCAGG**GGTCTTACTGACATCCACTTTG
**Nanopore LR-Seq 3' junction reverse**	**CCCTGGGACGTAGGAATCCACGCC**GGTAACTGTCCATTCATGTG

Table S3. Nanopore long-read (LR) sequencing primers. Related to Figure 5 and STAR Methods. Sequences in bold are barcodes to enable multiplexed sequencing on the same flow cell.

Table S4

Primers	Sequence (5'-3')
**3' junction forward**	CCCATACGATGTTCCAGATTAC
**3' junction reverse**	GGTAACTGTCCATTCATGTG

Table S4. *Sp*Cas9 3'junction primers. Related to STAR Methods. Primers used to PCR amplify the 3' junction to assess full-length *Sp*Cas9 integration.

Table S5

Target	Function		Sequence (5'-3')
mAlb-HITI	CAST-Seq PCRI	bait	CCACCAATCTAGATGTTGTTC
	CAST-Seq PCRI	decoy fwd	GACACAGAAGAGCATAGTTAGA
	CAST-Seq PCRI	decoy rev	CAAATTTGAAACCAAATAGTGATAATAGG
	CAST-Seq PCRI	decoy HITI	GATGTCAGTAAGACCAATAGGTG
	CAST-Seq PCRII	bait nested	GACTGGAGTTCAGACGTGTGCTCTTCCGATCTGTTGTTCTCTACGTAACTGTAATAGAAAC
Linker primers	CAST-Seq PCRI	prey	GTAATACGACTCACTATAGGGC
	CAST-Seq PCRII	prey nested	ACACTCTACACTCTTTCCCTACACGACGCTCTTCCGATCTAGGGCTCCGCTTAAGGGAC
Linker oligos	CAST-Seq positive strand		GTAATACGACTCACTATAGGGCTCCGCTTAAGGGACT
	CAST-Seq negative strand		P-GTCCCTTAAGCGGAGC-NH3

Table S5. CAST-Seq primers. Related to Figure 6 and STAR Methods. Primers used to perform CAST-Seq.

Table S6

gRNA	Region	Position	(5'-3') gRNA + PAM sequences	Mismatches	Off-target CDF score
ON- target	Intron: albumin	chr5: 90622727-90622747:-	GTATTTAATAGGCAGCAGTG TGG	-	-
OFF-target 1	Intron: Rik	chr5:151333345-151333367:-	**T**TA**C**TTAATA**A**GCAGCAGTG TGG<br>* * *	3	0.647
OFF-target 2	Intron: Lrr 1	chr12: 69224137-69224159:-	GT**T**TTTAA**A**A**A**GCAG**A**AGTG GGG<br>* * * *	4	0.646
OFF-target 3	Intergenic: Ppp1r3c-Tnks2	chr19: 36774036-36774058:-	**T**TAT**C**TAATAG**A**CAGCA**A**TG CGG<br>* * * *	4	0.646
OFF-target 4	Intron: Zim2	chr7: 6660686-6660708:+	G**A**ATTT**G**ATAG**A**CAGCAGTG GGG<br>* * *	3	0.557
OFF-target 5	Intron:Slc39a12	chr2:14426612-14426634:+	GTATTTA**GA**AGGCAGCAGT**T** TGG<br>** *	3	0.476
OFF-target 6	Intron: Gstcd	chr3:132751735-132751757:-	**AA**ATTT**G**AT**T**GGCAGCAGTG TGG<br>** * *	4	0.474
OFF-target 7	Intron: Kcnc1	chr7: 46060812-46060834:-	GTATTTAA**A**AGGC**T**G**A**AGT**A** AGG<br>* * * *	4	0.464
OFF-target 8	Intergenic: Rik/Lhfpl3-Lhfpl3	chr5: 23268978-23269000:+	**A**TATT**C**AA**GT**GGCAGCAGTG AGG<br>* * **	4	0.446
OFF-target 9	Intron: Dpyd	chr3:119141669-119141691:+	**A**TATTTAATAGGCA**A**CA**TT**T AGG<br>* * * *	4	0.395
OFF target 10	Intron: HCn2	chr10: 79561901-79561923:+	G**G**ATT**C**A**G**TAGGCAGCAGT**T** GGG<br>* * * *	4	0.392

Table S6. Off-Targets. Related to Figure 6. The top-ten predicted off-target sites were selected with the CRISPOR web tool. The mouse genome used of reference is the (GRCm39/mm39).

Table S7

Primers	Sequence (5’-3’)
Off-target 1 forward	TGTGGTTGCTGGGATTTGAAC
Off -target 1 reverse	ACTCCTCAGAAGGGTAATTGTAG
Off -target 2 forward	CACAGTAGGAATGCCATAGAAG
Off -target 2 reverse	TGTGGTAGGTAGGGAAAGAAC
Off-target 3 forward	GACTCCTTCACAAGGGATCACTTG
Off-target 3 reverse	AGTGTTCCAGTCTAAAGCACT
Off-target 4 forward	GCATGCTTAACTGCTGAGGT
Off-target 4 reverse	AGTAAGCTACACATGTAACAGCGACTAG
Off-target 5 forward	AATGCTGCAGGTCTGAGTGA
Off-target 5 reverse	ATAACTTCCGAGGTCTACTGCT
Off-target 6 forward	TGTAAAGTTAGCTGCTGACTG
Off-target 6 reverse	CTAACATCAACTTCAAGCACAGGAAG
Off-target 7 forward	TTGCTTGCTGTTTCCTGTGA
Off-target 7 reverse	CTCTGAGGTGTACTTTGTACCT
Off-target 8 forward	ACCCTCCCCTGTGACTTACT
Off-target 8 reverse	CCACCCTACTAAGCTATGCT
Off-target 9 forward	GAATAAGATGAGGCCAGAGTA
Off-target 9 reverse	TTCACGTTCATCCAAGGTC
Off-target 10 forward	AGGACTTGGGCAGGATAAA
Off-target 10 reverse	TGTGACCATGAGAGTCCCAG

Table S7. Off-target primers. Related to Figure 6. List of the primers used to PCR amplify the predicted off-target loci.
