# Supplementary material for: Safe and effective liver-directed AAV-mediated homology-independent targeted integration in mouse models of inherited diseases
Source: Cell Rep Med. 2024 Jun 18;5(7):101619. doi: 10.1016/j.xcrm.2024.101619 (PMC11293346; doi:10.1016/j.xcrm.2024.101619)
Supplement: Document S2. Article plus supplemental information [file mmc3.pdf]

Cell Reports
Medicine

Article

Safe and effective liver-directed AAV-mediated homology-independent targeted integration in mouse models of inherited diseases

Graphical abstract

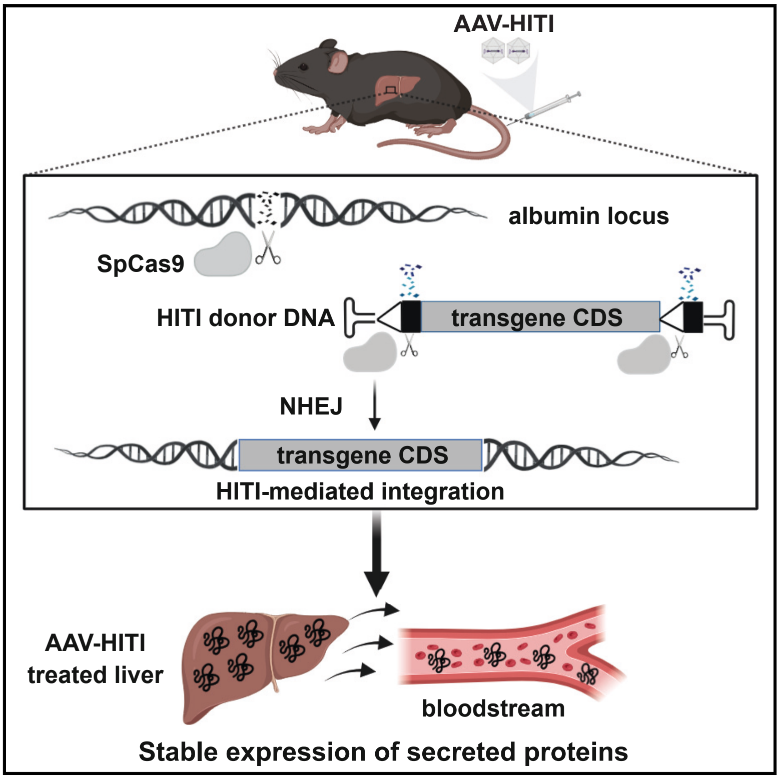

Highlights

- AAV-HITI provides sustained transgene expression from newborn and adult liver
- Liver-directed AAV-HITI is effective in mouse models of inherited diseases
- AAV-HITI results in undetectable off-targets and large gene rearrangements
- AAV-HITI is safe at the doses tested up to 1 year after AAV-HITI delivery

Authors

Federica Esposito, Fabio Dell'Aquila, Manuel Rhiel, ..., Ivana Trapani, Toni Cathomen, Alberto Auricchio

Correspondence

auricchio@tigem.it

In brief

Esposito et al. explore the therapeutic potential of liver-directed adeno-associated viral vector-mediated homology-independent targeted integration (AAV-HITI) through CRISPR-Cas9 at the mouse albumin locus. Effective integration yields stable therapeutic protein levels in mouse models of inherited diseases without adverse effects, supporting AAV-HITI as a safe and efficient liver-directed knockin strategy.

Esposito et al., 2024, Cell Reports Medicine *5*, 101619
July 16, 2024 © 2024 The Authors. Published by Elsevier Inc.
https://doi.org/10.1016/j.xcrm.2024.101619

CellPress

Cell Reports Medicine

CellPress
OPEN ACCESS

Article

Safe and effective liver-directed AAV-mediated homology-independent targeted integration in mouse models of inherited diseases

Federica Esposito,[1] Fabio Dell'Aquila,[1,2] Manuel Rhiel,[3,4] Stefano Auricchio,[1] Kay Ole Chmielewski,[3,4,5] Geoffroy Andrieux,[6,7] Rita Ferla,[1] Paula Sureda Horrach,[1] Arjun Padmanabhan,[1] Roberto Di Cunto,[1] Simone Notaro,[1] Manel Llado Santeularia,[1] Melanie Boerries,[6,7,8] Margherita Dell'Anno,[1] Edoardo Nusco,[1] Agnese Padula,[1] Sofia Nutarelli,[9] Tatjana I. Cornu,[3,4,7] Nicolina Cristina Sorrentino,[1,10] Pasquale Piccolo,[1] Ivana Trapani,[1,2] Toni Cathomen,[3,4,7,8] and Alberto Auricchio[1,11,12,*]

[1]Telethon Institute of Genetics and Medicine (TIGEM), Pozzuoli, Italy
[2]Medical Genetics, Department of Advanced Biomedical Sciences, University of Naples Federico II, Naples, Italy
[3]Institute for Transfusion Medicine and Gene Therapy, Medical Center – University of Freiburg, Freiburg, Germany
[4]Center for Chronic Immunodeficiency (CCI), Medical Center – University of Freiburg, Freiburg, Germany
[5]PhD Program, Faculty of Biology, University of Freiburg, Freiburg, Germany
[6]Institute of Medical Bioinformatics and Systems Medicine, Medical Center – University of Freiburg, Freiburg, Germany
[7]Faculty of Medicine, University of Freiburg, Freiburg, Germany
[8]German Cancer Consortium (DKTK), Partner site Freiburg, a partnership between DKFZ and Medical Center - University of Freiburg, Freiburg, Germany
[9]Department of Life Science and Public Health, Catholic University of the Sacred Heart, Rome, Italy
[10]Department of Clinical Medicine and Surgery, University of Naples Federico II, Naples, Italy
[11]Gene Therapy Joint lab, Dept. of Advanced Biomedical Sciences and Dept. of Translational Medicine, University of Naples "Federico II", Naples, Italy
[12]Lead contact
*Correspondence: auricchio@tigem.it
https://doi.org/10.1016/j.xcrm.2024.101619

SUMMARY

Liver-directed adeno-associated viral (AAV) vector-mediated homology-independent targeted integration (AAV-HITI) by CRISPR-Cas9 at the highly transcribed albumin locus is under investigation to provide sustained transgene expression following neonatal treatment. We show that targeting the 3′ end of the albumin locus results in productive integration in about 15% of mouse hepatocytes achieving therapeutic levels of systemic proteins in two mouse models of inherited diseases. We demonstrate that full-length HITI donor DNA is preferentially integrated upon nuclease cleavage and that, despite partial AAV genome integrations in the target locus, no gross chromosomal rearrangements or insertions/deletions at off-target sites are found. In line with this, no evidence of hepatocellular carcinoma is observed within the 1-year follow-up. Finally, AAV-HITI is effective at vector doses considered safe if directly translated to humans providing therapeutic efficacy in the adult liver in addition to newborn. Overall, our data support the development of this liver-directed AAV-based knockin strategy.

INTRODUCTION

Adeno-associated viral (AAV) vectors are considered the most effective tool for *in vivo* gene therapy due to their safety, efficacy, and long-term therapeutical transgene expression.[1–3] In AAV-based liver-directed gene therapy, a single intravenous AAV administration has been demonstrated to be sufficient to convert hepatocytes into a factory for efficient and sustained transgene expression and secretion of proteins into the bloodstream, providing long-term therapeutic effects in both preclinical studies and humans.[4–9] Despite their therapeutic potential, the non-integrative nature of AAV genomes excludes the use of AAV vectors from neonatal treatments,[10–13] which is the preferred stage of intervention for diseases such as early-onset inborn errors of metabolism. Liver growth in young subjects leads to AAV genome loss[14] over time resulting in the decline of the therapeutic efficacy following cell division.[1] For this reason, patients under 4 years of age were excluded from our recent successfully completed phase 1/2 clinical trial of AAV-based liver gene therapy for mucopolysaccharidosis type VI (MPS VI)[5] (Database: ClinicalTrials.gov, number NCT03173521). Furthermore, antibodies against the AAV vector capsid that are generated after the first administration may prevent a second round of treatment.[1,15–19] To address these shortcomings, genome editing via CRISPR-Cas nucleases alone or in combination with knockin strategies is being explored

© 2024 The Authors. Published by Elsevier Inc.
This is an open access article under the CC BY license (http://creativecommons.org/licenses/by/4.0/).

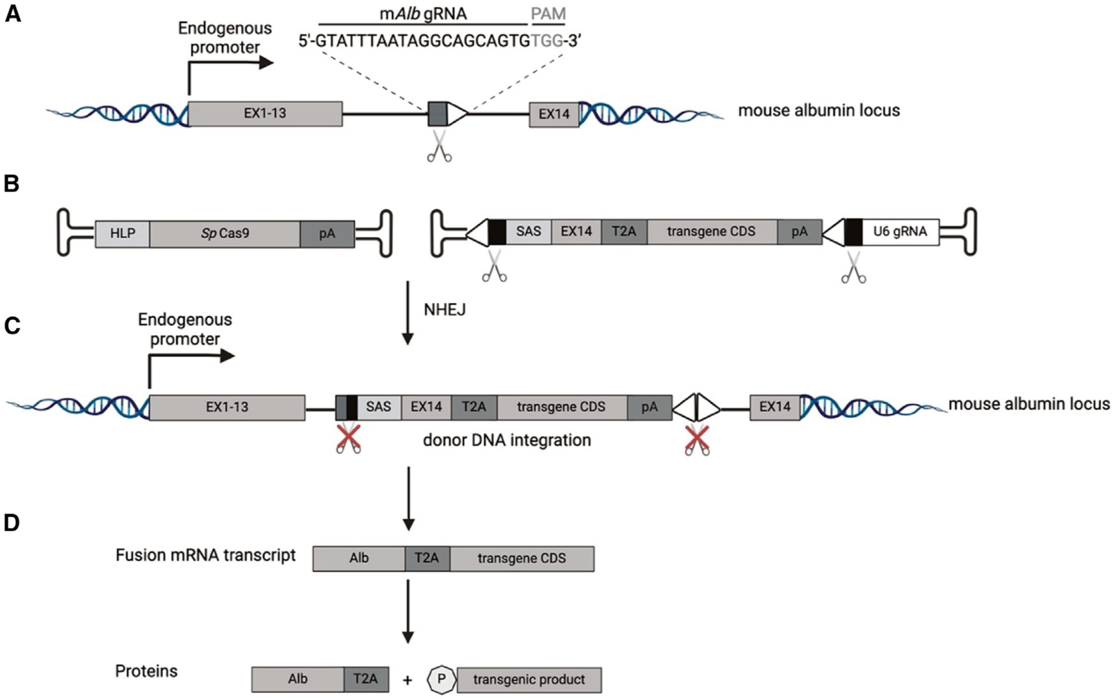

Figure 1. Schematic representation of homology-independent targeted integration (HITI)

(A) The on-target mouse albumin locus and the gRNA sequence designed within the intron 13 are depicted. EX1-13, mouse albumin exons; EX14, last mouse albumin exon;PAM, protospacer adjacent motif.

(B) Schematic representation of the two AAVs. One AAV carries the nuclease *Sp*Cas9 under control of the small hybrid liver-specific promoter (HLP). The other AAV contains the HITI donor DNA with the desired promoter-less transgene coding sequence (CDS). SAS, splicing acceptor signal; EX14, mouse albumin exon 14; T2A, Thosea asigna virus 2A skipping peptide; pA, polyadenylation signal (synthetic or bovine growth hormone); U6 gRNA, expression cassette for the gRNA (depicted) or the scRNA sequence; EX1-13, mouse albumin exons 1–13; gRNA and PAM sequences are represented with black (inverted gRNA sequences at the extremities of the donor DNA) and gray (within the mouse albumin locus) boxes and white triangle, respectively.

(C) The non-homologous end-joining (NHEJ) repair pathway of the cell leads to the integration of the cleaved donor DNA at the on-target site.

(D) Upon transgene integration a single fusion transcript is produced, and this results in the expression of both a modified albumin (Alb-2A) and a therapeutic transgene product containing a proline (P) residue at its N terminus.

extensively.[1,20–30] In particular, homology-independent targeted integration (HITI) of therapeutic transgenes[21,25,31–34] into the mouse albumin (m*Alb*) locus, which occurs via the cell cycle-independent non-homologous end-joining (NHEJ) repair machinery, is being considered to achieve stable expression following newborn liver gene therapy.[25,28,35–38] Here, we show that AAV-HITI directed to the 3′ end of the albumin locus results in the integration of the donor DNA after its processing by CRISPR-Cas9 as expected based on HITI design. This provides significant therapeutic benefit in the absence of major off-target (OT) and toxic effects in different mouse models of inherited human diseases.

RESULTS

Liver-directed AAV-HITI results in robust transgene expression following neonatal treatment

Our AAV vector-based CRISPR-Cas9-mediated HITI (AAV-HITI) approach was designed to target intron 13 of the m*Alb* locus, similarly to previous reports[34] (Figure 1A). The system relies on the co-delivery of two different AAV8 vectors. AAV8 has high liver tropism[39,40] and has been safely and effectively used in various AAV-liver-directed genome editing preclinical studies[21,30,41] as well as by us and others in liver-directed gene therapy clinical trials.[5,8] One AAV delivers the large *S. pyogenes* Cas9 (*Sp*Cas9) nuclease together with a small hybrid liver-specific promoter (HLP),[21,42,43] while the other carries the HITI donor DNA which is designed to contain a synthetic splicing acceptor signal (SAS) followed by the last m*Alb* exon (exon 14), the Thosea asigna virus 2A skipping peptide (T2A), and the promoter-less coding sequence (CDS) of a desired transgene together with a polyadenylation signal (pA) and the guide (g) RNA (or scramble RNA [scRNA]) expression cassette including the U6 promoter (Figure 1B). As previously described,[21,32,33] the HITI donor DNA is flanked at its 5′ and 3′ extremities by the same gRNA sequence of the endogenous target site (m*Alb* intron 13) but in an inverted orientation. Upon CRISPR-Cas9-mediated cleavage at both the endogenous locus and the extremities of the HITI donor DNA, the NHEJ repair pathway of the cell promotes donor DNA integration at the nuclease-induced double-strand breaks (DSBs) in the endogenous locus[21,32–34] (Figure 1C). Once proper integration has occurred, the therapeutic transgene is expressed under the control of the endogenous promoter. A single fusion mRNA transcript is generated; at the protein levels, this will result in the production of both a modified albumin (Alb-2A) and a

CellPress
OPEN ACCESS

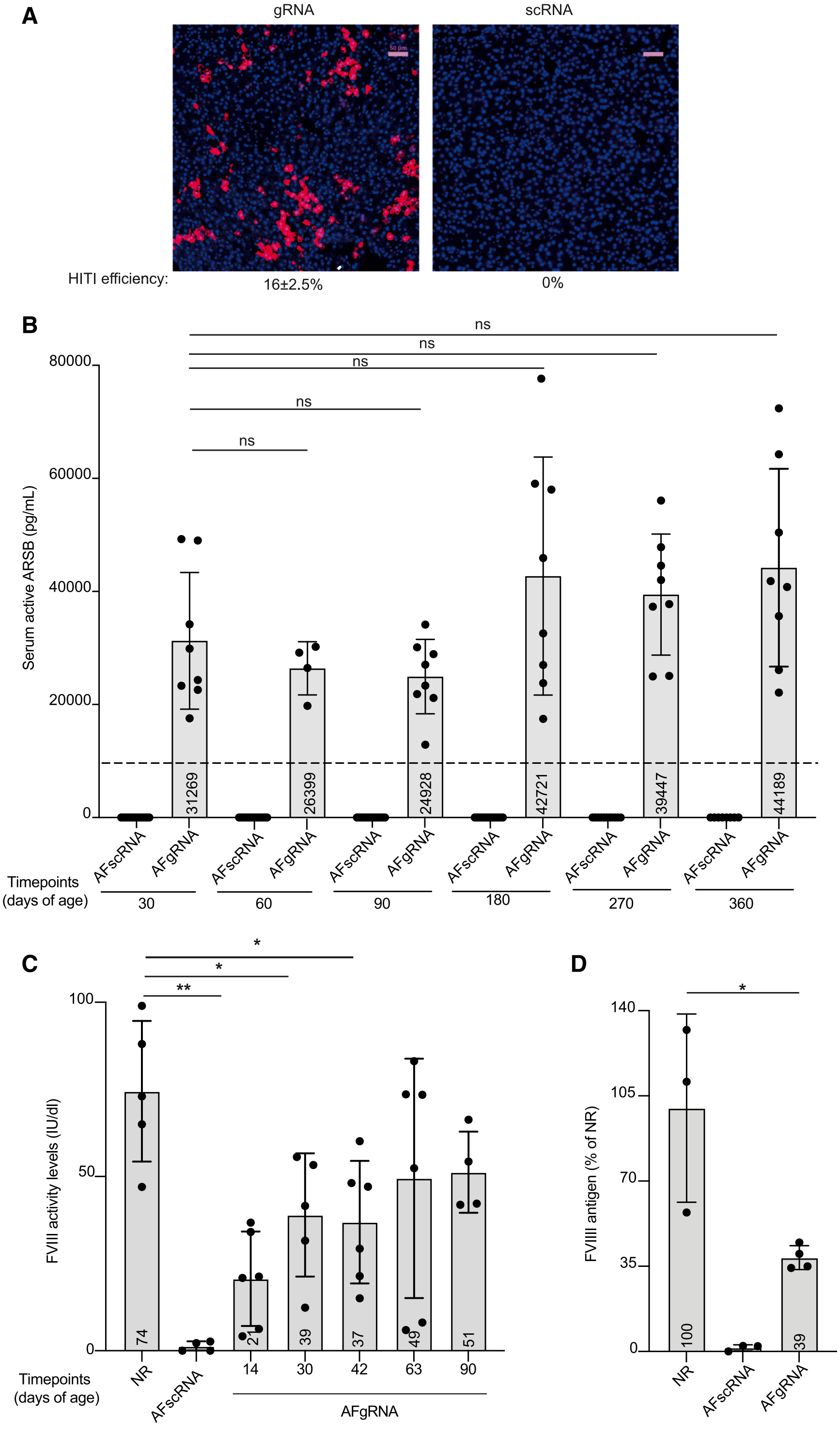

Figure 2. AAV-HITI-mediated transgene expression from newborn liver

(A) Representative fluorescence microscopy images of OCT liver cryo-sections from wild-type mice injected with AAV-HITI using the dsRed transgene (gRNA, $N = 5$ or scRNA, $N = 5$) at the total dose of 1.2×10^{14} total GCs/kg. HITI efficiency is reported below the images. Scale bar, 50 μm.

(B) Serum ARSB activity was analyzed at different time points after AAV-HITI treatment. AFgRNA, MPS VI mice treated with AAV-HITI-gRNA ($N = 8$); AFscRNA ($N = 14$ and $N = 8$ survived up to P360), MPS VI mice treated with AAV-HITI-scRNA. Dotted line corresponds to normal (NR) serum ARSB activity (NR = 11,825 ± 334 pg/mL; Alliegro et al., 2016[44]). ARSB measurements in all AFscRNA animals are equal to zero while all treated mice show ARSB activity levels higher than zero; therefore all the comparisons between the two groups are significant. No statistically significant differences were observed in AFgRNA-treated mice among the different time points. (B–C) Each dot corresponds to a single animal within each group at different time points.

(C) FVIII activity levels evaluated in HemA mice by chromogenic assay at different time points after AAV-HITI treatment. Statistical differences were assessed by ordinary one-way ANOVA test. **p = 0.0025, between normal (NR, $N = 5$) and AFgRNA ($N = 6$) at 14 days; *p = 0.0190, between NR and AFgRNA ($N = 5$, one sample was excluded) at 30 days; *p = 0.0389, between NR and AFgRNA ($N = 6$) at 42 days. No statistically significant differences were observed between NR and AFgRNA at the following time points.

(D) FVIII antigen levels measured in AFgRNA ($N = 4$) HemA mice at 90 days of age. *p = 0.0262 between NR and AFgRNA at 90 days. All data are represented as mean ± standard deviation. See also Figures S1, S2, and Table S1.

therapeutic transgene product containing a proline (P) residue at its N terminus (Figure 1D). To evaluate the efficiency of the system at targeting newborn hepatocytes, we used an AAV-HITI donor carrying the CDS of the *Discosoma* sp.*-Red* (dsRed) fluorescent protein together with the expression cassette encoding either the gRNA or an scRNA which served as a negative control (Table S1). Wild-type C57BL/6 mice were divided into two different treatment groups (gRNA or scRNA) and received a mixture of vectors at 1:1 ratio via temporal vein injection between post-natal days (P) 1 and 2 at a total dose of 1.2×10^{14} genome copies (GCs)/kg. Four weeks post-injection, fluorescent microscopy images from liver cryo-sections revealed that dsRed was highly expressed in livers of mice treated with gRNA (mean ± SD = 16% ± 2.5% dsRed-positive hepatocytes; Figures 2A and S1A) but not with scRNA. This indicates that our AAV-HITI approach provides robust transgene expression following neonatal delivery in mice. We then investigated the persistence of the AAV-HITI donor DNA following newborn delivery and found a significant drop after a month, as expected based on hepatocyte proliferation at that age, with similar very low levels at 1 and 12 months

after vector delivery (Figure S1B). We therefore hypothesized that the majority of the HITI donor DNA at 1 month after newborn delivery is integrated and performed a customized DNA hybridization analysis with a probe specific for the HITI donor DNA vector on liver cryo-sections to assess the rate of its integration. We found about 40% more positive hepatocyte nuclei in gRNA- than scRNA-treated samples, presumably resulting from HITI donor DNA integration whether full or partial (Figure S1C).

We next investigated the therapeutic potential of our strategy in two different mouse models of human inherited diseases. Liver-directed AAV-HITI efficacy was first assessed in a mouse model of MPS VI,[45] a lysosomal storage disorder due to arylsulfatase B (ARSB) deficiency. In this setting, the AAV-HITI donor vector contained the *ARSB* CDS in place of dsRed (Table S1). AAV-HITI vectors were co-delivered systemically in newborn MPS VI mice as described earlier, and the mice were monitored over a period of 1 year. Since ARSB is secreted into the bloodstream, it can be measured non-invasively[45] as a readout for AAV-HITI treatment. Starting from 1-month post-treatment, serum ARSB activity was found to be higher in AAV-HITI-gRNA-treated MPS VI (AFgRNA) than in wild-type mice[44] (Figure 2B) while AAV-HITI-scRNA-treated littermates (AFscRNA) had undetectable levels of ARSB at all time points (Figure 2B). Interestingly, ARSB activity in AFgRNA mice showed a non-statistically significant trend toward increase over time (Figure 2B). Next, we investigated whether following AAV-HITI serum albumin includes the T2A modification (Alb-2A) as predicted based on the AAV donor DNA design (Figure 1). Western blot analysis of AAV-HITI-treated sera samples collected at different time points confirmed the presence of Alb-2A of correct size (Figure S2A). In addition to MPS VI, AAV-HITI efficacy was assessed in a mouse model of hemophilia A (HemA), the most common X-linked bleeding disorder caused by mutations in the *F8* gene.[46] Since the full-length *F8* CDS (~7 kb) exceeds the cargo capacity (~4.9 kb) of the AAV vector, we designed an HITI donor DNA carrying the promoter-less CDS of a previously described human B domain-deleted (BDD) *F8* variant[47] (CodopV3 ~4.5 kb; Table S1). Newborn HemA male mice were injected systemically at P1–2 with AAV-HITI vectors (gRNA or scRNA) at a total dose of 3.9 × 10^{13} GCs/kg. Plasma samples were collected from AAV-HITI-treated HemA mice (AFgRNA and AFscRNA) and unaffected controls at different time points, and FVIII activity and FVIII antigen were measured. Therapeutic levels of FVIII were observed in plasma samples of AFgRNA- but not AFscRNA-treated mice up to 90 days of age by chromogenic assay[42,48] (Figure 2C), and FVIII protein levels were found to be around 40% of normal FVIII levels at 90 days post AAV-HITI delivery (Figure 2D). Lastly, Alb-2A expression was confirmed in AFgRNA mice at different time points by western blot analysis (Figure S2B).

Liver-directed AAV-HITI improves the phenotype of MPS VI and HemA mice

ARSB deficiency results in systemic abnormal storage and urinary excretion of glycosaminoglycans (GAGs). The measurement of urinary GAGs levels is considered a useful biomarker for MPS VI.[5,49,50] To evaluate AAV-HITI-mediated phenotypic improvement, we collected urine samples from treated mice and unaffected littermates at different time points. Normalized urinary GAG levels were found in urinary samples from AFgRNA-treated MPS VI mice starting from 3 months post-treatment (P90) until the last time point of the analysis (P360; Figure 3A). One-year post-treatment, MPS VI mice were sacrificed, and different organs were collected for further analysis. GAG levels in liver, kidney, and spleen lysates were normal in AFgRNA-treated MPS VI mice (Figure 3B). Accordingly, GAGs storage was significantly decreased in histological sections of these tissues (Figure S3) as well as in the heart mitral valve and myocardium of the AFgRNA-treated MPS VI mice (Figure 3C). Indeed, MPS VI patients suffer from heart failure, predominantly due to mitral valve insufficiency.[51] A further feature of MPS VI patients is skeletal abnormalities,[49,52] which have also been described in the mouse model of MPS VI.[53] Therefore, we assessed if AAV-HITI can improve these disease manifestations. Radiographic images from the AAV-HITI-treated MPS VI mice (AFgRNA or AFscRNA) and unaffected normal controls (NR) were used to evaluate the skull and long bones (femurs and tibias); male and female mice were considered separately given the sex-dependent growth differences. In AFgRNA-treated mice we observed an improvement in the skull width/length ratio compared to AFscRNA-treated mice of the same sex (Figure 3D). In AFgRNA-treated male mice we found a significant amelioration in the femur (Figure 3E) and tibia (Figure 3F) lengths. Altogether, our data indicate that AAV-HITI is effective following newborn delivery to MPS VI mice. Lastly, we assessed AAV-HITI efficacy in newborn AAV-HITI-treated (AFgRNA or AFscRNA) HemA mice by measuring the hemostasis activity by tail-clip and activated partial thromboplastin time (aPTT) assays.

We found that AFgRNA-treated male mice exhibited a significant reduction of bleeding time (Figure 3G) and blood loss (Figure 3H) at 63 days of age and improved coagulation compared to AFscRNA-treated mice at 90 days of age (Figure 3I). These findings support the use of AAV-HITI for therapies directed to newborn liver.

AAV-HITI at the on-target site

Having established the physiological benefits of AAV-HITI in two disease models, we thoroughly characterized AAV-HITI at the on-target site by molecular analysis and next-generation sequencing (NGS). Genomic DNA extracted from liver samples of AAV-HITI-treated MPS VI animals was used to evaluate *Sp*Cas9-gRNA efficiency 1-year post-treatment. For each treatment (gRNA or scRNA), a pool of 3 different DNA samples was analyzed by targeted amplicon NGS. Small insertions/deletions (indels) were observed in ~30% of reads from gRNA but not scRNA samples (~0.1%; Figure 4A). Interestingly, in ~1% (n = 603) of the total obtained reads (n = 61,260) from the gRNA DNA pool, we observed insertions longer than 10 nucleotides which were found to align with the AAV-HITI donor and the AAV-*Sp*Cas9 vector genomes. The vast majority of these insertions originated from the inverted-terminal repeat (ITR) regions (Figure 4B). We then investigated if the AAV donor DNA was integrated before or after being processed by CRISPR-Cas9 (therefore with or without the ITRs, respectively). To this end, we generated, as control, a donor DNA lacking the inverted gRNA sites at both its 5′ and 3′ extremities (donor DNA without

CellPress
OPEN ACCESS

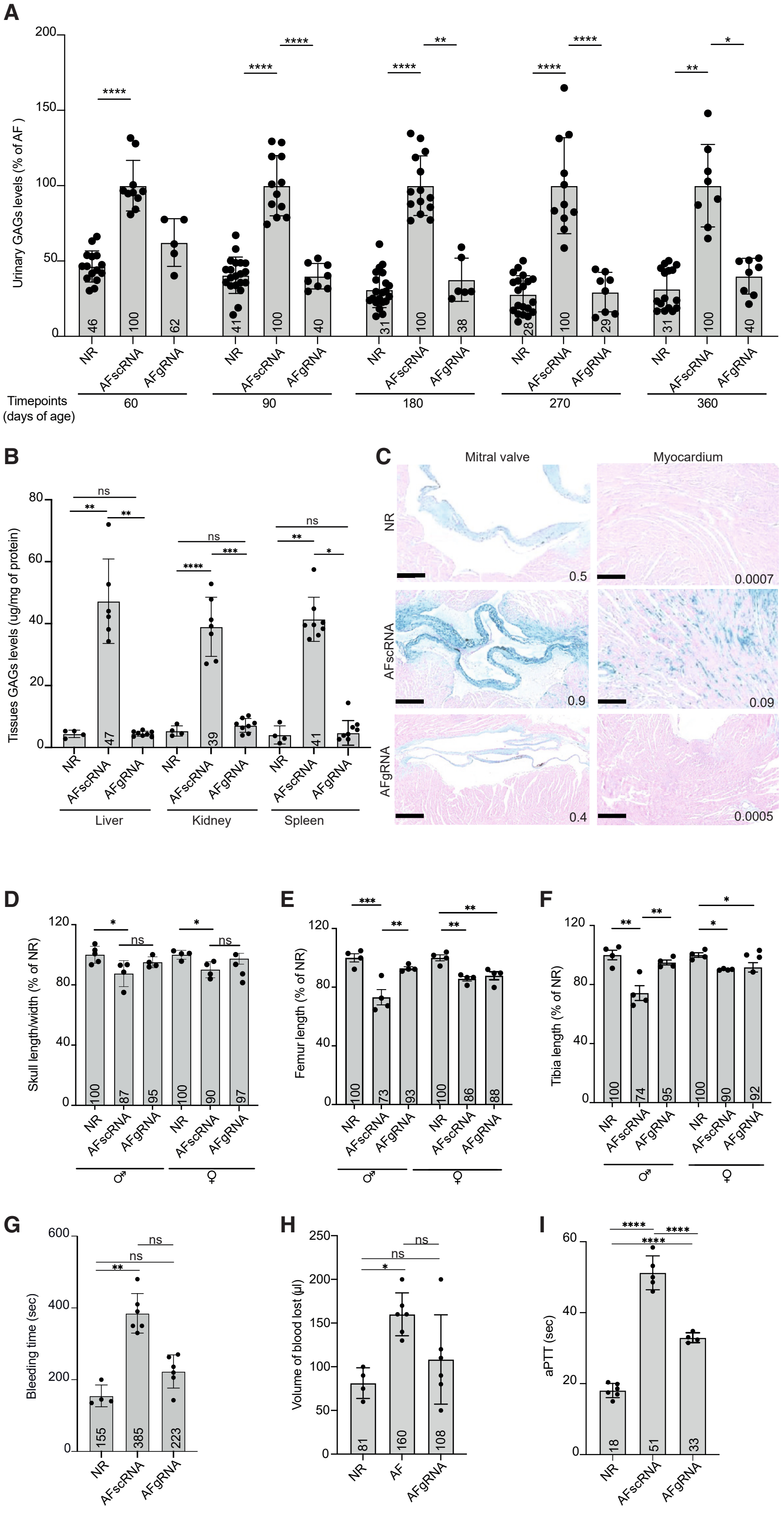

Figure 3. Liver-directed AAV-HITI therapeutic efficacy in newborn mice

(A) Urinary levels of glycosaminoglycans (GAGs) reported as a percentage of GAG levels in affected MPS VI mice (% of AF). Statistical differences were assessed by Kruskal-Wallis test and Dunn's multiple comparisons test. *p* values: *$p = 0.0379$, **$p = 0.0022$, and ****$p < 0.0001$. AFgRNA ($N = 8$), MPS VI mice treated with AAV-HITI-gRNA; AFscRNA ($N = 14$ and only $N = 8$ survived up to P360), MPS VI mice treated with AAV-HITI-scRNA. Each dot corresponds to a single animal within each group at different time points.

(B) Quantification of GAGs in the liver, kidney, and spleen. Statistical differences were assessed by Brown-Forsythe and Welch ANOVA tests. For the liver: **$p = 0.0016$ between NR ($N = 4$) and AFscRNA ($N = 6$); **$p = 0.0016$ between AFscRNA and AFgRNA ($N = 8$); $p = 0.9996$ between NR and AFgRNA. For the kidney: ****$p = 0.0001$ between NR ($N = 4$) and AFscRNA ($N = 7$); ***$p = 0.0002$ between AFscRNA and AFgRNA ($N = 8$); $p = 0.3784$ between NR and AFgRNA. For the spleen the Kruskal-Wallis test was used: **$p = 0.0029$ between NR ($N = 4$) and AFscRNA ($N = 8$); *$p = 0.0113$ between AFscRNA and AFgRNA ($N = 8$); $p > 0.9999$ between NR and AFgRNA. (A and B) Each dot corresponds to a single animal within each group at different time points. The differences in the number of analyzed samples within the same group of treatment at different time points were due to sample availability.

(C) Representative histological images of GAG storage in mitral heart valve and myocardium. Scale bar, 100 µm. Alcian blue quantification is reported inside the images as Alcian blue-positive area/total area. NR, $N = 4$; AFscRNA, $N = 8$; AFgRNA, $N = 8$.

(D–F) Measurement of skull length/width ratio, femur, and tibia lengths; data are reported as the percentage of normal length (% of NR). Males and females were kept separate in the analysis. AFgRNA, $N = 8$; AFscRNA, $N = 8$. Statistical differences were assessed by ordinary one-way ANOVA and Tukey's multiple comparisons test. (D) *p* value * = 0.0336 between NR and AFscRNA males; $p = 0.4847$ between NR and AFgRNA males; *$p = 0.0191$ between NR and AFscRNA females; $p = 0.6887$ between NR and AFgRNA females. (E) ***$p = 0.0011$ between NR and AFscRNA males; $p = 0.4491$ between NR and AFgRNA males; **$p = 0.0099$ between AFscRNA and AFgRNA males; **$p = 0.0122$ between NR and AFscRNA females; **$p = 0.0055$ between NR and AFgRNA females. (F) **$p = 0.0019$ between NR and AFscRNA males; **$p = 0.0089$ between NR and AFgRNA males. *$p = 0.0238$ between NR and AFscRNA females; *$p = 0.0398$ between NR and AFgRNA females.

(G and H) Tail-clip assay performed at 63 days of age in HemA AAV-HITI-treated male mice (AFgRNA, $N = 6$; AFscRNA, $N = 6$) and unaffected (NR; $N = 4$) controls. (G) **$p = 0.0019$; (H) *$p = 0.0129$.

(I) Activated partial thromboplastin time (aPTT) measured at 90 days of age in HemA AAV-HITI (AFgRNA, $N = 4$ and AFscRNA, $N = 5$) mice and unaffected (NR, $N = 6$) controls. *p* values: ****$p < 0.0001$ between NR and AFscRNA; ****$p < 0.0001$ between NR and AFgRNA; ****$p < 0.0001$ between AFscRNA and AFgRNA. All data are represented as mean ± standard deviation. See also Figure S3.

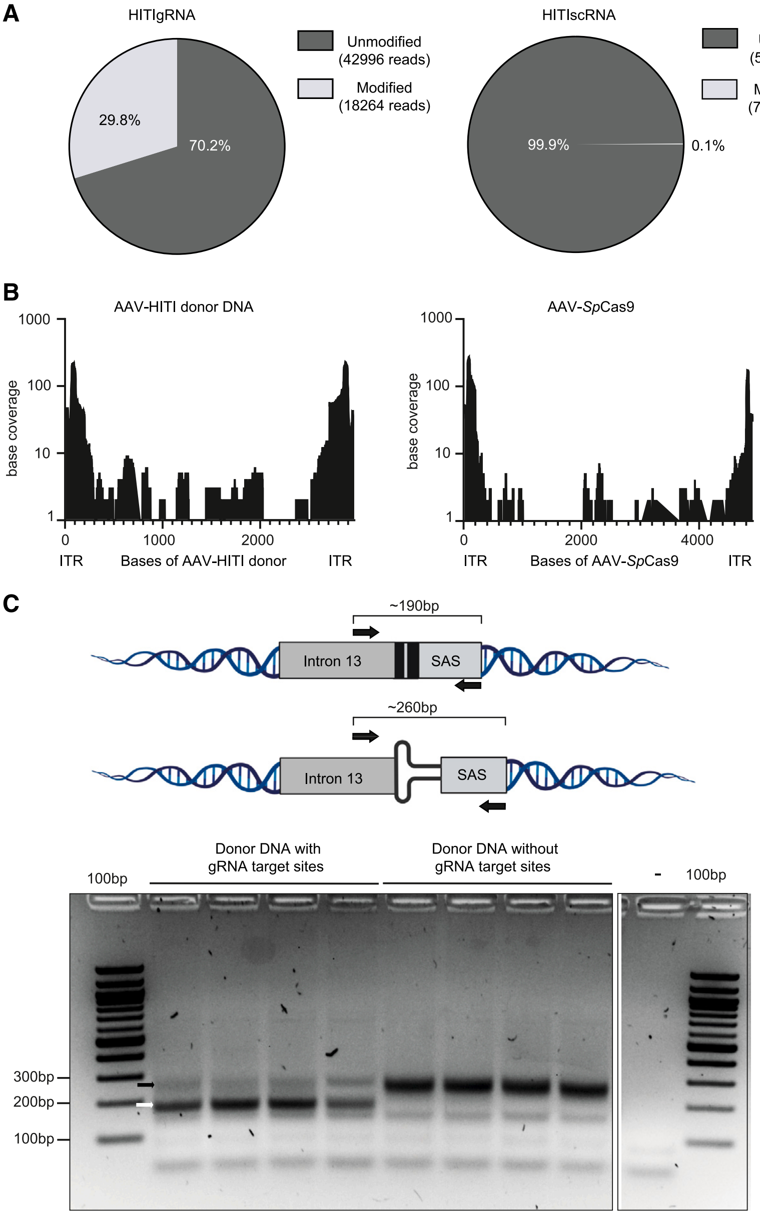

Figure 4. AAV-HITI molecular characterization at the on-target site

(A) Pie chart showing the modified reads observed in AAV-HITI (HITIgRNA or HITIscRNA)-treated mice analyzed by next-generation sequencing (NGS). The expected amplicon size is 285 bp, which was covered by 2x250 bp reads in NGS. The percentage of the modified reads is used as an indication of the gRNA efficiency at the on-target site (HITIgRNA~30% of indel).

(B) Sequences from different portions of both AAV vectors (AAV-HITI donor DNA and AAV-*Sp*Cas9) are captured at the induced double-strand breaks, mostly ITR sequences (ITR).

(C) Schematic representation of the strategy with the expected PCR amplicons and the position of the primers (up) and representative images of the 5′ junction PCR run on a 2% agarose gel (down). Four different biological samples for each group are showed in the gel. The white arrow indicates proper HITI-mediated donor DNA integration band (~190 bp); the black arrow indicates the ITR-mediated donor DNA integration band (~260 bp). See also Table S1.

gRNA target sites; Table S1). This donor should be integrated exclusively with its ITRs. Next, wild-type newborn C57BL/6 mice were randomly assigned to two different treatment groups and injected systemically by temporal vein at P1–2 at a total dose of 3.9 × 10^{13} GCs/kg. The same AAV vector encoding for the nuclease (AAV-*Sp*Cas9) was used in both groups and co-delivered with a second AAV carrying either the previously described (Figure 1B) dsRed HITI donor DNA (HITI donor) or the newly generated donor DNA without gRNA target sites. One-month post-treatment, genomic DNA was extracted from liver samples for molecular analysis. The 5′ junction was PCR amplified with specific primers (Figure 4C; Table S2) on DNA samples obtained from all the treated mice. A PCR product corresponding to the expected size (~260 bp) of the ITR-mediated integration was observed in samples treated with the donor DNA without gRNA target sites and the presence of ITRs confirmed by Sanger sequencing. Two junction PCR products of different sizes (~260 and ~190 bp; Figure 4C) were found in the samples from the AAV-HITI group. Sanger sequencing analysis showed that the smaller and more abundant PCR product (~190 bp) corresponded to the expected HITI-mediated integration while the higher (~260 bp) and less represented PCR product to the result of ITR-mediated integration.

To further evaluate the role of AAV ITRs in mediating full-length HITI donor DNA integration, we analyzed DNA samples from AFgRNA-treated MPS VI mice (previously injected to assess AAV-HITI efficacy) by long-read (LR) sequencing. We initially tried to amplify the target site upon HITI integration, hoping to detect the full-length integrated donor DNA. However, we obtained only small PCR products resembling the target site without HITI donor integration. This is very likely due to the strong bias toward amplification of smaller fragments during PCR. Here, the fragment derived from wild-type alleles is ~10-fold smaller than the expected fragment after HITI. In order to circumvent this PCR bias yet visualize the target site after successful HITI, we PCR-amplified in separate reactions two different long PCR products (5′ and 3′junctions; Figure 5A) whose size corresponded to the full-length donor DNA integrated at the on-target (~2 kb; Figure 5B). These long PCR fragments were analyzed by LR sequencing using Nanopore technology with specific primers (Table S3). We found that the majority of the obtained reads corresponded to the expected HITI-mediated integration; however, a small

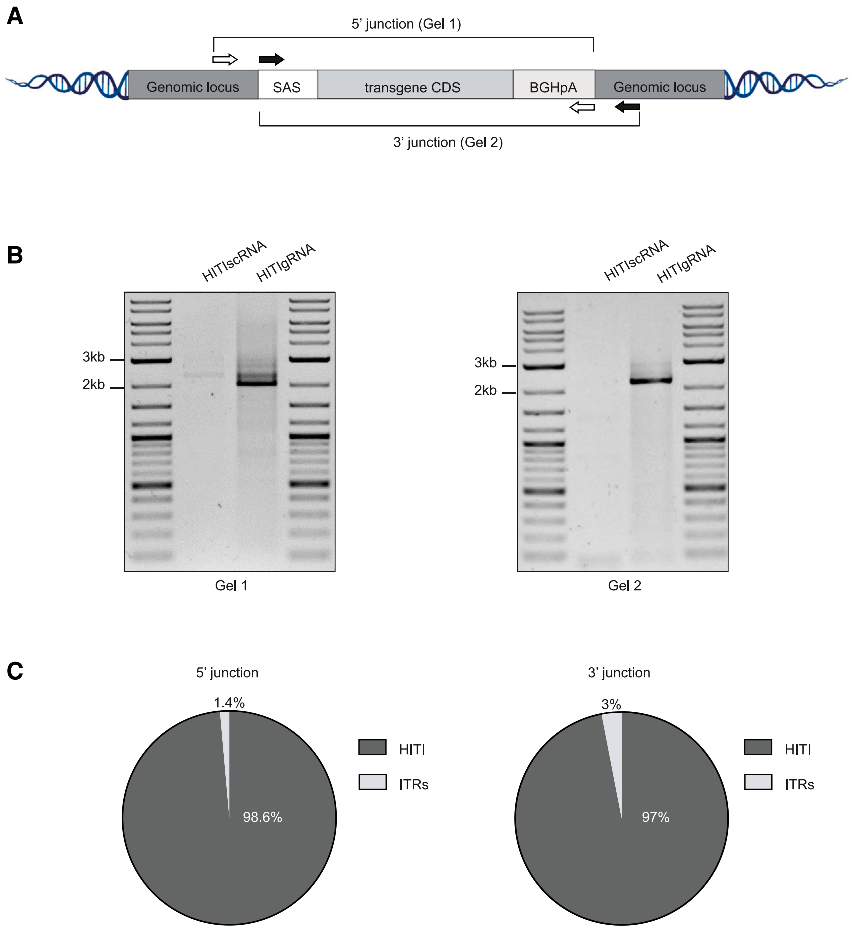

Figure 5. Long-reads analysis shows that full-length HITI donor DNA is integrated predominantly after ITR cleavage

(A) Schematic of the different long PCR junction products. White arrows indicate the primers designed to amplify the 5′ junction: the forward primer (white left) was designed in the endogenous locus right before the cleavage site; the reverse primer (white right) was designed at the end of the donor DNA on the polyadenylation signal (BGHpA). Black arrows indicate the primers designed to amplify the 3′junction: the forward primer (black left) was designed at the beginning of the donor DNA on the splicing acceptor signal (SAS); the reverse primer (black right) was designed in the endogenous locus right after the cleavage site.

(B) Representative images of the 5′ and 3′ junction PCR products run on an agarose gel for the detection of the full-length HITI donor DNA (~2 kb). The 5′ junction is showed in Gel 1 and the 3′ junction in Gel 2.

(C) Pie chart showing the percentage (%) of long reads in which donor DNA integration was HITI mediated or ITRs mediated (ITRs) at the junction sites. See also Figures S4, S6, and Tables S2 and S3.

fraction of reads presented with remnants of ITRs. We then tailored a bioinformatics pipeline to count the reads that contained ITR sequences, and we found that 1.4% of the 5′ junction reads and 3% of the 3′ junction reads contained substantial (>9 bp) parts of the ITR sequences (Figure 5C). To assess the frequency of the ITR-mediated donor DNA integration by a method complementary to LR sequencing, we performed Illumina-based short-read sequencing on samples treated with AAV-HITI. Short fragments (~200 bp) covering the 5′ junction were produced, and the number of reads containing (partial) ITRs was counted. Across three samples, we found that on average 9.8% (range 8.3%–11.3%) of the reads contained substantial (>9 bp) parts of an ITR. In line with the LR sequencing data, the vast majority of integrated fragments seem to have been released from the viral sequences prior to integration via HITI (Figure S4). To assess the various HITI donor DNA integration outcomes, we performed chromosomal aberrations analysis by single targeted linker-mediated PCR sequencing (CAST-Seq)[54] analysis (Figure S5) of liver DNA samples. CAST-Seq revealed six predominant integration events (Figure S6A): (1) the correct HITI event in the desired orientation (peak 1), (2) a fusion of intron 13 with the pA-encoding region of the AAV vector (peak 2), and (3) ITR-containing AAV fragments in both forward and reverse orientations (peaks 3–6; Figure S6A). Based on read coverage, we estimate that the intended HITI fusion product accounts for about 20% of integration events which is slightly higher than the integration in the reverse orientation (14.5%). The most frequent integration event is capturing of ITR-containing AAV fragments, maybe because they are shorter than the transgene-containing DNA sections (Figures S6A and S6B). In Figure S6C, only a sub-fraction of CAST-Seq reads was analyzed, i.e., only reads representing integration of cleaved (ITR-free) HITI donor template. This analysis includes peak 1 (19.7% of all integration events) and peak 2 (14.5% of all integration events) of Figures S6A and S6B but none of the other peaks (peaks 3–6). Of the total 34.2% (i.e., 19.7% + 14.5%) ITR-free HITI donor integration events, 48% are productive (Figure S6C). These 16.4% (48% of 34.2%) align very well with the 16% dsRed-positive hepatocytes observed by fluorescence microscopy (Figure 2A). Furthermore, the 34.2% of HITI integration events converge with the 49% HITI-positive nuclei observed in Figure S1C.

Safety of AAV-HITI following neonatal delivery

To assess the safety of AAV-HITI following neonatal treatment, we evaluated over time the amount of AAV-*Sp*Cas9 genomes following delivery in newborn mice and found a significant drop of about 30-fold after a month (Figure S7A), which is in line with data in Figure S1B.

To further investigate AAV-*Sp*Cas9 genome integration at the target site, we used CAST-Seq. We observed predominantly ITR-mediated capture of the AAV-*Sp*Cas9 genome without preference for either ITR (Figure S7B); however, Cas9 integration events were found to be less abundant (peak at 60,000 reads

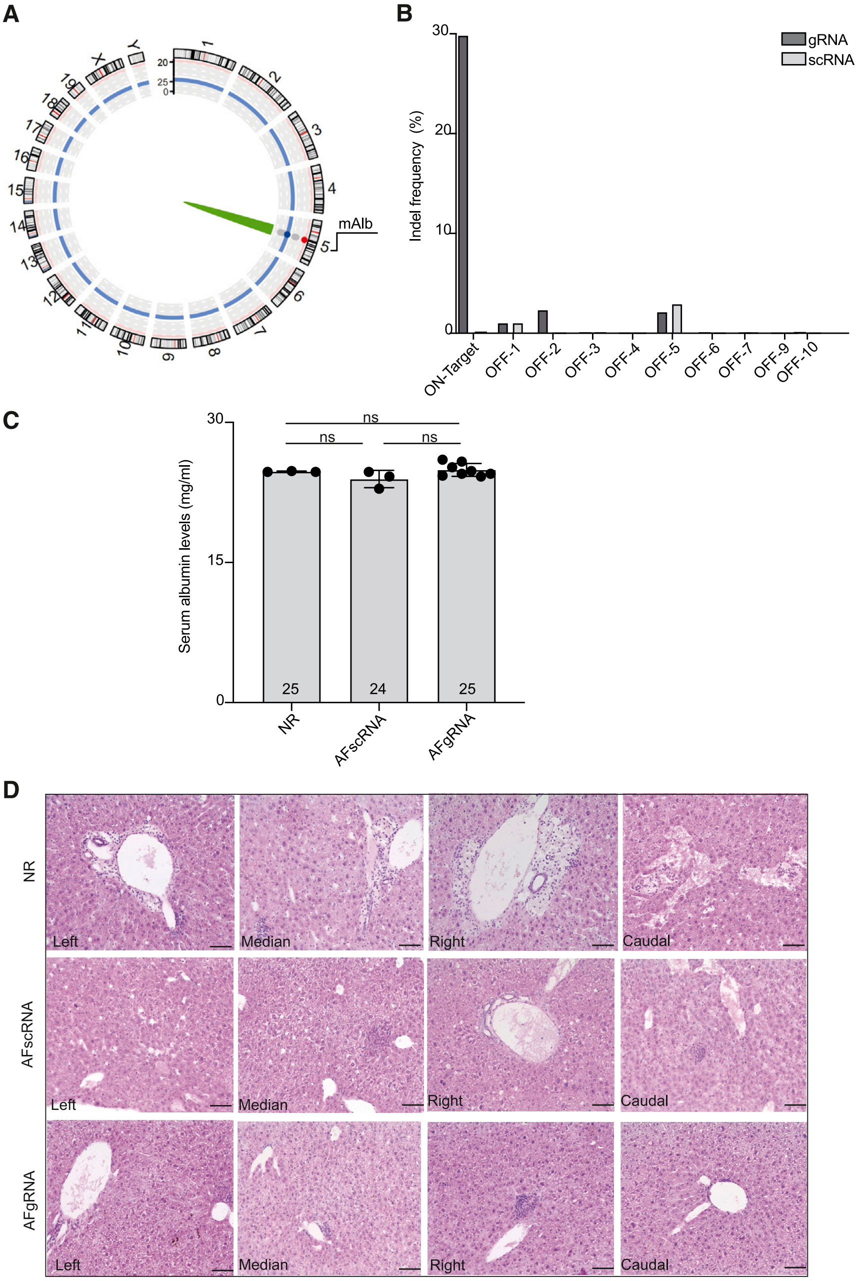

Figure 6. Safety following neonatal delivery of high doses of AAV-HITI

(A) The circos plot summarize the CAST-Seq analysis performed on genomic DNA extracted from liver samples of AAV-HITI-treated MPS VI mice. The on-target is reported in green. No chromosomal aberrations were found.

(B) Off-target activity (indel frequency) measured at the top predicted off-target loci by next-generation sequencing (NGS) analysis on genomic DNA extracted from liver samples of AAV-HITI-treated MPS VI. The off-target 8 (OFF-8) was excluded due to technical issues.

(C) Serum albumin levels measured 1-year post-treatment in sera samples from normal controls (NR, *N* = 3) or AAV-HITI-treated MPS VI mice (AFgRNA, *N* = 8; AFscRNA, *N* = 3); data are represented as mean ± standard deviation. Statistical differences were assessed by ordinary one-way ANOVA test.

(D) Representative images from histopathological analysis performed on paraffin sections from different liver lobes from AAV-HITI-treated MPS VI mice (AFgRNA, *N* = 8; AFscRNA, *N* = 8) and untreated normal controls (NR, *N* = 4) 1-year post-treatment. Scale bar, 50 μm. See also Figures S7, S8 and Tables S5–S7.

in Figure S7B) than HITI donor DNA integration events (peak at 200,000 reads in Figure S6A). Detection of internal AAV regions, in addition to the ITRs, suggests that internal regions were likely captured upon end resection of the vector genome (Figure S7B). To qualitatively assess potential full-length AAV-*Sp*Cas9 integration at the on-target site, we used specific primers as depicted in Figure S7C (Table S4). A faint PCR fragment corresponding to the expected size (~4.5 kb) was amplified in the AFgRNA- but not in the AFscRNA-treated sample (Figure S7C). Full-length *Sp*Cas9 integration was confirmed by Sanger sequencing analysis. To understand whether this could result in *Sp*Cas9 expression, we performed western blot analysis on AAV-HITI-treated liver lysates 1 year after vector delivery, and no Cas9 expression was detected (Figure S7D) suggesting that the risk of unwanted genetic editing due to long-term *Sp*Cas9 expression appears low under these conditions.

To evaluate potential CRISPR-Cas9-induced chromosomal rearrangement in an unbiased fashion, we performed CAST-Seq designed to this aim. We have shown before that CAST-Seq allows for the nomination of off-target (OT) sites through detection of OT-cleavage-induced translocations.[54] It furthermore enables assessment of DNA aberrations at the target site post nuclease treatment, including large deletions and inversions. CAST-Seq performed on genomic DNA extracted from liver samples of 3 different MPS VI mice treated with AAV-HITI (gRNA or scRNA; Table S5) revealed an exquisite safety profile of our nuclease. We did not detect any OT-mediated translocations (OMTs; Figure 6A) but only large deletions/inversions surrounding the on-target site (Figure S8A), in extreme cases reaching up to 35 kb. CAST-Seq also allowed us to assess the relative frequencies of gross chromosomal aberrations at the target site. We found that 2.4% of CAST-Seq reads indicate deletions and inversions larger than 200 bp, whereas only 0.6% of reads were detected in AAV-HITI scRNA-treated mice (Figure S8B).

Moreover, we used *in silico* prediction to identify putative OTs and selected the top 10 candidates for downstream analysis (Table S6). We PCR-amplified the region surrounding the

expected OT cleavage site with primers specific for each locus (Table S7). PCR products were analyzed by NGS for the presence of indel mutations. Indels were found to be almost undetectable or present at similar frequency in AFgRNA- and AFscRNA-treated samples (Figure 6B), underlining that the employed CRISPR-Cas9 nuclease is highly specific. Furthermore, we measured serum albumin levels in AAV-HITI-treated (gRNA or scRNA) MPS VI mice and unaffected littermates at P360 and confirmed that our AAV-HITI approach does not alter endogenous albumin expression (Figure 6C). Histopathological analysis performed on liver sections from all AAV-HITI-treated (AFgRNA or AFscRNA) mice and unaffected controls showed no evidence of hepatocellular carcinoma (HCC) at this stage (P360; Figure 6D).

Dose response of liver-directed AAV-HITI in newborn mice

We next performed a dose-response study with two additional doses of AAV-HITI: a medium dose (MD) of 3.9 × 10^{13} total GCs/kg and a low dose (LD) of 1.2 × 10^{13} total GCs/kg. AAV-HITI efficiency at these new doses was assessed in newborn C57BL/6 mice using the dsRed HITI donor DNA. Animals were randomly assigned to the MD or LD group and injected systemically at P1–2, as described earlier. The percentage of hepatocytes expressing dsRed as a result of the integration (used to evaluate AAV-HITI efficiency) was quantified on liver cryo-sections 1-month post-treatment and found to be AAV dose dependent (Figure S9A). Next, the efficacy of AAV-HITI at these same doses (MD and LD) was evaluated in newborn MPS VI mice. A similar dose-dependent effect on serum ARSB activity was observed. Mice administered with AAV-HITI at MD exhibited sustained and stable serum ARSB activity levels starting from 1-month post-treatment, while mice treated with AVV-HITI at LD stably achieved ~50% of the normal ARSB levels (Figure S9B). Urinary (Figure S9C) and tissue GAG levels were normalized in AFgRNA-treated MPS VI mice (Figures S10A–S10D). GAGs storage was significantly decreased also in histological sections from the heart mitral valve and myocardium (Figure S10E) regardless of the dose used. In MD AFgRNA-treated mice, we also observed a significant amelioration in the skull width/length ratio and in the femur length compared to AF controls (Figures S10F–S10H).

Liver-directed AAV-HITI is effective in adult mice

To understand the applicability and efficiency of AAV-HITI in the adult liver, MPS VI and HemA mice were randomly administered systemically at 6 weeks of age an LD of 1.2 × 10^{13} GCs/kg of AAV-HITI vectors. For MPS VI mice detectable serum ARSB activity was measured in the AFgRNA-treated animals at 90 days of age, and this activity became supraphysiological at the subsequent time points (Figure 7A). Moreover, AFgRNA-treated mice showed decreased levels of urinary (Figure 7B) and tissue GAGs compared to the AF control mice (Figures S11A–S11D). GAGs storage was also reduced in histological sections from the heart mitral valve and myocardium (Figure S11E) while no significant improvement was observed in the analyzed bones (Figures S11F–S11H). In HemA mice at 30 days post AAV-HITI delivery, we observed FVIII protein levels around 36% of normal (Figure 7C), therapeutic levels of FVIII activity (Figure 7D), and reduced clotting time (Figure 7E). These data indicate that AAV-HITI is effective in the adult liver at moderate AAV doses in addition to newborn.

DISCUSSION

In this study, we have demonstrated the therapeutic efficacy of liver-directed AAV-HITI at the highly transcribed m*Alb* locus both in the newborn and in the adult liver. Several previous studies have reported targeting of the albumin locus[30,37,55] with overall similar levels of transgene integration to those we obtained here. HITI, however, hijacks NHEJ able to target both newborn and adult tissues[21,22,25,34] without the need for long homology arms in the donor template. This allows to accommodate larger therapeutic sequences. Importantly, here we show that the majority of the full-length donor DNA integration happened after CRISPR-Cas9 cleavage of the ITRs, as intended by its design, which is in line with the lower integration rate of the AAV-*Sp*Cas9 vector which lacks the inverted gRNA sites. This, combined with the slightly favored desired integration of the donor DNA that we observed using CAST-Seq, highlights the importance of the inverted gRNA sites at both extremities of the donor DNA. In addition, we found that integration of internal AAV-HITI donor DNA sequences was rare, suggesting that excessive end resection upon CRISPR-Cas-mediated cleavage of the HITI donor DNA does not occur.

Furthermore, we demonstrated the safety of AAV-HITI following neonatal delivery at high doses. AAV-HITI combines two different technologies whose safety is under evaluation: AAV vectors and CRISPR-Cas9 nuclease. AAV vectors are predominantly non-integrative; however, it has been shown that portions of their genome can integrate into pre-existing DSBs at a low frequency.[56] Despite this low level of integrations, a number of studies have been conducted in mice to investigate potential genotoxicity associated with these events particularly after neonatal treatment.[57,58] Data collected in animal studies suggest that several factors such as age of treatments, vector doses, and vector design may influence genotoxic events related to AAV vectors.[57] However, no confirmed genotoxicity associated with AAV therapies has been described in humans to date.[57] In our recent publication,[21] we performed genome-wide analysis of liver genomic DNA following AAV neonatal delivery and found partial AAV integrations at spontaneous DSBs. Here, we showed that portions of the AAV vector genomes can be found at the CRISPR-Cas9-induced DSBs with most of these integrations falling within the AAV ITR sequences, in line with previously reported data.[59–61] To further investigate the safety of our approach, we thoroughly assessed AAV-*Sp*Cas9 integration and long-term expression and found that, albeit detectable, full-length integration occurs at low levels and does not result in long-term detectable protein expression in the transduced liver.

Moreover, when nuclease-based strategies are proposed for therapeutic applications, the need to assess nuclease specificity and genome integrity is crucial to reduce the risk of genotoxicity.[54] Indeed, poor nuclease specificity may result in indel mutations at OT sites and large chromosomal aberrations.[54] Notably, the CAST-Seq data combined with the assessment of *in silico*-predicted OT sites showed that, despite low rates of partial

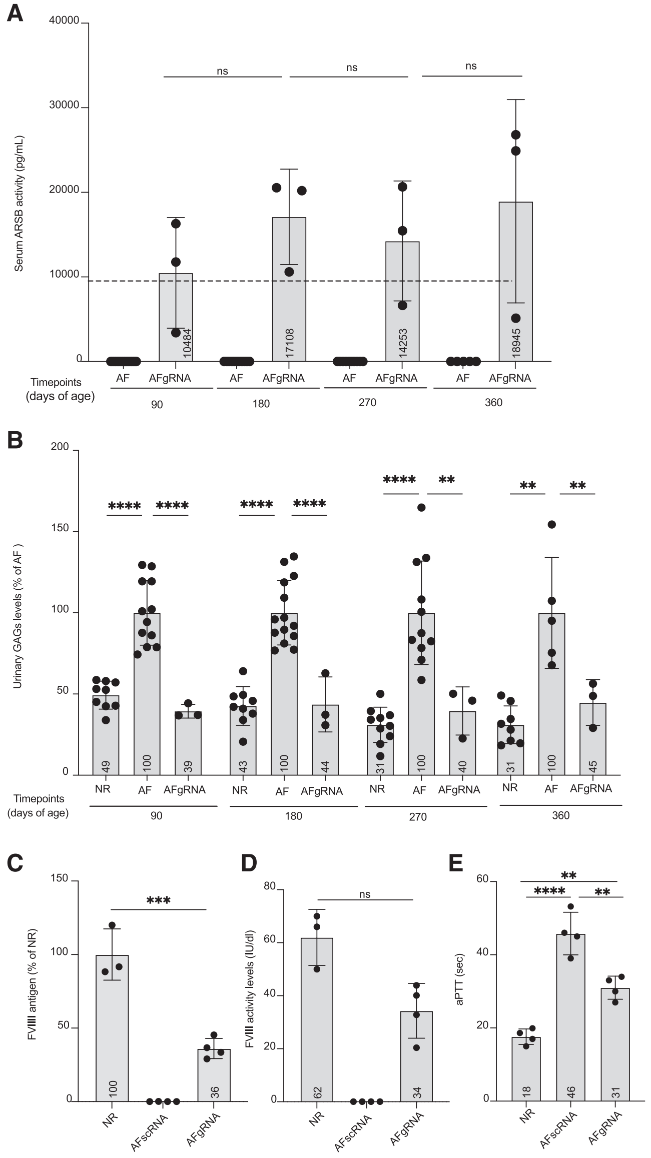

Figure 7. Liver-directed AAV-HITI in adult mice

(A) Serum ARSB activity analyzed at different time points in MPS VI mice left untreated (AF, $N = 14$) or treated with AAV-HITI-gRNA (AFgRNA, $N = 3$) at the total dose of 1.2×10^{13} total GCs/kg.

(B) Urinary GAG levels at different time points in MPS VI mice left untreated (AF, $N = 14$) or treated with AAV-HITI-gRNA (AFgRNA, $N = 3$) reported as a percentage of GAG levels in untreated MPS VI mice. NR, sera samples from normal animals. Statistical differences were assessed at P90 by Brown-Forsythe and Welch ANOVA tests: ****$p < 0.0001$ between NR and AF; ****$p < 0.0001$ between AF and AFgRNA; by Kruskal-Wallis test at P180: ****$p < 0.0001$ between NR and AF; ****$p < 0.0001$ between AF and AFgRNA; by ordinary one-way ANOVA test at P270: ****$p < 0.0001$ between NR and AF; **$p < 0.0007$ between AF and AFgRNA; at P360: **$p = 0.0012$ between NR and AF; $p = 0.9999$ between NR and AFgRNA; **$p = 0.0078$ between AF and AFgRNA. The differences in the number of analyzed samples within the same group of treatment were due to sample availability. Each dot corresponds to a single animal within each group at different time points. (A and B) The differences in the number of analyzed samples within the same group of treatment at different time points were due to sample availability.

(C) FVIII antigen levels detected in AAV-HITI (AFscRNA, $N = 4$; AFgRNA, $N = 4$)-treated adult HemA mice and normal controls (NR, $N = 3$) 1-month post AAV-HITI treatment. ***$p = 0.0001$ between NR and AFgRNA.

(D) FVIII activity levels evaluated in HemA mice (AFgRNA, $N = 4$; AFscRNA, $N = 4$) and normal controls (NR, $N = 3$) by chromogenic assay 1-month post AAV-HITI treatment. Statistical differences were assessed by ordinary one-way ANOVA test. p value between NR and AFgRNA = 0.0783.

(E) Activated partial thromboplastin time (aPTT) measured 1 month post AAV-HITI treatment in HemA AAV-HITI (AFgRNA, $N = 4$ and AFscRNA, $N = 4$) mice and normal controls (NR; $N = 4$). ****$p < 0.0001$ between NR and AFscRNA; **$p = 0.0028$ between NR and AFgRNA; **$p = 0.0014$ between AFscRNA and AFgRNA. All data are represented as mean ± standard deviation. See also Figure S11.

AAV integrations at the on-target site, neither intra-chromosomal rearrangements nor OT indels could be detected. CAST-Seq further revealed that a minor portion of reads (2.4%) from the target locus pointed at large chromosomal aberrations. Despite their low frequency, these events warrant consideration for potential safety implications. Of note, these data are in stark contrast to the high frequency (often exceeding 50%) at which such reads are found in cells that were exposed to a CRISPR-Cas9 nuclease in the absence of a donor DNA template (Klermund et al., in revision; Rhiel et al., in preparation), suggesting that the presence of an AAV donor DNA template could mitigate these chromosomal aberrations. However, more experiments, and in particular proper side-by-side comparisons, are needed to confirm this hypothesis.

Additionally, the absence of HCC up to 1 year after neonatal treatment supports the safety of the AAV-HITI platform. Of note, our AAV-HITI approach is based on the use of a weak HLP to drive *Sp*Cas9 expression and no promoter in the donor DNA. This design should limit the risk of genotoxicity, as also demonstrated by Chandler et al.[62] Nonetheless, to exclude potential AAV-HITI-induced genotoxicity associated with HCC development in mice, longer follow-up up to 18–24 months of age will be necessary.[63]

The unaltered secreted albumin levels support the safe design of HITI in albumin intron 13, as correct HITI insertions are not predicted to affect albumin gene expression. However, we should not underestimate the risk that undesired integration events may introduce potential cryptic acceptor splice sites that could alter expression from the endogenous locus. While this does not affect albumin levels in our case, it should not be overlooked, especially if HITI is performed in other genes.

Since high doses of AAVs may lead to adverse events,[64,65] we performed a dose-response study in which we found that AAV-HITI administered in neonatal mice at 1.2×10^{13} total GCs/kg resulted in ∼50% of the normal levels of serum ARSB activity, which significantly improved several features of the mouse disease phenotype. If directly translatable to humans, this dose is well tolerated. We also tested this same AAV-HITI dose in adult MPS VI and HemA mice and found it to be highly effective thus expanding the potential applications of AAV-HITI to conditions that require intervention in the adult liver.

Overall, our data support the use of the liver-directed AAV-HITI for sustained and stable expression of therapeutic transgenes to treat inborn genetic disorders. However, additional studies are required to further assess its translational potential. For instance,

CellPress
OPEN ACCESS

unwanted AAV genome integration at the on-target site could be mitigated using low doses of AAV-HITI,[25] or by using non-viral systems such as lipid nanoparticles (LNPs) for nuclease delivery which provide transient expression.[66]

Finally, validating gRNA sequences tailored for the human albumin locus and assessing the effectiveness and safety of AAV-HITI in mice with humanized liver could represent a significant step toward advancing this approach for clinical application.

Limitations of the study

Future implementations of this study could include longer animal follow-up and formal Investigational New Drug (IND)-enabling studies to further support the safety of the AAV-HITI platform and unbiased LR whole-genome sequencing to further strengthen the characterization of HITI-mediated editing events.

STAR★METHODS

Detailed methods are provided in the online version of this paper and include the following:

- KEY RESOURCES TABLE
- RESOURCE AVAILABILITY
  - Lead contact
  - Materials availability
  - Data and code availability
- EXPERIMENTAL MODEL AND SUBJECT DETAILS
  - Animal models
- METHODS DETAILS
  - Study design
  - Systemic vector administration
  - Generation of the AAV vector plasmids
  - AAV vector production and characterization
  - Liver fluorescence imaging
  - *In situ* hybridization
  - Serum ARSB enzymatic activity
  - Chromogenic assay
  - FVIII antigen detection
  - GAG level analysis in urine and tissues
  - Alcian blue staining in histological sections
  - Bone analysis
  - Tail-clip assay
  - Activated partial thromboplastin time (aPTT) assay
  - Western blot analysis
  - Serum albumin measurement
  - Histopathological analysis
  - DNA extraction
  - Quantitative PCR (qPCR)
  - CAST-Seq
  - Nanopore long-read sequencing
  - Short-read sequencing
  - Off-target site analysis
- QUANTIFICATION AND STATISTICAL ANALYSIS

SUPPLEMENTAL INFORMATION

Supplemental information can be found online at https://doi.org/10.1016/j.xcrm.2024.101619.

ACKNOWLEDGMENTS

We thank TIGEM Advanced Histopathology Facility for tissue processing and embedding of liver, spleen, kidney, and heart tissues; imaging; quantification of Alcian blue staining in heart tissue; and for DNAscope experiments. We thank Cogentech s.r.l. for the liver histopathology analysis; NEGEDIA s.r.l. for the off-target analysis; InnovaVector s.r.l. for AAV vectors preparation; TIGEM Bioinformatics Core, particularly Eugenio Del Prete, for the statistical analysis; and Biorender.com for the cartoons used. We gratefully acknowledge Dr. Cathal Wilson (TIGEM) for editing of the text. This work was supported by the European Union Horizon 2020 grant "UPGRADE" (grant 825825), the Italian UniNA and "Compagnia di San Paolo" Star Plus 2020 Established Principal Investigator (grant 21-UNINA-EPIG-007), the European Union's EIC Pathfinder program AAVolution (grant 101071041), and the ERC EXPEDITE grant (grant 101097155). We also acknowledge funding from the European Union, project geneTIGA grant-101057438 toT.C., and the German Federal Ministry of Education and Research (BMBF) within the Medical Informatics Funding Scheme: MIRACUM–FKZ 01ZZ1801B and PM^{4}Onco–FKZ 01ZZ2322A (M.B.), and EkoEstMed–FKZ 01ZZ2015 (G.A.).

AUTHOR CONTRIBUTIONS

F.E. and A.A. conceived the study and wrote the manuscript. F.E., P.S.H., S.A., and M.L.S. generated plasmids for AAV production. F.E., F.D.A., R.F., and M.D.A. performed the experiments in MPS VI mice. F.E., P.S.H., and S.A. performed the experiments in C57BL/6 mice. F.E. and S.A. performed the experiments in HemA mice. F.E., F.D.A., E.N., S.A., R.D.C., and M.D.A. performed *in vivo* procedures. A. Padmanabhan and S. Notaro processed the different MPS VI tissues. A. Padula and F.E. designed and performed the western blot analysis on sera samples. F.E. and S.A. performed the western blot analysis on liver samples. P.P. and I.T. contributed to the interpretation of the results. F.E. performed the off-target NGS experiments and analyzed the data. M.R., K.O.C., G.A., M.B., T.I.C., and T.C. designed, performed, and analyzed CAST-Seq, on-target NGS, and long-read sequencing experiments. F.E. designed and performed the short-read experiments. M.R. and K.O.C. analyzed the short-read sequencing data. N.C.S. and S. Nutarelli developed customized ISH protocols and performed and analyzed the ISH experiments.

DECLARATION OF INTERESTS

F.E., F.D.A., R.F., M.L.S., and A.A. are listed as inventors on the patent WO2023213831 "Homology independent targeted integration for gene editing" related to this work. A.A. is founder, shareholder, and consultant of InnovaVector s.r.l. and of AAVantgarde Bio s.r.l. R.F. is currently an employee of AAVantgarde Bio s.r.l. T.C., M.B., and G.A. are listed as inventors of CAST-Seq (EP3856928B1).

Received: October 4, 2023
Revised: February 13, 2024
Accepted: May 27, 2024
Published: June 18, 2024

REFERENCES

1. Zabaleta, N., Unzu, C., Weber, N.D., and Gonzalez-Aseguinolaza, G. (2023). Gene therapy for liver diseases — progress and challenges. Nat. Rev. Gastroenterol. Hepatol. *20*, 288–305. https://doi.org/10.1038/s41575-022-00729-0.
2. Mendell, J.R., Al-Zaidy, S.A., Rodino-Klapac, L.R., Goodspeed, K., Gray, S.J., Kay, C.N., Boye, S.L., Boye, S.E., George, L.A., Salabarria, S., et al. (2021). Current Clinical Applications of In Vivo Gene Therapy with AAVs. Mol. Ther. *29*, 464–488. https://doi.org/10.1016/j.ymthe.2020.12.007.
3. Au, H.K.E., Isalan, M., and Mielcarek, M. (2021). Gene Therapy Advances: A Meta-Analysis of AAV Usage in Clinical Settings. Front. Med. *8*, 809118–809214. https://doi.org/10.3389/fmed.2021.809118.
4. Jeyakumar, J.M., Kia, A., Tam, L.C.S., McIntosh, J., Spiewak, J., Mills, K., Heywood, W., Chisari, E., Castaldo, N., Verhoef, D., et al. (2023). Preclinical evaluation of FLT190, a liver-directed AAV gene therapy for Fabry disease. Gene Ther. *30*, 487–502. https://doi.org/10.1038/s41434-022-00381-y.

5. Brunetti-Pierri, N., Ferla, R., Ginocchio, V.M., Rossi, A., Fecarotta, S., Romano, R., Parenti, G., Yildiz, Y., Zancan, S., Pecorella, V., et al. (2022). Liver-Directed Adeno-Associated Virus–Mediated Gene Therapy for Mucopolysaccharidosis Type VI. NEJM Evid. *1*, EVIDoa2200052-12. https://doi.org/10.1056/evidoa2200052.

6. Ginocchio, V.M., Ferla, R., Auricchio, A., and Brunetti-Pierri, N. (2019). Current Status on Clinical Development of Adeno-Associated Virus-Mediated Liver-Directed Gene Therapy for Inborn Errors of Metabolism. Hum. Gene Ther. *30*, 1204–1210. https://doi.org/10.1089/hum.2019.151.

7. Miesbach, W., Meijer, K., Coppens, M., Kampmann, P., Klamroth, R., Schutgens, R., Tangelder, M., Castaman, G., Schwäble, J., Bonig, H., et al. (2018). Gene therapy with adeno-associated virus vector 5–human factor IX in adults with hemophilia B. Blood *131*, 1022–1031. https://doi.org/10.1182/blood-2017-09-804419.

8. Nathwani, A.C., Tuddenham, E.G.D., Rangarajan, S., Rosales, C., McIntosh, J., Linch, D.C., Chowdary, P., Riddell, A., Pie, A.J., Harrington, C., et al. (2011). Adenovirus-Associated Virus Vector–Mediated Gene Transfer in Hemophilia B. N. Engl. J. Med. *365*, 2357–2365. https://doi.org/10.1056/nejmoa1108046.

9. Nathwani, A.C., Reiss, U.M., Tuddenham, E.G.D., Rosales, C., Chowdary, P., McIntosh, J., Peruta, M.D., Lheriteau, E., Patel, N., Raj, D., et al. (2015). Long term gene therapy for FiX. N. Engl. J. Med. *371*, 1994–2004. https://doi.org/10.1056/NEJMoa1407309.Long-Term.

10. Ehrhardt, A., Xu, H., and Kay, M.A. (2003). Episomal Persistence of Recombinant Adenoviral Vector Genomes during the Cell Cycle In Vivo. J. Virol. *77*, 7689–7695. https://doi.org/10.1128/jvi.77.13.7689-7695.2003.

11. Cotugno, G., Annunziata, P., Barone, M.V., Karali, M., Banfi, S., and Auricchio, A. (2012). Impact of age at administration, lysosomal storage, and transgene regulatory elements on AAV2/8-mediated rat liver transduction. PLoS One *7*, e33286. https://doi.org/10.1371/journal.pone.0033286.

12. Wang, L., Wang, H., Bell, P., McMenamin, D., and Wilson, J.M. (2012). Hepatic gene transfer in Neonatal mice by Adeno-associated virus serotype 8 vector. Hum. Gene Ther. *23*, 533–539. https://doi.org/10.1089/hum.2011.183.

13. Cunningham, S.C., Spinoulas, A., Carpenter, K.H., Wilcken, B., Kuchel, P.W., and Alexander, I.E. (2009). AAV2/8-mediated correction of OTC deficiency is robust in adult but not neonatal Spfash mice. Mol. Ther. *17*, 1340–1346. https://doi.org/10.1038/mt.2009.88.

14. Cunningham, S.C., Dane, A.P., Spinoulas, A., and Alexander, I.E. (2008). Gene delivery to the juvenile mouse liver using AAV2/8 vectors. Mol. Ther. *16*, 1081–1088. https://doi.org/10.1038/mt.2008.72.

15. Earley, J., Piletska, E., Ronzitti, G., and Piletsky, S. (2023). Evading and overcoming AAV neutralization in gene therapy. Trends Biotechnol. *41*, 836–845. https://doi.org/10.1016/j.tibtech.2022.11.006.

16. Hamilton, B.A., and Wright, J.F. (2021). Challenges Posed by Immune Responses to AAV Vectors: Addressing Root Causes. Front. Immunol. *12*, 675897–675898. https://doi.org/10.3389/fimmu.2021.675897.

17. Mingozzi, F., Maus, M.V., Hui, D.J., Sabatino, D.E., Murphy, S.L., Rasko, J.E.J., Ragni, M.V., Manno, C.S., Sommer, J., Jiang, H., et al. (2007). CD8(+) T-cell responses to adeno-associated virus capsid in humans. Nat. Med. *13*, 419–422. https://doi.org/10.1038/nm1549.

18. Manno, C.S., Pierce, G.F., Arruda, V.R., Glader, B., Ragni, M., Rasko, J.J., Ozelo, M.C., Hoots, K., Blatt, P., Konkle, B., et al. (2006). Successful transduction of liver in hemophilia by AAV-Factor IX and limitations imposed by the host immune response. Nat. Med. *12*, 342–347. https://doi.org/10.1038/nm1358.

19. Ertl, H.C.J. (2022). Immunogenicity and toxicity of AAV gene therapy. Front. Immunol. *13*, 975803–975809. https://doi.org/10.3389/fimmu.2022.975803.

20. Chavez, M., Chen, X., Finn, P.B., and Qi, L.S. (2023). Advances in CRISPR therapeutics. Nat. Rev. Nephrol. *19*, 9–22. https://doi.org/10.1038/s41581-022-00636-2.

21. Tornabene, P., Ferla, R., Llado-Santaeularia, M., Centrulo, M., Dell'Anno, M., Esposito, F., Marrocco, E., Pone, E., Minopoli, R., Iodice, C., et al. (2022). Therapeutic homology-independent targeted integration in retina and liver. Nat. Commun. *13*, 1963–2014. https://doi.org/10.1038/s41467-022-29550-8.

22. Wang, Q., Zhong, X., Li, Q., Su, J., Liu, Y., Mo, L., Deng, H., and Yang, Y. (2020). CRISPR-Cas9-Mediated In Vivo Gene Integration at the Albumin Locus Recovers Hemostasis in Neonatal and Adult Hemophilia B Mice. Mol. Ther. Methods Clin. Dev. *18*, 520–531. https://doi.org/10.1016/j.omtm.2020.06.025.

23. Li, T., Yang, Y., Qi, H., Cui, W., Zhang, L., Fu, X., He, X., Liu, M., Li, P.F., and Yu, T. (2023). CRISPR/Cas9 therapeutics: progress and prospects. Signal Transduct. Target. Ther. *8*, 36. https://doi.org/10.1038/s41392-023-01309-7.

24. Frangoul, H., Altshuler, D., Cappellini, M.D., Chen, Y.-S., Domm, J., Eustace, B.K., Foell, J., de la Fuente, J., Grupp, S., Handgretinger, R., et al. (2021). CRISPR-Cas9 Gene Editing for Sickle Cell Disease and β-Thalassemia. N. Engl. J. Med. *384*, 252–260. https://doi.org/10.1056/nejmoa2031054.

25. He, X., Zhang, Z., Xue, J., Wang, Y., Zhang, S., Wei, J., Zhang, C., Wang, J., Urip, B.A., Ngan, C.C., et al. (2022). Low-dose AAV-CRISPR-mediated liver-specific knock-in restored hemostasis in neonatal hemophilia B mice with subtle antibody response. Nat. Commun. *13*, 7275. https://doi.org/10.1038/s41467-022-34898-y.

26. Gillmore, J.D., Gane, E., Taubel, J., Kao, J., Fontana, M., Maitland, M.L., Seitzer, J., O'Connell, D., Walsh, K.R., Wood, K., et al. (2021). CRISPR-Cas9 In Vivo Gene Editing for Transthyretin Amyloidosis. N. Engl. J. Med. *385*, 493–502. https://doi.org/10.1056/nejmoa2107454.

27. Bart P. L., Koenekoop, R.K., Porto, F.B.O., Russell, S.R., and Girach, A. (2011). Leber Congenital Amaurosis Due to Cep290 Mutations — Severe Vision Impairment with a High Unmet Medical. 898–907.

28. Ou, L., Przybilla, M.J., Ahlat, O., Kim, S., Overn, P., Jarnes, J., O'Sullivan, M.G., and Whitley, C.B. (2020). A Highly Efficacious PS Gene Editing System Corrects Metabolic and Neurological Complications of Mucopolysaccharidosis Type I. Mol. Ther. *28*, 1442–1454. https://doi.org/10.1016/j.ymthe.2020.03.018.

29. Tao, J., Bauer, D.E., and Chiarle, R. (2023). Assessing and advancing the safety of CRISPR-Cas tools: from DNA to RNA editing. Nat. Commun. *14*, 212. https://doi.org/10.1038/s41467-023-35886-6.

30. De Caneva, A., Porro, F., Bortolussi, G., Sola, R., Lisjak, M., Barzel, A., Giacca, M., Kay, M.A., Vlahoviček, K., Zentilin, L., and Muro, A.F. (2019). Coupling AAV-mediated promoterless gene targeting to SaCas9 nuclease to efficiently correct liver metabolic diseases. JCI Insight *5*, e128863. https://doi.org/10.1172/jci.insight.128863.

31. Hong, S.A., Seo, J.H., Wi, S., Jung, E.S., Yu, J., Hwang, G.H., Yu, J.H., Baek, A., Park, S., Bae, S., and Cho, S.R. (2022). In vivo gene editing via homology-independent targeted integration for adrenoleukodystrophy treatment. Mol. Ther. *30*, 119–129. https://doi.org/10.1016/j.ymthe.2021.05.022.

32. Suzuki, K., and Izpisua Belmonte, J.C. (2018). In vivo genome editing via the HITI method as a tool for gene therapy. J. Hum. Genet. *63*, 157–164. https://doi.org/10.1038/s10038-017-0352-4.

33. Suzuki, K., Tsunekawa, Y., Hernandez-Benitez, R., Wu, J., Zhu, J., Kim, E.J., Hatanaka, F., Yamamoto, M., Araoka, T., Li, Z., et al. (2016). In vivo genome editing via CRISPR/Cas9 mediated homology-independent targeted integration. Nature *540*, 144–149. https://doi.org/10.1038/nature20565.

34. Chen, X., Niu, X., Liu, Y., Zheng, R., Yang, L., Lu, J., Yin, S., Wei, Y., Pan, J., Sayed, A., et al. (2022). Long-term correction of hemophilia B through CRISPR/Cas9 induced homology-independent targeted integration. J. Genet. Genomics *49*, 1114–1126. https://doi.org/10.1016/j.jgg.2022.06.001.

35. Chen, H., Shi, M., Gilam, A., Zheng, Q., Zhang, Y., Afrikanova, I., Li, J., Gluzman, Z., Jiang, R., Kong, L.J., and Chen-Tsai, R.Y. (2019). Hemophilia

CellPress
OPEN ACCESS

A ameliorated in mice by CRISPR-based in vivo genome editing of human Factor VIII. Sci. Rep. *9*, 16838–16915. https://doi.org/10.1038/s41598-019-53198-y.

36. Porro, F., Bortolussi, G., Barzel, A., De Caneva, A., Iaconcig, A., Vodret, S., Zentilin, L., Kay, M.A., and Muro, A.F. (2017). Promoterless gene targeting without nucleases rescues lethality of a Crigler-Najjar syndrome mouse model. EMBO Mol. Med. *9*, 1346–1355. https://doi.org/10.15252/emmm.201707601.

37. Barzel, A., Paulk, N.K., Shi, Y., Huang, Y., Chu, K., Zhang, F., Valdmanis, P.N., Spector, L.P., Porteus, M.H., Gaensler, K.M., and Kay, M.A. (2015). Promoterless gene targeting without nucleases ameliorates haemophilia B in mice. Nature *517*, 360–364. https://doi.org/10.1038/nature13864.

38. Sharma, R., Anguela, X.M., Doyon, Y., Wechsler, T., DeKelver, R.C., Sproul, S., Paschon, D.E., Miller, J.C., Davidson, R.J., Shivak, D., et al. (2015). In vivo genome editing of the albumin locus as a platform for protein replacement therapy. Blood *126*, 1777–1784. https://doi.org/10.1182/blood-2014-12-615492.

39. Zhao, L., Yang, Z., Zheng, M., Shi, L., Gu, M., Liu, G., Miao, F., Chang, Y., Huang, F., and Tang, N. (2024). Recombinant adeno-associated virus 8 vector in gene therapy: Opportunities and challenges. Genes Dis. *11*, 283–293. https://doi.org/10.1016/j.gendis.2023.02.010.

40. Gao, G.-P., Alvira, M.R., Wang, L., Calcedo, R., Johnston, J., and Wilson, J.M. (2002). Novel adeno-associated viruses from rhesus monkeys as vectors for human gene therapy. Proc. Natl. Acad. Sci. USA *99*, 11854–11859.

41. Martinez-Turrillas, R., Martin-Mallo, A., Rodriguez-Diaz, S., Zapata-Linares, N., Rodriguez-Marquez, P., San Martin-Uriz, P., Vilas-Zornoza, A., Calleja-Cervantes, M.E., Salido, E., Prosper, F., and Rodriguez-Madoz, J.R. (2022). In vivo CRISPR-Cas9 inhibition of hepatic LDH as treatment of primary hyperoxaluria. Mol. Ther. Methods Clin. Dev. *25*, 137–146. https://doi.org/10.1016/j.omtm.2022.03.006.

42. Esposito, F., Lyubenova, H., Tornabene, P., Auricchio, S., Iuliano, A., Nusco, E., Merlin, S., Olgasi, C., Manni, G., Gargaro, M., et al. (2022). Liver gene therapy with intein-mediated F8 trans -splicing corrects mouse haemophilia A. EMBO Mol. Med. *14*, 151999–e15215. https://doi.org/10.15252/emmm.202115199.

43. McIntosh, J., Lenting, P.J., Rosales, C., Lee, D., Rabbanian, S., Raj, D., Patel, N., Tuddenham, E.G.D., Christophe, O.D., McVey, J.H., et al. (2013). Therapeutic levels of FVIII following a single peripheral vein administration of rAAV vector encoding a novel human factor VIII variant. Blood *121*, 3335–3344. https://doi.org/10.1182/blood-2012-10-462200.

44. Alliegro, M., Ferla, R., Nusco, E., De Leonibus, C., Settembre, C., and Auricchio, A. (2016). Low-dose gene therapy reduces the frequency of enzyme replacement therapy in a mouse model of lysosomal storage disease. Mol. Ther. *24*, 2054–2063. https://doi.org/10.1038/mt.2016.181.

45. Ferla, R., Claudiani, P., Cotugno, G., Saccone, P., De Leonibus, E., and Auricchio, A. (2014). Similar therapeutic efficacy between a single administration of gene therapy and multiple administrations of recombinant enzyme in a mouse model of lysosomal storage disease. Hum. Gene Ther. *25*, 609–618. https://doi.org/10.1089/hum.2013.213.

46. Bowen, D.J. (2002). Haemophilia A and haemophilia B: molecular insights [erratum appears in Mol Pathol 2002 Jun;55(3):208]. Mol Pathol. *55*, 127–144.

47. Nathwani, A.C., Tuddenham, E., Chowdary, P., McIntosh, J., Lee, D., Rosales, C., Phillips, M., Pie, J., Junfang, Z., Meagher, M.M., et al. (2018). GO-8: Preliminary Results of a Phase I/II Dose Escalation Trial of Gene Therapy for Haemophilia a Using a Novel Human Factor VIII Variant. Blood *132*, 489. https://doi.org/10.1182/blood-2018-99-118256.

48. Peyvandi, F., Mannucci, P.M., Garagiola, I., El-Beshlawy, A., Elalfy, M., Ramanan, V., Eshghi, P., Hanagavadi, S., Varadarajan, R., Karimi, M., et al. (2016). A Randomized Trial of Factor VIII and Neutralizing Antibodies in Hemophilia A. N. Engl. J. Med. *374*, 2054–2064. https://doi.org/10.1056/nejmoa1516437.

49. D'avanzo, F., Zanetti, A., De Filippis, C., and Tomanin, R. (2021). Mucopolysaccharidosis type vi, an updated overview of the disease. Int. J. Mol. Sci. *22*, 13456. https://doi.org/10.3390/ijms222413456.

50. Harmatz, P., and Shediac, R. (2017). Mucopolysaccharidosis VI: Pathophysiology, diagnosis and treatment. Front. Biosci. *22*, 385–406. https://doi.org/10.2741/4490.

51. Sestito, S., Rinninella, G., Rampazzo, A., D'Avanzo, F., Zampini, L., Santoro, L., Gabrielli, O., Fiumara, A., Barone, R., Volpi, N., et al. (2022). Cardiac involvement in MPS patients: incidence and response to therapy in an Italian multicentre study. Orphanet J. Rare Dis. *17*, 251–312. https://doi.org/10.1186/s13023-022-02396-5.

52. Muenzer, J. (2011). Overview of the mucopolysaccharidoses. Rheumatology *50*, 4–12. https://doi.org/10.1093/rheumatology/ker394.

53. Evers, M., Saftig, P., Schmidt, P., Hafner, A., McLoghlin, D.B., Schmahl, W., Hess, B., Von Figura, K., and Peters, C. (1996). Targeted disruption of the arylsulfatase B gene results in mice resembling the phenotype of mucopolysaccharidosis VI. Proc. Natl. Acad. Sci. USA *93*, 8214–8219. https://doi.org/10.1073/pnas.93.16.8214.

54. Turchiano, G., Andrieux, G., Klermund, J., Blattner, G., Pennucci, V., el Gaz, M., Monaco, G., Poddar, S., Mussolino, C., Cornu, T.I., et al. (2021). Quantitative evaluation of chromosomal rearrangements in gene-edited human stem cells by CAST-Seq. Cell Stem Cell *28*, 1136–1147.e5. https://doi.org/10.1016/j.stem.2021.02.002.

55. Wang, L., Yang, Y., Breton, C.A., White, J., Zhang, J., Che, Y., Saveliev, A., McMenamin, D., He, Z., Latshaw, C., et al. (2019). CRISPR/Cas9-mediated in vivo gene targeting corrects hemostasis in newborn and adult factor IX-knockout mice. Blood *133*, 2745–2752. https://doi.org/10.1182/blood.2019000790.

56. Deyle, D.R., and Russell, D.W. (2009). Adeno-associated virus vector integration. Curr. Opin. Mol. Ther. *11*, 442–447.

57. Sabatino, D.E., Bushman, F.D., Chandler, R.J., Crystal, R.G., Davidson, B.L., Dolmetsch, R., Eggan, K.C., Gao, G., Gil-Farina, I., Kay, M.A., et al. (2022). Evaluating the state of the science for adeno-associated virus integration: An integrated perspective. Mol. Ther. *30*, 2646–2663. https://doi.org/10.1016/j.ymthe.2022.06.004.

58. Chandler, R.J., LaFave, M.C., Varshney, G.K., Burgess, S.M., and Venditti, C.P. (2016). Genotoxicity in mice following AAV gene delivery: A safety concern for human gene therapy? Mol. Ther. *24*, 198–201. https://doi.org/10.1038/mt.2016.17.

59. Hanlon, K.S., Kleinstiver, B.P., Garcia, S.P., Zaborowski, M.P., Volak, A., Spirig, S.E., Muller, A., Sousa, A.A., Tsai, S.Q., Bengtsson, N.E., et al. (2019). High levels of AAV vector integration into CRISPR-induced DNA breaks. Nat. Commun. *10*, 4439–4511. https://doi.org/10.1038/s41467-019-12449-2.

60. Nelson, C.E., Wu, Y., Gemberling, M.P., Oliver, M.L., Waller, M.A., Bohning, J.D., Robinson-Hamm, J.N., Bulaklak, K., Castellanos Rivera, R.M., Collier, J.H., et al. (2019). Long-term evaluation of AAV-CRISPR genome editing for Duchenne muscular dystrophy. Nat. Med. *25*, 427–432. https://doi.org/10.1038/s41591-019-0344-3.

61. Simpson, B.P., Yrigollen, C.M., Izda, A., and Davidson, B.L. (2023). Targeted long-read sequencing captures CRISPR editing and AAV integration outcomes in brain. Mol. Ther. *31*, 760–773. https://doi.org/10.1016/j.ymthe.2023.01.004.

62. Chandler, R.J., La Fave, M.C., Varshney, G.K., Trivedi, N.S., Carrillo-Carrasco, N., Senac, J.S., Wu, W., Hoffmann, V., Elkahloun, A.G., Burgess, S.M., and Venditti, C.P. (2015). Vector design influences hepatic genotoxicity after adeno-associated virus gene therapy. J. Clin. Invest. *125*, 870–880. https://doi.org/10.1172/JCI79213.

63. Ferla, R., Alliegro, M., Dell'Anno, M., Nusco, E., Cullen, J.M., Smith, S.N., Wolfsberg, T.G., O'Donnell, P., Wang, P., Nguyen, A.D., et al. (2021). Low incidence of hepatocellular carcinoma in mice and cats treated with systemic adeno-associated viral vectors. Mol. Ther. Methods Clin. Dev. *20*, 247–257. https://doi.org/10.1016/j.omtm.2020.11.015.

64. Hinderer, C., Katz, N., Buza, E.L., Dyer, C., Goode, T., Bell, P., Richman, L.K., and Wilson, J.M. (2018). Severe Toxicity in Nonhuman Primates and Piglets Following High-Dose Intravenous Administration of an Adeno-Associated Virus Vector Expressing Human SMN. Hum. Gene Ther. *29*, 285–298. https://doi.org/10.1089/hum.2018.015.

65. (2020). High-dose AAV gene therapy deaths. Nat. Biotechnol. *38*, 910. https://doi.org/10.1038/s41587-020-0642-9.

66. Han, J.P., Kim, M., Choi, B.S., Lee, J.H., Lee, G.S., Jeong, M., Lee, Y., Kim, E.A., Oh, H.K., Go, N., et al. (2022). In vivo delivery of CRISPR-Cas9 using lipid nanoparticles enables antithrombin gene editing for sustainable hemophilia A and B therapy. Sci. Adv. *8*, eabj6901-10. https://doi.org/10.1126/sciadv.abj6901.

67. Lampe, S.E.G., Kaspar, B.K., and Foust, K.D. (2014). Intravenous injections in neonatal mice. J. Vis. Exp. *11*, 2–7. https://doi.org/10.3791/52037.

68. Nakai, H., Fuess, S., Storm, T.A., Muramatsu, S.i., Nara, Y., and Kay, M.A. (2005). Unrestricted Hepatocyte Transduction with Adeno-Associated Virus Serotype 8 Vectors in Mice. J. Virol. *79*, 214–224. https://doi.org/10.1128/jvi.79.1.214-224.2005.

69. Tornabene, P., Trapani, I., Centrulo, M., Marrocco, E., Minopoli, R., Lupo, M., Iodice, C., Gesualdo, C., Simonelli, F., Surace, E.M., and Auricchio, A. (2021). Inclusion of a degron reduces levelsof undesired inteins after AAV-mediated proteintrans-splicing in the retina. Mol. Ther. Methods Clin. Dev. *23*, 448–459. https://doi.org/10.1016/j.omtm.2021.10.004.

70. Auricchio, A., Hildinger, M., O'Connor, E., Gao, G.P., and Wilson, J.M. (2001). Isolation of highly infectious and pure adeno-associated virus type 2 vectors with a single-step gravity-flow column. Hum. Gene Ther. *12*, 71–76. https://doi.org/10.1089/104303401450988.

71. Doria, M., Ferrara, A., and Auricchio, A. (2013). AAV2/8 vectors purified from culture medium with a simple and rapid protocol transduce murine liver, muscle, and retina efficiently. Hum. Gene Ther. Methods *24*, 392–398. https://doi.org/10.1089/hgtb.2013.155.

72. Wang, F., Flanagan, J., Su, N., Wang, L.C., Bui, S., Nielson, A., Wu, X., Vo, H.T., Ma, X.J., and Luo, Y. (2012). RNAscope: A novel in situ RNA analysis platform for formalin-fixed, paraffin-embedded tissues. J. Mol. Diagn. *14*, 22–29. https://doi.org/10.1016/j.jmoldx.2011.08.002.

73. Rhiel, M., Geiger, K., Andrieux, G., Rositzka, J., Boerries, M., Cathomen, T., and Cornu, T.I. (2023). T-CAST: An optimized CAST-Seq pipeline for TALEN confirms superior safety and efficacy of obligate-heterodimeric scaffolds. Front. Genome Ed. *5*, 1130736–1130814. https://doi.org/10.3389/fgeed.2023.1130736.

74. Currin, A., Swainston, N., Dunstan, M.S., Jervis, A.J., Mulherin, P., Robinson, C.J., Taylor, S., Carbonell, P., Hollywood, K.A., Yan, C., et al. (2019). Highly multiplexed, fast and accurate nanopore sequencing for verification of synthetic DNA constructs and sequence libraries. Synth. Biol. *4*, ysz025-8. https://doi.org/10.1093/synbio/ysz025.

75. Tange, O. (2018). GNU Parallel 2018 (Ole Tange).

76. Srivathsan, A., Lee, L., Katoh, K., Hartop, E., Narayanan Kutty, S., Wong, J., Yeo, D., and Meier, R. (2021). MinION barcodes: biodiversity discovery and identification by everyone, for everyone. Preprint at bioRxiv. https://doi.org/10.1186/s12915-021-01141-x.

77. Li, H. (2018). Minimap2: Pairwise alignment for nucleotide sequences. Bioinformatics *34*, 3094–3100. https://doi.org/10.1093/bioinformatics/bty191.

78. Danecek, P., Bonfield, J.K., Liddle, J., Marshall, J., Ohan, V., Pollard, M.O., Whitwham, A., Keane, T., McCarthy, S.A., Davies, R.M., and Li, H. (2021). Twelve years of SAMtools and BCFtools. GigaScience *10*, giab008-4. https://doi.org/10.1093/gigascience/giab008.

79. Clement, K., Rees, H., Canver, M.C., Gehrke, J.M., Farouni, R., Hsu, J.Y., Cole, M.A., Liu, D.R., Joung, J.K., Bauer, D.E., and Pinello, L. (2019). CRISPResso2 provides accurate and rapid genome editing sequence analysis. Nat. Biotechnol. *37*, 224–226. https://doi.org/10.1038/s41587-019-0032-3.

80. Li, H. (2013). Aligning Sequence Reads, Clone Sequences and Assembly Contigs with BWA-MEM. Preprint at arXiv. https://doi.org/10.48550/arXiv.1303.3997.

81. Concordet, J.P., and Haeussler, M. (2018). CRISPOR: Intuitive guide selection for CRISPR/Cas9 genome editing experiments and screens. Nucleic Acids Res. *46*, W242–W245. https://doi.org/10.1093/nar/gky354.

STAR★METHODS

KEY RESOURCES TABLE

REAGENT or RESOURCE	SOURCE	IDENTIFIER
**Antibodies**		
2A	Novus Biologicals	Cat#NBP259627H, RRID:AB_3101802
Cas9	Thermo Fisher Scientific	Cat# MA1-201, RRID:AB_2610640
Calnexin	Enzo Life Sciences	Cat#ADI-SPA-860F, RRID:AB_11178981
hARSB polyclonal antibody	Covalab	Custom
**Chemicals, peptides and recombinant proteins**		
SubX	Leica Biosystems	Cat# 3803670E
Alcian blue	Merck	Cat#A5268-25G
Nuclear Fast Red	Merck	Cat#N8002-5G
Aluminum sulfate hydrate 98%	Merck	Cat# 36,845-8
**Critical commercial assays**		
Coatest® SP4 FVIII-kit	Chromogenix, Werfen, Milan	Cat#K824094
Activated partial thromboplastin time (aPTT)	Teco, Bunde, Germany	Coatron M4
Mouse albumin ELISA kit	Abcam	Cat# 108791
VisuLizeFVIII ELISA kit	Affinity Biologicals	Cat# FVIII-AG
**Deposited data**		
BSgenome.Mmusculus.UCSC.mm10 1.4.3	Bioconductor	https://bioconductor.org/packages/release/data/annotation/html/BSgenome.Mmusculus.UCSC.mm10.html
TxDb.Mmusculus.UCSC.mm10.knownGene 3.10.0	Bioconductor	https://bioconductor.org/packages/release/data/annotation/html/TxDb.Mmusculus.UCSC.mm10.knownGene.html
org.Mm.e.g.,.db 3.16.0	Bioconductor	https://bioconductor.org/packages/release/data/annotation/html/org.Mm.eg.db.html
**Experimental models: Organisms/strains**		
C57BL/6 mice	Envigo	Cat#057
B6; 129S F8tm1Kaz/J	Jackson Laboratory	Cat#004424
MPS VI mice	Institute of Molecular Medicine and Cell Research, University of Freiburg, Germany	N/A
**Software and algorithms**		
Fiji	ImageJ	http://rsbweb.nih.gov/ij/
GraphPad	Prism GraphPad	https://www.graphpad.com/features
Benchling	Benchling	www.benchling.com
Zen Blue	ZEISS ZEN	https://www.zeiss.com/microscopy/en/products/software/zeiss-zen-lite.html
Qpath	Qpath	https://qupath.github.io/
Biorender	Biorender	https://www.biorender.com/
CRISPResso2	CRISPResso2	http://crispresso2.pinellolab.org/submission
CRISPOR web tool	CRISPOR web tool	CRISPOR.org
MinKNOW version 23.04.6	Oxford Nanopore Technologies	https://nanoporetech.com
Minimap2 v. 2.24-r1122	minimap2	https://github.com/lh3/minimap2/releases
BBmap	SOURGEFORGE	https://sourceforge.net/projects/bbmap/
BWA MEM v0.7.17	SOURGEFORGE	https://bio-bwa.sourceforge.net/bwa.shtml
GNU parallel 20220922	GNU Operating System	https://www.gnu.org/software/parallel/
Samtools 1.16.1	Samtools	http://www.htslib.org/

(*Continued on next page*)

Continued

REAGENT or RESOURCE	SOURCE	IDENTIFIER
Pcregrep 8.45 2021-06-15		https://man7.org/linux/man-pages/man1/pcregrep.1.html
BBDuk	BBTools	https://jgi.doe.gov/data-and-tools/software-tools/bbtools/bb-tools-user-guide/bbduk-guide/
bedtools v2.27.1	Bedtools website	https://bedtools.readthedocs.io
Biostrings 2.46.0	Bioconductor	https://bioconductor.org/packages/release/bioc/html/Biostrings.html
ChIPseeker 1.14.2	Bioconductor	https://bioconductor.org/packages/release/bioc/html/ChIPseeker.html
CAST-Seq	CAST-Seq github	https://github.com/AG-Boerries/CAST-Seq
R 4.2.2	CRAN	https://www.r-project.org
Other		
TaqMan-PCR	Applied Biosystems	Cat# 4304437
O.C.T. matrix	Kaltek	Cat# 0782
Vectashield with DAPI	Vector Laboratories	Cat# H-1200-10
Trisodium citrate 0.109 M	BD	Cat# 5T31.363048
Bradford Reagent	Bio-Rad	Cat#5000006
Protease inhibitor cocktail	Merck	Cat#78430
Ponceau	Merck	Cat#P7170-1L
KAPA HiFi Hotstart Polymerase	Roche	Cat#07958927001
Ampure XP beads	Beckman Coulter	Cat# A63881
DNeasy Blood & Tissue kit	Qiagen	Cat# 69504
QIAquick® Gel Extraction Kit	Qiagen	Cat# 28706
Ligation Sequencing Kit V14	Oxford Nanopore Technologies	Cat# SQK-LSK114
LightCycler 480 SYBR Green I Master	Roche	Cat#04707516001

RESOURCE AVAILABILITY

Lead contact

Further information and requests for resources and reagents as well as datasets and protocols should be directed to and will be fulfilled by the lead contact, Alberto Auricchio (auricchio@tigem.it).

Materials availability

This study did not generate new unique reagents.

Data and code availability

- All data reported in this paper will be shared by the lead contact upon request.
- This study does not report original code.
- Any additional information required to reanalyze the data reported in this work paper is available from the lead contact upon request.

EXPERIMENTAL MODEL AND SUBJECT DETAILS

Animal models

Mice were housed at the TIGEM animal house (Pozzuoli, Italy) and maintained under a 12 h light/dark cycle at 23 ± 1°C and humidity of 50% ± 5% with food and water available *ad libitum*. Animals were raised in accordance with the Institutional Animal Care and Use Committee guidelines for the care and use of animals in research. C57BL/6J mice were purchased from Envigo Italy SRL (Udine, Italy). The hemophilic A (HemA) mouse model (Cat# B6; 129S-F8tm1Kaz/J) was imported from the Jackson Laboratory (JAX stock). The MPS VI mice were kindly provided by Prof. C. Peters (Institute of Molecular Medicine and Cell Research, University of Freiburg, Germany). HemA mice were maintained by crossing knockout homozygous females with knockout hemizygous males to produce knockout experimental mice. MPS VI mice were maintained as heterozygotes and crossed to produce homozygous knockout experimental mice.

METHODS DETAILS

Study design

This study was designed to evaluate the efficacy and safety of the AAV vector mediated HITI approach in the liver. Editing efficiency in the liver was defined in cryo-section fluorescent images by using the ImageJ software to count and calculate the percentage of hepatocyte positive for the *Discosoma* sp.-Red (dsRed) fluorescent protein. In all *in vivo* studies, mice were randomly assigned to each treatment group. Therapeutic efficacy in the liver was assessed by evaluating the impact of neonatal systemic delivery of AAV-HITI on the phenotype of two different animal models of Mucopolysaccharidosis type VI (MPS VI) and Hemophilia A (HemA) while for safety studies only MPS VI mice were used. In addition, in the studies involving the MPS VI disease model, female and male mice were considered equivalent and randomly assigned to treatment groups whereas in the study involving hemophilic animals only males were used. Littermate controls were used when available. In both cases, observers were blind to both genotype and treatment of the animals. Sample sizes were determined based on previous experience and technical feasibility. Any difference in the numbers of analyzed samples within the same group of treatment at different timepoints was due to sample availability.

Systemic vector administration

Studies in animals were carried out in accordance with the Italian Ministry of Health regulation for animal procedures (Ministry of Health authorization number: 352/2020-PR and 626/2022-PR). The injections were performed under general anesthesia, as previously described.[21] Temporal vein injections in neonatal mice (C57BL/6J or MPS VI, or HemA) were performed at post-natal p1-2 following the protocol published by Gombash Lampe et *al*..[67] For adult treatments, (C57BL/6J, MPS VI or HemA) retro-orbital injections were performed. The following doses were used in the experiments aimed at evaluating HITI efficiency in C57BL/6J and MPSVI newborn mice: high doses 1.2×10^{14} total genome copies (GC/Kg; 6×10^{13} GC/kg for each vector); medium doses 3.9×10^{13} total GC/Kg (1.95×10^{13} GC/kg for each vector); low doses 1.2×10^{13} total GC/Kg (6×10^{12} GC/kg for each vector). For rescue experiments in neonatal HemA mice, we used 3.9×10^{13} total GC/Kg (1.95×10^{13} GC/kg for each vector). For rescue experiments in MPS VI and Hema adult mice, we used 1.2×10^{13} total GC/Kg (6×10^{12} GC/kg for each vector). AAV8 vectors were used for all *in vivo* studies because of their high liver transduction efficiency.[68]

Generation of the AAV vector plasmids

The plasmids used for AAV vector production were derived from either the pAAV2.1[21] or the pTIGEM[69] plasmids both containing the inverted terminal repeats of AAV serotype 2.[70] The mouse albumin (m*Alb*) gRNA (5′-GTATTTAATAGGCAGCAGTG-3′) was selected using the benchling gRNA design tool (www.benchling.com), targeting the intron 13 of the albumin locus considering the best predicted on-target and off-target scores. The scramble RNA was designed so as not to align with any sequences in the mouse genome.

AAV vector production and characterization

AAV serotype 8 vectors (AAV8) were produced by InnovaVector s.r.l by triple transfection of HEK293 cells followed by two rounds of $CsCl_2$ purification.[71] For each viral preparation, vector titers (genome copies/ml) were determined by averaging the titer achieved by dot-blot and by TaqMan-PCR (Cat#4304437, Applied Biosystems, Carlsbad, California, USA) quantification analysis.[71]

Liver fluorescence imaging

To evaluate dsRed expression in liver, C57BL/6J mice were injected at post-natal day (P) 1–2. Livers were harvested at p30 under anesthesia as previously described.[21] A small piece of each lobe was dissected, fixed in 4% PFA overnight, infiltrated with 15% sucrose over the course of a day and 30% sucrose overnight before being included in O.C.T. matrix (Cat# 0782, Kaltek) for cryo-sectioning. Five-μm-thick liver cryo-sections were cut, distributed on slides, and mounted with Vectashield with DAPI (Cat#H-1200-10, Vector Lab). Cryo-sections were analyzed under a confocal LSM-700 microscope (Carl Zeiss), using appropriate excitation and detection settings for dsRed and DAPI. For assessment of HITI efficiency in mouse liver cryo-sections, three to four images of each liver were acquired at 20× magnification and then analyzed using ImageJ (Fiji) software (http://rsbweb.nih.gov/ij/) as previously described.[21] We counted a minimum of 900 hepatocytes, identified by DAPI staining of the nucleus, for each image. The hepatocytes expressing the dsRed as result of the integration were unequivocally identified and counted based on their shape. The final value (∼15%) was then obtained by dividing the number of dsRed positive hepatocytes over the total number of DAPI cells and multiplied by 100 to obtain the percentage (%).

In situ hybridization

Liver cryo-sections were used for the *in situ* hybridization (ISH) with the technology of Basescope (Advanced Cell Diagnostic, USA) in accordance with the manufacturer's protocol.[72] ISH labeling of HITI donor DNA was performed using a 3-ZZ paired probe (sense) (Cat# 1134401, ACD). ISH was performed using the BaseScope Duplex Reagent Kit (Cat# 323871, ACD). Custom pre-treatment conditions included target retrieval of 30 min at 95 C–100 C, RNAscope Protease III (Cat # 322380, ACD) for 30 min at room temperature (RT). Custom counterstaining with Mayer haematoxylin for 1 min at RT was performed after probe, amplification, and chromogen steps in the kit assay. Sections were analyzed with scanned with ZEISS Axio Scan.Z1. The whole digital slides were viewed by zen blue software. Red positive spots of HITI DNA were quantified with QuPath software. For the quantitative analysis of positive

signals, we selected 4–6 liver regions at 20× magnification from mice belonging to AAV-HITIgRNA, -scRNA and PBS experimental groups, and the results were expressed as a percentage of positive nuclei over the total nuclei analyzed.

Serum ARSB enzymatic activity

Blood samples were collected at different timepoints from AAV-HITI-treated and control MPS VI mice as previously reported.[21] Serum ARSB activity was measured by an immune capture assay based on the use of a specific custom-made anti-hARSB polyclonal antibody (Covalab, Villeurbanne, France) following a previously described protocol.[21]

Chromogenic assay

Blood sampling was performed by retro-orbital withdrawal and nine parts of blood were collected into one part of buffered trisodium citrate 0.109 M (Cat#5T31.363048; BD, Franklin Lakes, NJ, USA). Plasma was collected after centrifugation at 3.000 rpm at 4°C for 15 min. Chromogenic assay was performed on plasma samples using the Coatest SP4 FVIII-kit (Cat# K824094; Chromogenix, Werfen, Milan, Italy) according to the manufacturer's instructions. Results are expressed as International Units (IU) per decilitre (dL).

FVIII antigen detection

To quantify the levels of FVIII antigen, an ELISA kit (Cat#FVIII-AG; VisuLize FVIII ELISA kit, Affinity Biologicals, Arcore, Italy) was used according to the manufacturer's instructions.

GAG level analysis in urine and tissues

Urine samples were collected over 24 h using metabolic cages at p60, p90, p180, p270 and p360 from MPS VI-treated and control mice. Samples were centrifuged briefly to remove debris and diluted 1:50 in water to measure GAGs content. Fifty μL of diluted urine or 250 μg of protein lysate were then used for GAGs evaluation as previously reported.[21]

Alcian blue staining in histological sections

After methacarn fixation, livers, kidneys, spleens, and hearts were dehydrated by immersion in increasing concentrations of alcohol (70%, 80%, 90%, 100%) and then in Sub-X. All tissues were embedded in paraffin and sectioned transversally into 7-μm-thick serial sections on a microtome. Tissue sections were de-paraffinized, rehydrated, then washed in water and stained with 1% Alcian blue (Cat#A5268-25G, Merck) in hydrochloric acid (5 min for hearts, 60 min all remaining tissues). Counter-staining was performed with 0.1% Fast-Red (Cat#N8002-5G, Merck) in water for 1 min. Liver, kidney, and spleen sections stained with Alcian blue were imaged with Leica Microscope DM5500. Heart sections stained with Alcian blue were scanned with ZEISS Axio Scan.Z1. The whole digital slides were viewed by Zen Blue software (https://www.zeiss.com/microscopy/en/products/software/zeiss-zen-lite.html). Quantitative analyses of Alcian blue staining in myocardial tissue and in mitral valves were performed by Qpath software (https://qupath.github.io/). Alcian blue quantification in myocardium was measured in two fields of identical area. Alcian blue quantification in the mitral valve was performed on the entirety of the valve. Results are expressed as Alcian blue positive area/total area.

Bone analysis

Radiography images were performed on AAV-HITI-treated MPS VI mice and unaffected controls were anesthetized with an intraperitoneal injection of ketamine (10 mg/Kg) combined with medetomidine (1 mg/Kg). Skull weight and length, and tibia and femur length, were measured using ImageJ (Fiji) software (http://rsbweb.nih.gov/ij/).

Tail-clip assay

Mice were anesthetized and the distal part of the tail was cut at 2–3 mm of diameter and immediately put in a prewarmed 0.9% saline solution and allowed to bleed for 10 min without disturbance and the tails were then cauterized. The mixture of collected blood and physiological saline solution was centrifuged at 1500 g for 5 min and the total volume of blood lost was measured.

Activated partial thromboplastin time (aPTT) assay

Activated partial thromboplastin time (aPTT) was measured on plasma samples with Coatron M4 (Teco, Bünde, Germany) using the aPTT program following the manufacturer's manual.

Western blot analysis

Protein concentration in AAV-HITI-treated sera samples collected from MPS VI and HemA mice was determined by Bradford Reagent (Cat#5000006, Bio-Rad). Ten microgram of sera were denatured at 100°C for 5 min in 1× Laemmli sample buffer supplemented with 1 M dithiothreitol (DTT). Next, protein samples were resolved on a 4–15% SDS–PAGE and transferred onto polyvinylidene difluoride (PVDF) membrane. After transfer to PVDF membrane, blot was blocked with TBS-Tween-20 containing 5% non-fat milk for 1 h at room temperature followed by incubation with primary antibody (Cat#NBP259627H,Novus Biologicals; dilution: 1/2,000) 1h at 4°C. Ponceau (Cat#P7170-1L, Merck) was used as normalizer in sera blot. Liver specimens were mechanically homogenized using metal beads and lysed in RIPA buffer, supplemented with protease inhibitor cocktail (Cat#78430, Merck). Samples were incubated for 30 min on ice, vortexed every 10 min, and centrifuged at 16,200 *g* for 20 min. Pellets were discarded and lysates were used

for Western blot analyses. After lysis, protein samples were treated as mentioned above. Hundred micrograms of liver proteins were loaded for each specimen into a 6% SDS–PAGE; after transfer to PVDF membrane, blot was blocked with TBS-Tween-20 containing 5% non-fat milk for 1 h at room temperature followed by incubation with primary antibody overnight at 4°C. The primary antibodies used for immuno-blotting were: rabbit anti-*Sp*Cas9 (Cat# MA1-201, Thermo Fisher Scientific; dilution: 1/1,000) mouse anti-Calnexin (Cat#ADI-SPA-860F, Enzo Life Sciences; dilution: 1/2,000).

Serum albumin measurement

Blood was collected at p360 from AAV-HITI-treated and control mice via eye bleeding and centrifuged as previously described.[21] Serum samples were diluted 1:30.000 and analyzed with a mouse albumin ELISA kit (Cat#108791, Abcam, Cambridge, UK) following the manufacturer's instructions.

Histopathological analysis

Right, left, median, and caudal liver lobes were embedded in paraffin and sectioned at a thickness of 5 μm. To assess histological features, Haematoxylin/Eosin (Diapath) staining was performed according to standard protocols and samples were analyzed by an expert histopathologist from Histopathology Unit (Cogentech Ltd. Benefit Corporation, Milan) in blind.

DNA extraction

DNA extraction was performed using the DNeasy Blood & Tissue kit (Cat# 69504, QIAGEN) following the manufacturer's instructions.

Quantitative PCR (qPCR)

Viral genome copies (GC) were measured by qPCR analysis in 100ng of genomic DNA extracted from AAV-HITI-treated liver samples at 1-week, -month and 1-year upon AAV-HITI administration, using the LightCycler 480 SYBR Green I Master mix (Cat#04707516001, Roche). The following primers: forward HLP: 5′-CTCCTCCGATAACTGGGGTGAC-3′ and reverse HLP: 5′-GCCCTGTCCTC GTCCGTATTTA-3′, were used to detect AAV8 -*Sp*Cas9 vector; forward BGH 5′-TCTAGTTGCCAGCCATCTGTTGT-3'; reverse BGH 5′-TGGGAGTGGCACCTTCCA-3′ were used to detect the AAV-HITI donor DNA following the qPCR protocol from Roche. Briefly, enzyme activation was set at 95°C during 20 s −3 min (1 cycle), followed by the denaturation step at 95°C during 3 s and annealing extension and acquisition at 60°C at 20 s with the last two points set at 40 cycles.

CAST-Seq

To identify chromosomal aberrations, nominate off-target sites and analyze integration events at the on-target site, we performed CAST-Seq.[54] High-throughput sequencing libraries were essentially prepared according to Turchiano et al., 2021.[54] In search for off-targets, a third decoy primer was included to prevent predominant amplification of the target locus after successful HITI. In total, three CAST-Seq libraries from liver genomic DNA samples extracted from three different 1 year-old MPS VI mice treated as newborn with high doses of AAV-HITI were prepared and compared to a single library generated from control mice samples (scRNA). In order to analyze in depth the integration of AAVs and parts thereof, two CAST-Seq libraries of HITI-treated and a single library of a control mouse were sequenced. For these samples, the HITI-specific decoy primer was not used during library preparation. Sequencing of the samples was outsourced to GeneWiz (division of Azenta Life Sciences) who collected 2x150bp paired-end reads using an Illumina NovaSeq 6000 instrument. The bioinformatics analysis was performed using the previously published pipelines.[54,73]

Nanopore long-read sequencing

Fragments for long-read sequencing were amplified from DNA extracted from 1 year-old MPS VI mice treated as newborn with high doses of AAV-HITI using KAPA HiFi Hotstart Polymerase (Cat#07958927001, Roche). The cycling conditions were: 3 min at 98°C followed by 35 cycles of 98°C for 20 s, 61°C (5′ junction) or 63°C (3′ junction) for 30 s, and 4 min at 72°C. A final elongation of 10 min at 72°C was programmed after the last cycle. Multiple PCR reactions were pooled and the pool subjected to bead purification using 0,9X Ampure XP beads (Cat# A63881, Beckman Coulter) and fragments eluted in a small volume of water (30 μL). The thus concentrated PCR pool was subjected to agarose gel electrophoresis and the prominent PCR product was gel-extracted using the QIAquick Gel Extraction Kit (Cat# 28706, Qiagen). With the PCR step, 24-nt long barcodes were introduced, in accordance with previously published data.[74] The 5′ and 3′ junction PCR products were in a next step prepared for sequencing using the Ligation Sequencing Kit V14 (Cat# SQK-LSK114, Oxford Nanopore Technologies) following the manufacturer's instructions. Samples were immediately sequenced on an R10.4.1 MinION Flow Cell (Cat#FLO-MIN114, Oxford Nanopore Technologies) using a MinION sequencing device (Cat#MIN-101B, Oxford Nanopore Technologies) operated with MinKNOW software version 23.04.6. Reads were demultiplexed by employing a custom pipeline that allows for up to 4 mismatches in the barcode sequence using pcregrep and GNU Parallel.[75] We decided to allow up to 4 mismatches based on previously published data[76] as a compromise between a low false annotation rate versus loss of valuable information. Of note, the two samples described here were analyzed together during one sequencing run in the absence of other samples thus reducing the chance of wrongful read assignment. Obtained reads were aligned using Minimap2 v. 2.24-r1122,[77] and further processed with Samtools.[78] ITR sequence insertions were counted from the CIGAR string in a window of 200 bp around the integration site junctions using a custom script.

Short-read sequencing

Assessment of Indels in short sequencing reads was performed using CRISPResso2.[79] For the quantification of ITR sequence integration, raw FASTQ files were processed using BBduk (https://sourceforge.net/projects/bbmap/) and aligned to the reference amplicon using BWA MEM v0.7.17.[80] Correctly mapped reads were extracted from the FASTQ file and aligned to the ITR sequence. The total number of all alignments and the number of all alignments to the ITR sequence were quantified and the relative frequency was calculated.

Off-target site analysis

The top 10 predicted off-target sites were identified using the CRISPOR web tool (CRISPOR.org)[81] based on GRCm39/mm39 mouse genome reference, and sorted by CFD off-target scores. Equal amounts of genomic DNA extracted from 3 AAV-HITI-gRNA-treated livers and 3-scRNA-treated livers were used to amplify between 150 and 300 bp genomic regions flanking the off-targets. PCR products were amplified with specific primers (Table S7) and quantified using Qubit 4.0 fluorometric Assay (Thermo Fisher Scientific). An equal amount of each PCR product from each liver sample was pooled together. Next-generation sequencing (NGS) amplicon library were preprepared from 10 ng of pooled PCR products using the NEGEDIA DNAseq Low Input sequencing service (NEGEDIA s.r.l) which included library preparation, quality assessment and sequencing on a NovaSeq 6000 sequencing system using a paired-end, 2x150 cycle strategy (Illumina Inc.). The resulting FASTQ files were then analyzed using CRISPRessoV2,[79] using the off-targets sequences as reference for analysis. A 17-nucleotide window (upstream and downstream of the cleavage site) was considered for evaluation. NGS analysis was performed by NEGEDIA s.r.l.

QUANTIFICATION AND STATISTICAL ANALYSIS

For all the statistical analysis, we performed the Shapiro-Wilk test to check that each condition had followed the normal distribution (null hypothesis). To assess significant differences between two conditions the non-parametric Mann-Whitney test was used in the case of rejection of the null hypothesis (p-value <0.05). If the null hypothesis was not rejected (p-value ≥ 0.05) the parametric unpaired T-test was applied and the F-test was used to check the homoscedasticity between the two compared conditions. In case of rejection of the null hypothesis (p-value <0.05) the parametric Welch's t-test was applied. For multiple comparisons and post hoc tests, non-parametric Kruskal-Wallis test was used in the case of rejection of the null hypothesis (p-value <0.05). The parametric one-way ANOVA test was applied in case of not rejection of the null hypothesis (p-value ≥ 0.05). In this case, we also performed the Brown-Forsythe test to check the homoscedasticity between the compared conditions (null hypothesis): we used the parametric Welch's one-way ANOVA in case of rejection of the null hypothesis (p-value <0.05). For completeness, we computed the p-values with post hoc tests for the pairwise multiple comparisons: Tukey's test for one-way ANOVA, Dunnet's test for Welch's one-way ANOVA, and Dunn's test for Kruskal-Wallis test. p-values are represented as follows: * p-value <0.05; ** p-value <0.01; *** p-value <0.001; **** p-value <0.0001.

Cell Reports Medicine, Volume *5*

Supplemental information

Safe and effective liver-directed AAV-mediated

homology-independent targeted integration

in mouse models of inherited diseases

Federica Esposito, Fabio Dell'Aquila, Manuel Rhiel, Stefano Auricchio, Kay Ole Chmielewski, Geoffroy Andrieux, Rita Ferla, Paula Sureda Horrach, Arjun Padmanabhan, Roberto Di Cunto, Simone Notaro, Manel Llado Santeularia, Melanie Boerries, Margherita Dell'Anno, Edoardo Nusco, Agnese Padula, Sofia Nutarelli, Tatjana I. Cornu, Nicolina Cristina Sorrentino, Pasquale Piccolo, Ivana Trapani, Toni Cathomen, and Alberto Auricchio

Figure S1

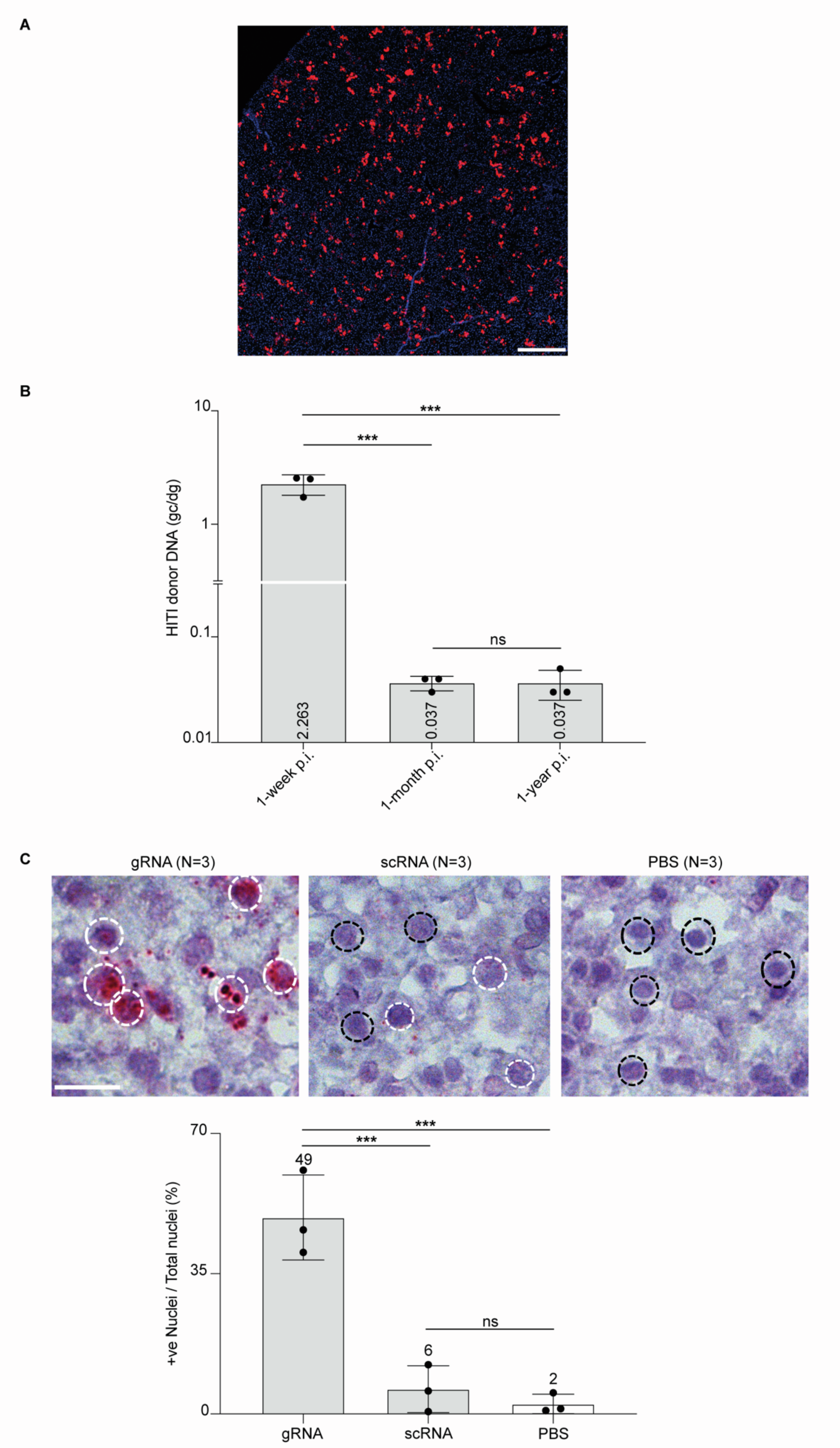
A
B
10
1
0.1
0.01
HITI donor DNA (gc/dg)
***
***
ns
2.263
0.037
0.037
1-week p.i.
1-month p.i.
1-year p.i.
C
gRNA (N=3)
scRNA (N=3)
PBS (N=3)
70
35
0
+ve Nuclei / Total nuclei (%)
***
***
ns
49
6
2
gRNA
scRNA
PBS

Figure S1. AAV-HITI Ds-Red. Related to Figure 2. A) Representative fluorescence microscopy low magnification image of an OCT liver cryo-section from a wild-type mouse injected with AAV-HITI (gRNA) at the total dose of 1.2x10^14 total GC/kg. Scale bar 500 µm. **B**) Real-Time PCR analysis of HITI Donor vector genome in liver of AAV-HITIgRNA-treated newborn mice at different timepoints. Data are represented as mean ± standard deviation of HITI donor DNA genome copies/diploid genomes (gc/dg). For each timepoint N=3 samples were analyzed. Statistical differences were assessed by Ordinary one-way ANOVA Test followed by Tukey's multiple comparisons test. P-value ***= 0.0001 between 1-week and 1-month; p-value ***= 0.0001 between 1-week and 1-year; p-value >0.999 between 1-month and 1-year. **C**) In situ Hybridization (ISH) of HITI donor DNA on liver sections. Upper panel: representative images of ISH in liver sections from (N=3) gRNA, (N=3) scRNA and (N=3) PBS experimental groups; Nuclei are stained in blue. White circles: positive nuclei; Black circles: negative nuclei; Scale bar of all images represent 25µm. Lower panel: quantification of positive nuclei over the total counted nuclei (+ve Nuclei/Total nuclei). Statistical differences were assessed by Ordinary one-way ANOVA Test followed by Tukey's multiple comparisons test. P-value *** = 0.0008 between gRNA and scRNA samples; p-value ***= 0.0005 between gRNA and PBS samples. Data are represented as mean ± standard deviation.

Figure S2

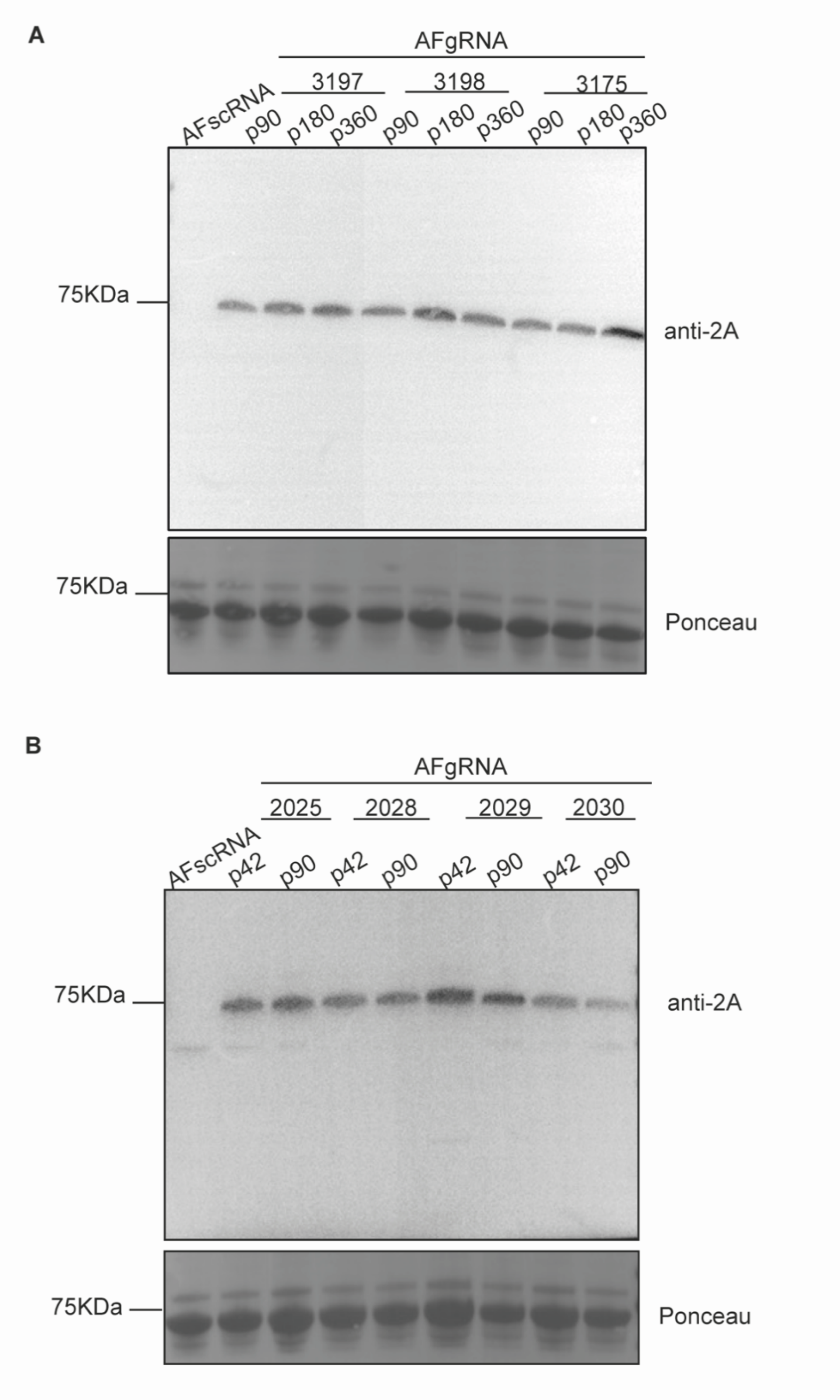

Figure S2. Detection of the modified albumin 2A (Alb-2A) in sera from treated animals. **Related to Figure 2**. **A**) Western blot analysis performed on 10 µg of proteins extracted from sera samples collected from AAV-HITI-treated MPS VI or HemA (**B**) mice to detect the modified Alb-2A (AFgRNA MPS VI N=3: 3197,3198,3175; AFgRNA HemA N=4: 2025,2028,2029,2030). In **A**) 3 different timepoints were analyzed: p90, p180 and p180 days post AAV-HITI neonatal delivery. In **B**) 2 different timepoints were analyzed: p42 and p90. An affected AAV-HITI scRNA-treated mouse (AFscRNA; MPS VI in **A** or HemA in **B**) was used as negative control.

Figure S3

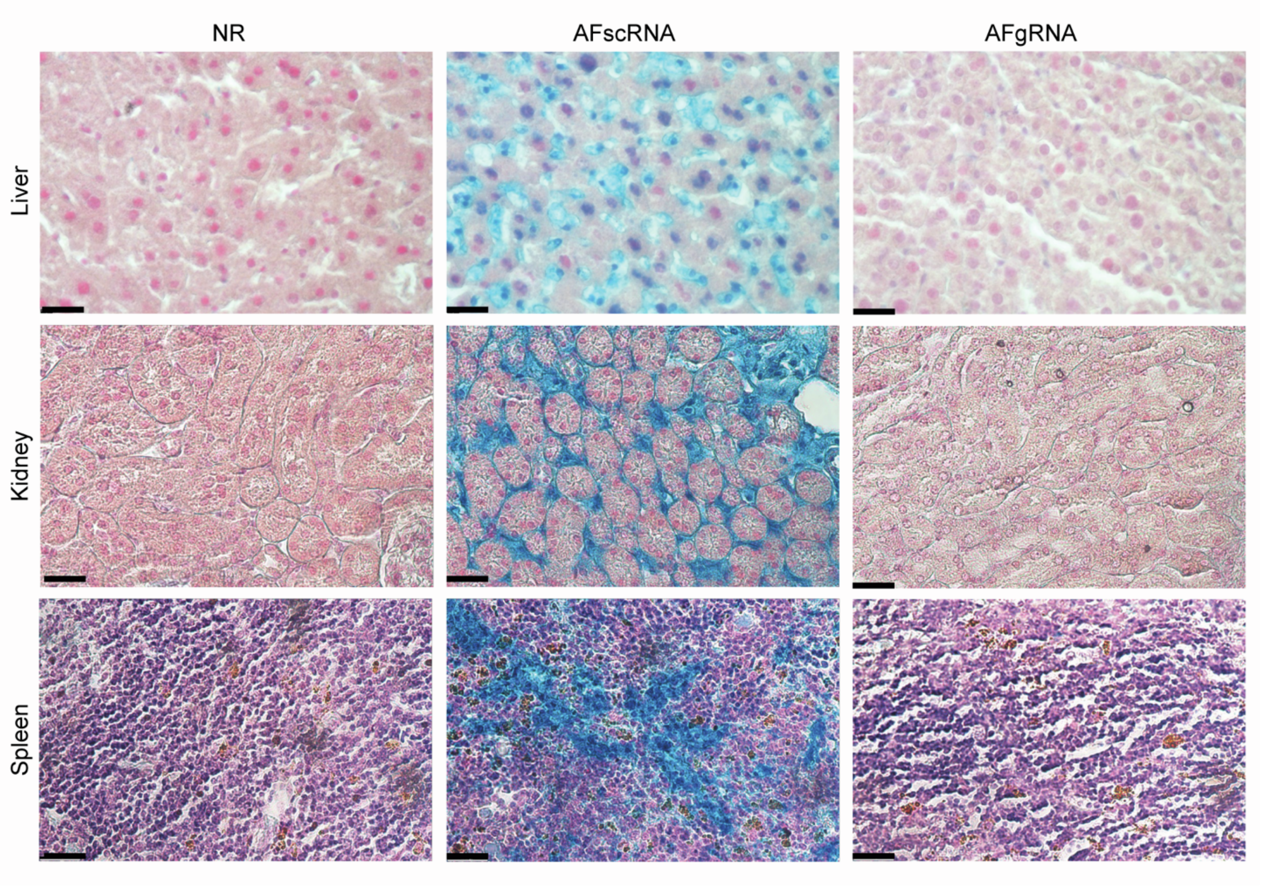

Figure S3. GAGs storage in different AAV-HITI-treated tissues. Related to Figure 3. Alcian blue staining of histological sections from different tissues: liver, kidney, and spleen. Scale bar 50µm. Unaffected untreated mice (NR, N=4); affected MPS VI mice treated with AAV-HITI-scRNA (AFscRNA, N=8); affected MPS VI mice treated with AAV-HITI-gRNA (AFgRNA, N=8).

Figure S4

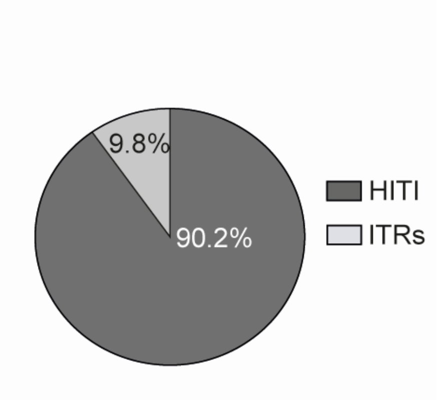

Figure S4. Short reads sequencing of 5' HITI junction from liver genomic DNA. Related to Figure 5. Pie chart showing the percentage (%) of short reads in which donor DNA integration was HITI-mediated or ITRs-mediated (ITRs) at the 5' junction site.

Figure S5

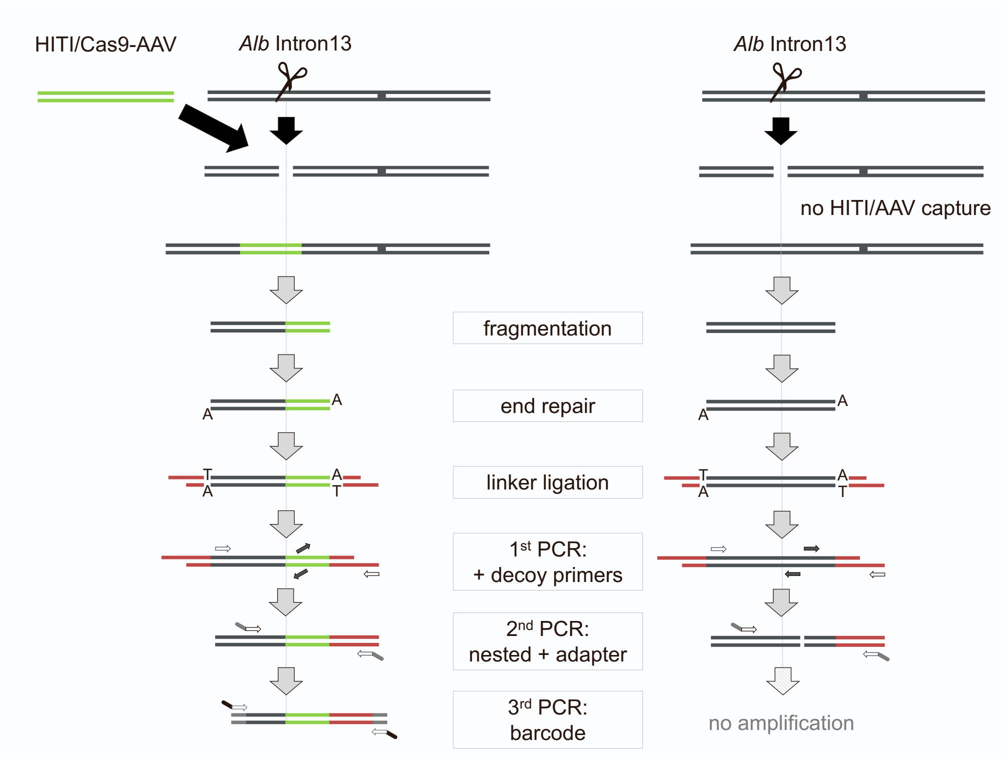

Figure S5. Schematic representation of CAST-Seq at the on-target site. Related to STAR Methods. The cartoon shows the CAST-Seq library preparation for e detection of integrated AAV-HITI vectors at the on-target site upon nuclease cleavage. Liver genomic DNA is fragmented randomly, and end repaired to add a 3′-A overhang which is used for ligation of a short linker (red). The first PCR is performed with bait and prey primers (white arrows) binding to the target site and the linker, along with decoy primers (black arrows) binding to the on-target locus near the cleavage site. The second PCR with nested primers adds adapters that are used in the third PCR to add barcodes.

Figure S6

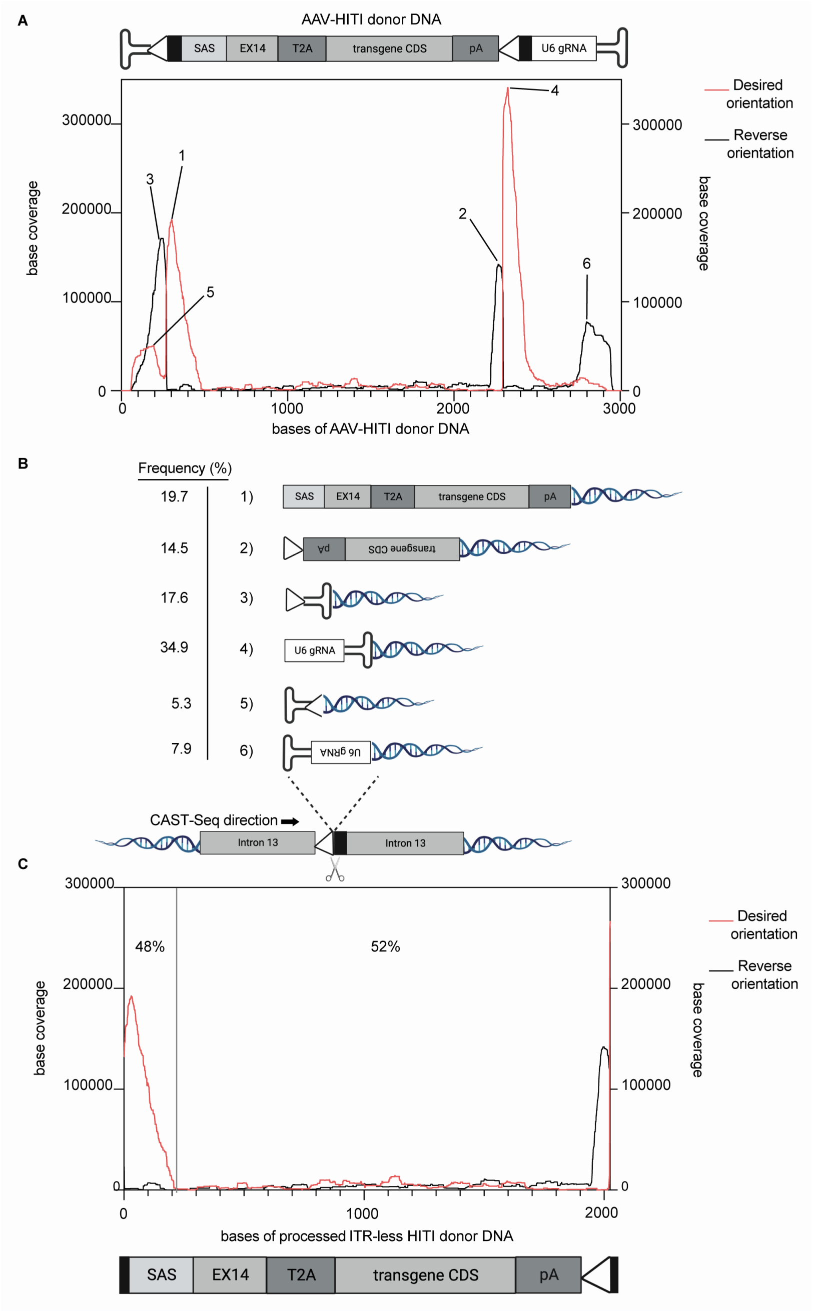

Figure S6. Assessment of HITI integration events at the on-target site by CAST-Seq. Related to Figure 5. A) Single base coverage of integration events by CAST-Seq. For each base, read coverage of the desired (forward strand) and reverse (reverse strand) integration is shown. Labelled are six distinct peaks that represent different integration events. **B**) Schematic representation of integration events. Shown are the six integration events corresponding to the peaks in (**A**), and their relative frequency determined by peak height. **C**) CAST-Seq based single base read coverage of cleaved transgene integration events. For each base, the read coverage of the desired integration and reverse integration is shown. The percentage of functional (left) versus non-productive integration (right) events are indicated.

Figure S7

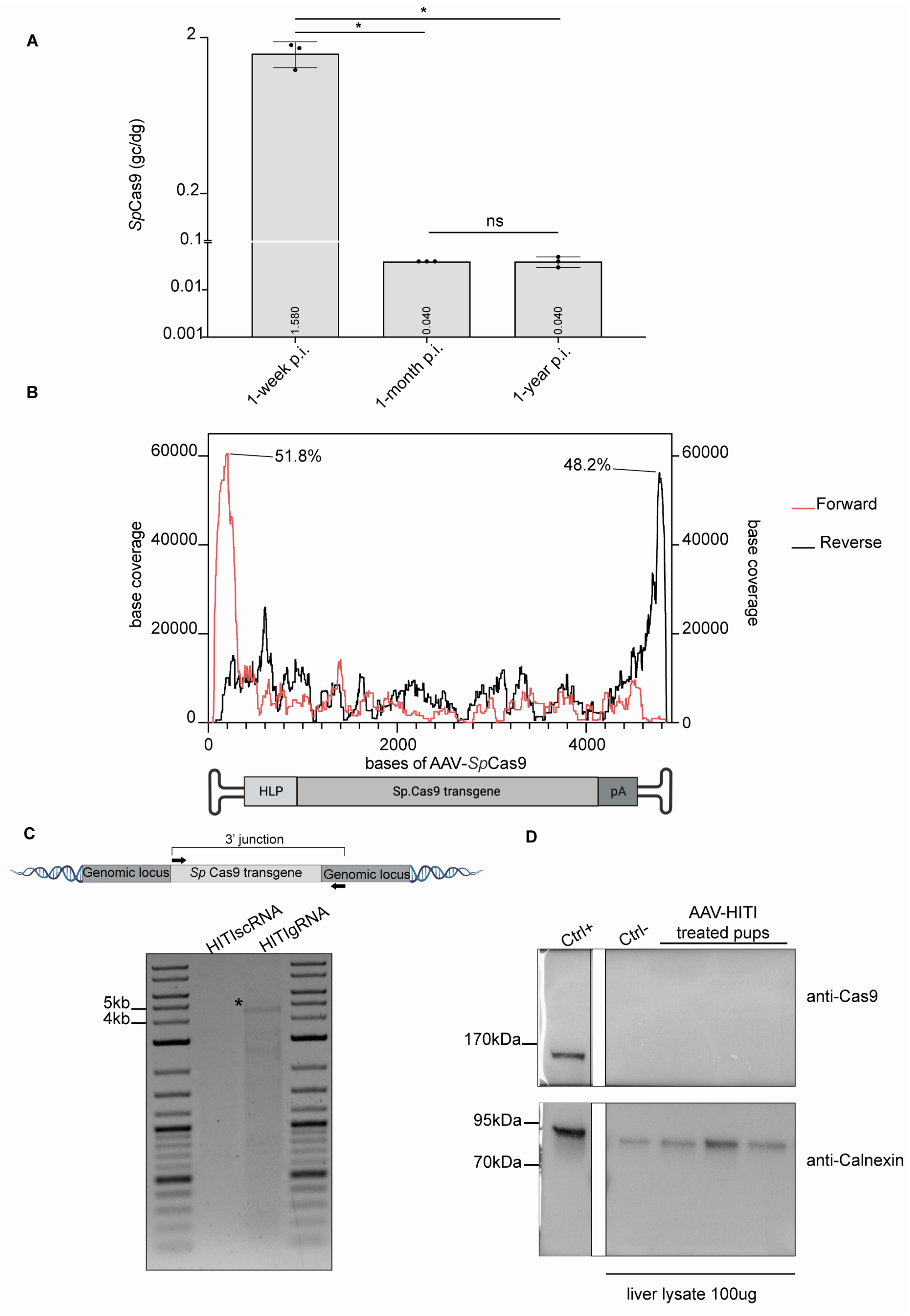

Figure S7. Evaluation of AAV-*Sp*Cas9 biodistribution, integration and expression in liver of newborn AAV-HITI-treated mice. Related to Figure 6. **A**) Real-Time PCR analysis of *Sp*.Cas9 vector genome in liver of AAV-HITIgRNA-treated newborn mice at different timepoints. Data are represented as mean ± standard deviation of Cas9 genome copies/diploid genomes (gc/dg). At each timepoint N=3 samples were used for the analysis. Statistical differences were assessed by Ordinary one-way ANOVA Test followed by Tukey's multiple comparisons test. P-value * = 0.0424

between 1-week and 1-month; p-value * = 0.0183 between 1-week and 1-year; p-value > 0.999 between 1-month and 1-year. **B**) CAST-Seq based single base coverage of AAV-*Sp.*Cas9 integration events. For each base, the read coverage of the forward and reverse strand is shown. The relative frequency of capturing of the 5' and 3' ITR regions as determined by peak height is indicated. **C**) Schematic of the PCR analysis to detect full-length *Sp*Cas9 vector genome integration. Black arrows indicate the primers designed to amplify the 3'junction in the forward orientation: the forward primer was designed to bind in the HA-tag preceding the *Sp*Cas9 transgene; the reverse primer binds in the endogenous locus. The expected product size is ~ 4.5kb. **D**) Western blot analysis performed on liver lysates (100µg) extracted from AAV-HITI-treated mice 1-year after neonatal administration (AFgRNA N=3). Positive control (Ctrl+): cell lysate transiently transfected with a plasmid carrying the *Sp*Cas9. Negative control (Ctrl-): liver lysed obtained from a mouse left untreated.

Figure S8

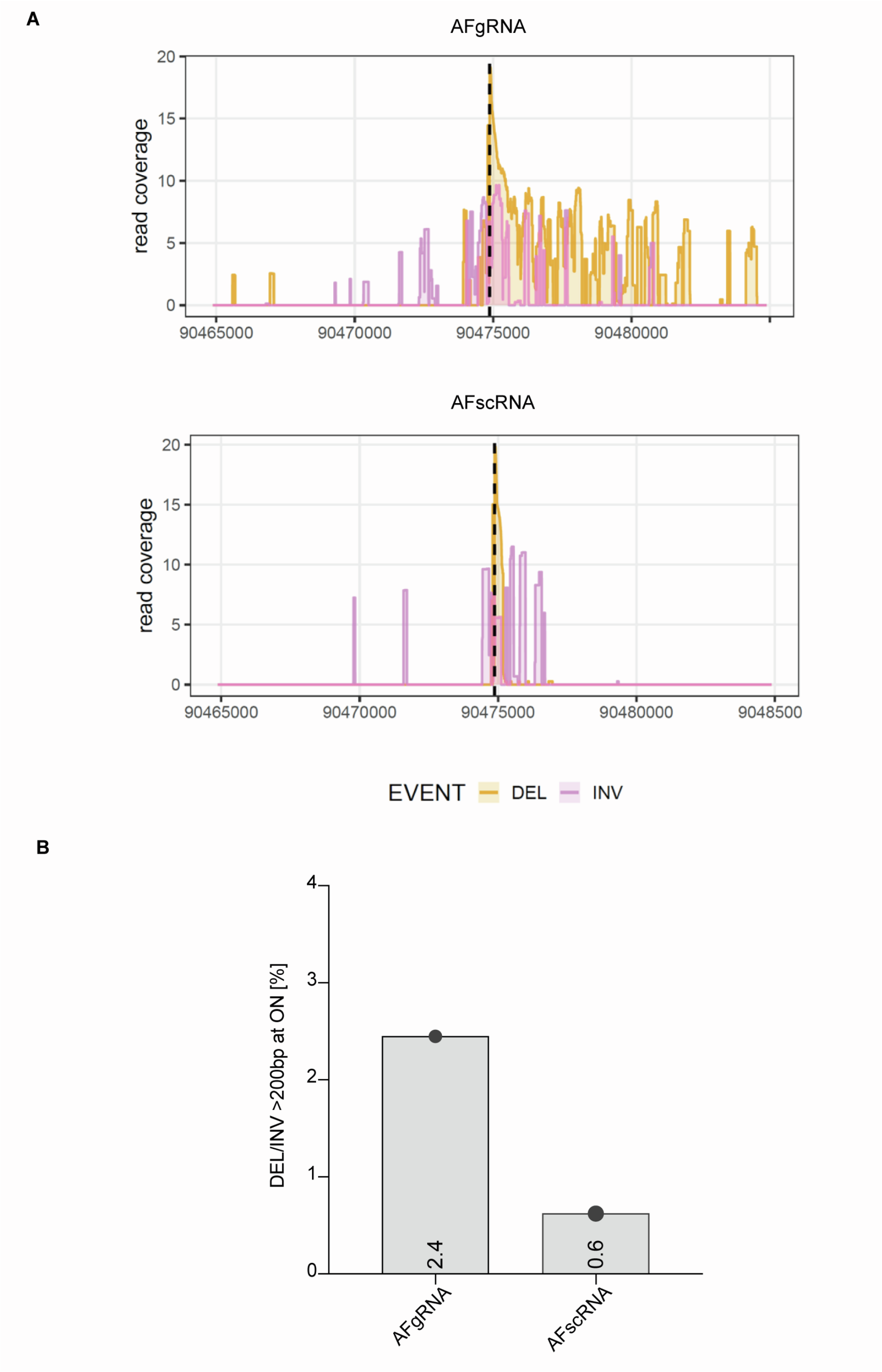

Figure S8. Assessment of on-Target aberrations in liver samples from AAV-HITI-treated mice. Related to Figure 6. A) Coverage plots showing deletions (DEL) and inversions (INV) within +/-10 kb of the on-target site in MPS VI mice treated as newborn with AAV-HITI at high doses (AFgRNA and AFscRNA) as detected by CAST-Seq. **B**) Quantification of CAST-Seq reads in A at a distance of more than 200 bp away from the cut site (ON), indicative of gross chromosomal aberrations such as deletions (DEL) and inversions (INV).

Figure S9

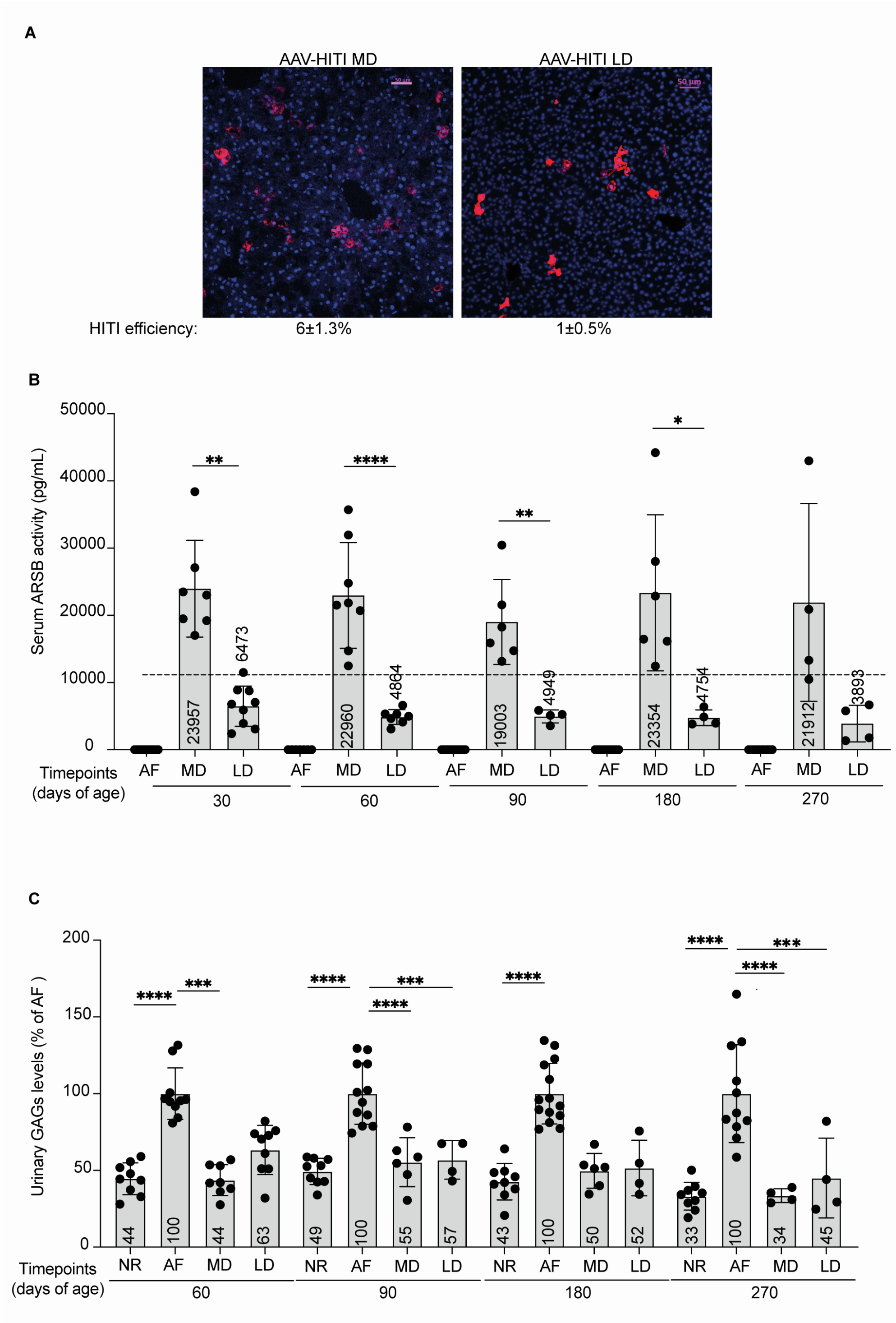

Figure S9. Liver-directed AAV-HITI at various doses in newborn MPS VI mice. Related to Figure 2. A) Representative fluorescence microscopy images of OCT liver cryo-sections from mice injected with AAV-HITI at different doses. Wild-type mice were treated with a medium dose (MD N=5, 3.9x10^13 total GC/kg) or a low dose (LD N=5, 1.2x10^13 total GC/kg) of AAV-HITIgRNA vectors. The percentage of Ds-Red positive hepatocytes (HITI efficiency) is reported. Scale bar 50µm. **B**) MPS VI mice were either left untreated (AF, N=14) or treated with a medium

dose (MD, N= 8) or a low dose (LD, N= 9), as in (**A**), of AAV-HITI-gRNA (AFgRNA) and serum ARSB activity was analysed at different timepoints after administration. Dotted line corresponds to normal serum ARSB activity (11825±334 pg/mL; Alliegro et al., 2016). Statistical differences were assessed by the ordinary one-way ANOVA and Tukey's multiple comparisons test; at p30 p-value ** =0.001; at p60 p-value **** < 0.001; at p90 p-value ** = 0.0052; at p180 p-value * =0.0302. **C**) Urinary GAGs were measured at different timepoints in MPS VI mice treated with either a medium (MD, N=8) or low dose (LD, N=9), of AAV-HITI-gRNA as in (**A**), or left untreated (AF, N=14); NR, N=9, normal mice. Values are reported as a percentage of GAGs levels in untreated MPS VI mice (% of AF). Statistical differences were assessed by Kruskal-Wallis Test and Dunn's multiple comparisons test at p60 p-value **** = <0.0001 between NR and AF; p60 p value ***= 0.0026 between AF and MD. Statistical differences were assessed by Welch ANOVA Test and Dunnett's T3 multiple comparisons test at p90 p-value **** = <0.0001 between NR and AF; p90 p-value ****= 0.0033 between AF and MD; p90 p-value ***= 0.0082 between AF and LD. Statistical differences were assessed by Kruskal-Wallis Test and Dunn's multiple comparisons test at p180. P-value ****= 0.0005 between NR and AF; at p270 p-value ****= < 0.0001 between AF and MD; at p270 p-value ***= 0.001 between AF and LD. Each dot corresponds to a single animal within each group at different time points. **B-C**) The differences in the number of analyzed samples within the same group of treatment at different timepoints was due to sample availability. All Data are represented as mean ± standard deviation.

Figure S10

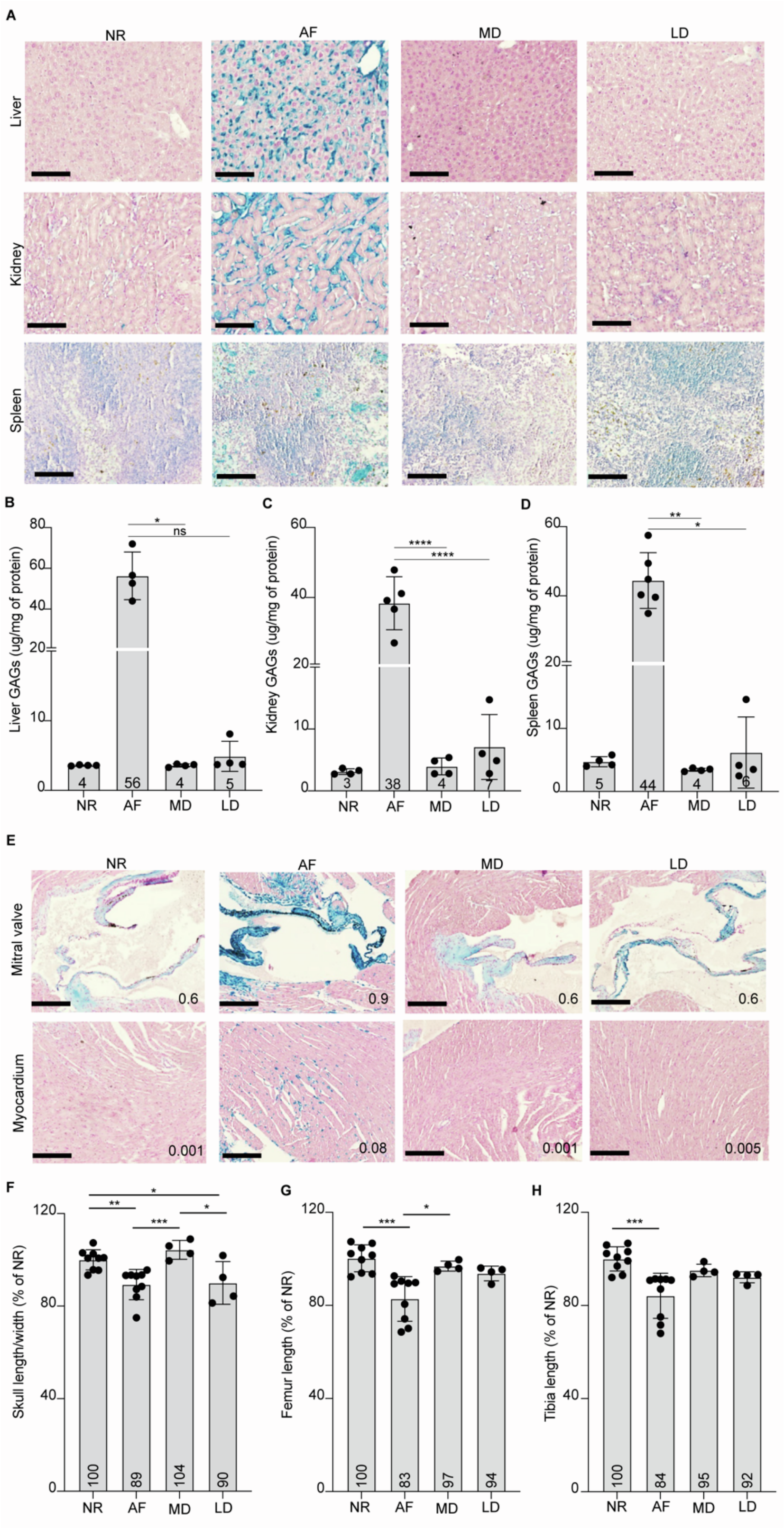

Figure S10. Efficacy of AAV-HITI at various doses in newborn MPS VI mice. Related to Figure 3. A) Alcian blue staining of histological sections from liver, kidney, spleen. Scale bar 50μm. Quantification of GAGs in liver (**B**), kidney

(**C**), and spleen (**D**). NR, unaffected untreated mice; AF (liver N=4; kidney N=5; spleen N=6), affected MPS VI mice, and AFgRNA MD (N=4), affected MPS VI mice treated with AAV-HITI-gRNA at medium doses; AFgRNA LD (N=4), affected MPS VI mice treated with AAV-HITI-gRNA at low doses **E**) Alcian blue staining of histological sections from mitral heart valve and myocardium. Alcian Blue quantifications in the mitral heart valve and in myocardium are reported as Alcian blue positive area/total area. Scale bar 50µm. NR, unaffected untreated mice; AF (N=6), affected MPS VI mice, and AFgRNA MD (N=4), affected MPS VI mice treated with AAV-HITI-gRNA at medium doses; AFgRNA LD (N=4), affected MPS VI mice treated with AAV-HITI-gRNA at low doses. **F-H**) Measurement of skull length/width ratio (**F**), femur (**G**) and tibia (**H**) lengths; data are reported as the percentage of normal length (% of NR). NR, unaffected untreated mice; AF (N= 9), affected MPS VI mice, and AFgRNA MD (N=4,), affected MPS VI mice treated with AAV-HITI-gRNA at medium dose; and AFgRNA LD (N=4,), affected MPS VI mice treated with AAV-HITI-gRNA at low doses. Statistical differences were assessed by ordinary one-way ANOVA and Tukey's multiple comparisons test: **B**) p-value * = 0.0138 between NR and AF; p-value >0.9999 between NR and AFgRNA MD; p-value * = 0.0398 between AF and AFgRNA MD; p-value =0.5656 between NR and AFgRNA LD; p-value >0.9999 between AF and AFgRNA LD; p-value >0.9999 between AFgRNA MD and AFgRNALD . **C**) P-value **** <0.0001 between NR and AF; p-value = 0.9949 between NR and AFgRNA MD; p-value **** <0.0001 between AF and AFgRNA MD; p-value = between NR and AFgRNA LD; p-value = 0.6712 between AF and AFgRNA LD; p-value 0.8037 between AFgRNA MD and AFgRNALD. **D**) P-value = 0.9858 between NR and AFgRNA MD; p-value **= 0.0051 between AF and AFgRNA MD; p-value >0.9999 between NR and AFgRNA LD; p-value* =0.0436 between AF and AFgRNA LD; p-value >0.9999 between AFgRNA MD and AFgRNALD. **F**) P-value** =0.0026 between NR and AF; p-value =0.6948 between NR and AFgRNA MD; p-value*** = 0.0009 between AF and AFgRNA MD; p-value *= 0.0401 between NR and AFgRNA LD; p-value =0.9995 between AF and AFgRNA LD; p-value* =0.0421 between AFgRNA MD and AFgRNA LD. **G**) p-value*** =0.0008 between NR and AF; p-value >0.9999 between NR and AFgRNA MD; p-value *= 0.0394 between AF and AFgRNA MD; p-value >0.9999 between NR and AFgRNA LD; p-value =0.6061 between AF and AFgRNA LD; p-value >0.9999 between AFgRNA MD and AFgRNALD. **H**) p-value*** =0.0001 between NR and AF; p-value >0.9999 between NR and AFgRNA MD; p-value =0.1701 between AF and AFgRNA MD; p-value = 0.3631between NR and AFgRNA LD; p-value >0.9999 between AF and AFgRNA LD; p-value >0.9999 between AFgRNA MD and AFgRNA LD.

Figure S11

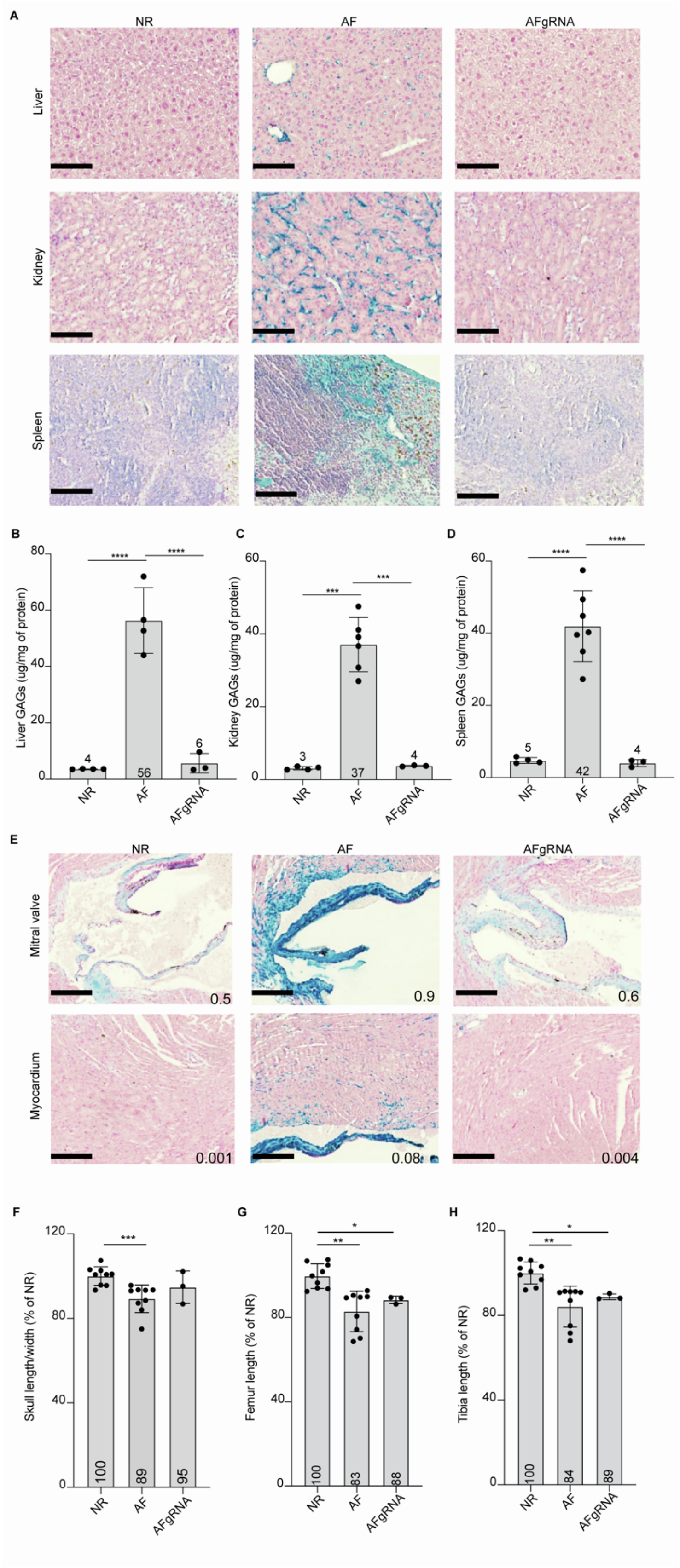
A
NR
AF
AFgRNA
Liver
Kidney
Spleen
B
Liver GAGs (ug/mg of protein)
C
Kidney GAGs (ug/mg of protein)
D
Spleen GAGs (ug/mg of protein)
E
Mitral valve
Myocardium
0.5
0.9
0.6
0.001
0.08
0.004
F
Skull length/width (% of NR)
G
Femur length (% of NR)
H
Tibia length (% of NR)

Figure S11. Efficacy of AAV-HITI in adult MPS VI mice. Related to Figure 7. A) Alcian blue staining of histological sections from liver, kidney, spleen. Scale bar 50μm. Quantification of GAGs in liver (**B**), kidney (**C**), and spleen (**D**). **E**) Alcian Blue quantification in the mitral heart valve and in myocardium are reported as Alcian blue positive area/total area. Scale bar 50μm. **F-H**) Measurement of skull length/width ratio (**F**), femur (**G**) and tibia (**H**) lengths; data are reported as the percentage of normal length (% of NR). NR (N=9), unaffected untreated mice; AF (N=9), affected MPS VI mice, and AFgRNA (N=3), affected MPS VI mice treated with AAV-HITI-gRNA at low doses; In **B**) NR, N=4; AF N=4; AFgRNA, N=3. In **C**) NR, N=4; AF N=6; AFgRNA, N=3. In **D**) NR, N=4; AF N=7; AFgRNA, N=3. Statistical differences were assessed by ordinary one-way ANOVA and Tukey's multiple comparisons test. **B)** P-value **** <0.0001 between NR and AF; p-value = 0.9280 between NR and AFgRNA; p-value **** <0.0001 between AF and AFgRNA. **C**) P-value ***= 0.0003 between NR and AF; p-value = 0.1385 between NR and AFgRNA; p-value ***= 0.0003 between AF and AFgRNA. **D**) P-value **** <0.0001 between NR and AF; p-value = 0.9904 between NR and AFgRNA; p-value **** <0.0001 between AF and AFgRNA. **F**) p-value *** =0.00029 between NR and AF; p-value = 0.3868 between NR and AFgRNA; p-value = 0.3610 between AF and AFgRNA. **G**) p-value** =0.0014 between NR and AF; p-value * = 0.0322 between NR and AFgRNA; p-value >0.9999 between AF and AFgRNA. **H**) p-value **=0.0012 between NR and AF; p-value * =0.0235 between NR and AFgRNA; p-value >0.9999 between AF and AFgRNA.

Table S2

Primers	Sequence (5'-3')
**5' junction forward**	CACGTGGTCAGGTGTAGCTC
**5' junction reverse**	TGGAGAGAAAGGCAAAGTGGA

Table S2. 5' short junction primers. Related to Figure 4 and STAR Methods. Primers used to PCR amplify the 5' junction upon donor DNA integration.

Table S3

Primers	Sequence (5'-3')
**Nanopore LR-Seq 5' junction forward**	CCACCAATCTAGATGTTGTTC
**Nanopore LR-Seq 5' junction reverse**	CCCAGAATAGAATGACACCTACTCA
**Nanopore LR-Seq 3' junction forward**	**GAGTCTTGTGTCCCAGTTACCAGG**GGTCTTACTGACATCCACTTTG
**Nanopore LR-Seq 3' junction reverse**	**CCCTGGGACGTAGGAATCCACGCC**GGTAACTGTCCATTCATGTG

Table S3. Nanopore long-read (LR) sequencing primers. Related to Figure 5 and STAR Methods. Sequences in bold are barcodes to enable multiplexed sequencing on the same flow cell.

Table S4

Primers	Sequence (5'-3')
**3' junction forward**	CCCATACGATGTTCCAGATTAC
**3' junction reverse**	GGTAACTGTCCATTCATGTG

Table S4. *Sp*Cas9 3'junction primers. Related to STAR Methods. Primers used to PCR amplify the 3' junction to assess full-length *Sp*Cas9 integration.

Table S5

Target	Function		Sequence (5'-3')
mAlb-HITI	CAST-Seq PCRI	bait	CCACCAATCTAGATGTTGTTC
	CAST-Seq PCRI	decoy fwd	GACACAGAAGAGCATAGTTAGA
	CAST-Seq PCRI	decoy rev	CAAATTTGAAACCAAATAGTGATAATAGG
	CAST-Seq PCRI	decoy HITI	GATGTCAGTAAGACCAATAGGTG
	CAST-Seq PCRII	bait nested	GACTGGAGTTCAGACGTGTGCTCTTCCGATCTGTTGTTCTCTACGTAACTGTAATAGAAAC
Linker primers	CAST-Seq PCRI	prey	GTAATACGACTCACTATAGGGC
	CAST-Seq PCRII	prey nested	ACACTCTACACTCTTTCCCTACACGACGCTCTTCCGATCTAGGGCTCCGCTTAAGGGAC
Linker oligos	CAST-Seq positive strand		GTAATACGACTCACTATAGGGCTCCGCTTAAGGGACT
	CAST-Seq negative strand		P-GTCCCTTAAGCGGAGC-NH3

Table S5. CAST-Seq primers. Related to Figure 6 and STAR Methods. Primers used to perform CAST-Seq.

Table S6

gRNA	Region	Position	(5'-3') gRNA + PAM sequences	Mismatches	Off-target CDF score
ON- target	Intron: albumin	chr5: 90622727-90622747:-	GTATTTAATAGGCAGCAGTG TGG	-	-
OFF-target 1	Intron: Rik	chr5:151333345-151333367:-	**T**TA**C**TTAATA**A**GCAGCAGTG TGG<br>* * *	3	0.647
OFF-target 2	Intron: Lrr 1	chr12: 69224137-69224159:-	GT**T**TTTAA**A**A**A**GCAG**A**AGTG GGG<br>* * * *	4	0.646
OFF-target 3	Intergenic: Ppp1r3c-Tnks2	chr19: 36774036-36774058:-	**T**TAT**C**TAATAG**A**CAGCA**A**TG CGG<br>* * * *	4	0.646
OFF-target 4	Intron: Zim2	chr7: 6660686-6660708:+	G**A**ATTT**G**ATAG**A**CAGCAGTG GGG<br>* * *	3	0.557
OFF-target 5	Intron:Slc39a12	chr2:14426612-14426634:+	GTATTTA**GA**AGGCAGCAGT**T** TGG<br>** *	3	0.476
OFF-target 6	Intron: Gstcd	chr3:132751735-132751757:-	**AA**ATTT**G**AT**T**GGCAGCAGTG TGG<br>** * *	4	0.474
OFF-target 7	Intron: Kcnc1	chr7: 46060812-46060834:-	GTATTTAA**A**AGGC**T**G**A**AGT**A** AGG<br>* * * *	4	0.464
OFF-target 8	Intergenic: Rik/Lhfpl3-Lhfpl3	chr5: 23268978-23269000:+	**A**TATT**C**AA**GT**GGCAGCAGTG AGG<br>* * **	4	0.446
OFF-target 9	Intron: Dpyd	chr3:119141669-119141691:+	**A**TATTTAATAGGCA**A**CA**T**T**T** AGG<br>* * * *	4	0.395
OFF target 10	Intron: HCn2	chr10: 79561901-79561923:+	G**G**ATT**C**A**G**TAGGCAGCAGT**T** GGG<br>* * * *	4	0.392

Table S6. Off-Targets. Related to Figure 6. The top-ten predicted off-target sites were selected with the CRISPOR web tool. The mouse genome used of reference is the (GRCm39/mm39).

Table S7

Primers	Sequence (5'-3')
Off-target 1 forward	TGTGGTTGCTGGGATTTGAAC
Off -target 1 reverse	ACTCCTCAGAAGGGTAATTGTAG
Off -target 2 forward	CACAGTAGGAATGCCATAGAAG
Off -target 2 reverse	TGTGGTAGGTAGGGAAAGAAC
Off-target 3 forward	GACTCCTTCACAAGGGATCACTTG
Off-target 3 reverse	AGTGTTCCAGTCTAAAGCACT
Off-target 4 forward	GCATGCTTAACTGCTGAGGT
Off-target 4 reverse	AGTAAGCTACACATGTAACAGCGACTAG
Off-target 5 forward	AATGCTGCAGGTCTGAGTGA
Off-target 5 reverse	ATAACTTCCGAGGTCTACTGCT
Off-target 6 forward	TGTAAAGTTAGCTGCTGACTG
Off-target 6 reverse	CTAACATCAACTTCAAGCACAGGAAG
Off-target 7 forward	TTGCTTGCTGTTTCCTGTGA
Off-target 7 reverse	CTCTGAGGTGTACTTTGTACCT
Off-target 8 forward	ACCCTCCCCTGTGACTTACT
Off-target 8 reverse	CCACCCTACTAAGCTATGCT
Off-target 9 forward	GAATAAGATGAGGCCAGAGTA
Off-target 9 reverse	TTCACGTTCATCCAAGGTC
Off-target 10 forward	AGGACTTGGGCAGGATAAA
Off-target 10 reverse	TGTGACCATGAGAGTCCCAG

Table S7. Off-target primers. Related to Figure 6. List of the primers used to PCR amplify the predicted off-target loci.
